# The precise timeline of transcriptional regulation reveals causation in mouse somitogenesis network

**DOI:** 10.1186/1471-213X-13-42

**Published:** 2013-12-05

**Authors:** Bernard Fongang, Andrzej Kudlicki

**Affiliations:** 1 Department of Biochemistry and Molecular Biology, Sealy Center for Molecular Medicine, Institute for Translational Sciences, University of Texas Medical Branch, 301 University Blvd, Galveston, TX, 77555, USA

**Keywords:** Somitogenesis, Transcriptional regulation, Maximum Entropy deconvolution

## Abstract

**Background:**

In vertebrate development, the segmental pattern of the body axis is established as somites, masses of mesoderm distributed along the two sides of the neural tube, are formed sequentially in the anterior-posterior axis. This mechanism depends on waves of gene expression associated with the Notch, Fgf and Wnt pathways. The underlying transcriptional regulation has been studied by whole-transcriptome mRNA profiling; however, interpretation of the results is limited by poor resolution, noisy data, small sample size and by the absence of a wall clock to assign exact time for recorded points.

**Results:**

We present a method of Maximum Entropy deconvolution in both space and time and apply it to extract, from microarray timecourse data, the full spatiotemporal expression profiles of genes involved in mouse somitogenesis. For regulated genes, we have reconstructed the temporal profiles and determined the timing of expression peaks along the somite cycle to a single-minute resolution. Our results also indicate the presence of a new class of genes (including Raf1 and Hes7) with two peaks of activity in two distinct phases of the somite cycle. We demonstrate that the timeline of gene expression precisely reflects their functions in the biochemical pathways and the direction of causation in the regulatory networks.

**Conclusions:**

By applying a novel framework for data analysis, we have shown a striking correspondence between gene expression times and their interactions and regulations during somitogenesis. These results prove the key role of finely tuned transcriptional regulation in the process. The presented method can be readily applied to studying somite formation in other datasets and species, and to other spatiotemporal processes.

## Background

The mechanism of segmentation of the vertebrate embryo depends on waves of gene expression progressing through the presomitic mesoderm (PSM) along the antero-posterior axis of the body [1] and involves regulation of genes from the Notch, Fgf and Wnt pathways [2,3]. Perturbations of this process cause congenital vertebral malformations (CVMs) [4,5], however, in many cases their mechanism is not known, although impressive amounts of experimental profiles including whole-genome expression data have been collected [1-3,6-8]. According to the “clock-and-wavefront” model [9], the periodicity of somitogenesis is governed by a molecular oscillator that drives waves of gene expression caudal-rostrally through the PSM. The evidence for cycling genes was first observed in the chick PSM [10] where *c-hairy1* displays dynamic wave of *mRNA* expression caudal-rostrally and has been subsequently extended to other species as zebrafish [11-13] and mouse [1,14]. Mice carrying mutations in genes encoding ligands, receptors or downstream effectors of the Notch pathways display severe segmentation defects [14-16]. It is therefore believed that Notch pathway is a crucial component of the vertebrate segmentation mechanism. Indeed, known Notch cycling genes in mammalian somitogenesis include: *Hes1/7/5*, *Hey1/3*, *Lfng*, *Nkd1*, *Nrarp*, *Maml3*, *Bcl9l*[15]. Also the Wnt signaling pathway is also rhythmically activated in the PSM and reported cyclic genes from this pathway include *Axin2*, *Dact1*, *Dkk1*, *Sp5*, *Tnfrsf19*, *Myc*, *Has2*, *Phlda1*. It has been reported that inactivation of Wnt inhibitors such as *Dkk1*, results in segmentation defects [13,15-18]. Other known cycling genes like *Spry2/4*, *Dusp6*, *Shp2, Hspg2, Efna2, Bcl2l11* belong to the Fgf family.

The details of the wave mechanism, the core pacemaker as well as the hierarchy between the components of Notch, Fgf and Wnt and other pathways involved remain largely unknown. Although all three pathways appear essential to proper functioning of the segmentation clock, there is no consensus whether the central oscillator is directly driven by periodic activation of the Notch, Wnt or Fgf pathways, or, conversely, are these pathways regulated by an oscillator acting upstream of them (see [19-21] and the excellent review in [22]). Analysis of high-resolution gene expression profiles, including precise timing of gene expression may facilitate identification of further components of the network, causal relations between them as well as the transcriptional regulatory elements associated with each gene thus improving our understanding of the molecular mechanisms involved in somite formation. The segmentation clock is believed to be conserved between species; however some of the genes and regulations involved vary between the clades [13]. Therefore, comparison between the process in different organisms may shed light on the evolution of the process, and allow identification of the most conserved, primordial aspects.

The activity of each of the three pathways is confined to a specific phase of the somite cycle. Precisely timed transcriptional regulation plays a role in processes outside development, e.g. cell division, metabolic oscillation, biogenesis of organelles. Tight confinement of transcription of genes to a specific time interval may be beneficial for several reasons. First, it allows compartmentalization in time and prevents interactions between incompatible biochemical processes [23]. Second, by just-in-time transcription, the organism does not need to store and maintain proteins when they are not used. Third, when the order of gene transcription follows the order of recruitment of subunits to a protein complex, proper assembly of the complex is facilitated [24]. It is therefore natural to postulate that in somitogenesis, the timing of gene expression will reflect the order in which the gene products enter their specific pathways. Because causation cannot act backward in time, reconstructing the order of events is an important step towards uncovering the causal dependencies between the particular elements of a biological network. Two traditional approaches to extracting the timeline of expression from timecourse experiments are: using the time of the highest measurement and computing the phase of the best-fit single harmonic wave. In the highest-peak method, the resolution is inherently limited to that of the source data, which is typically low due to the high cost of microarray experiments. The method is sensitive to experimental error or noise: just one bad measurement may result in drastically altering the timing result. The phase of the main Fourier mode [25] is more resistant to noise, without limiting the resolution; however, this will produce accurate results only if the data are well-approximated by a single sinusoid, which is often not the case.

The nature of microarray data, where the sample may contain subpopulations of cells in different states, or at different stages of the cycle, has allowed introduction of a new method, based on algebraic decomposition of the profile into a series of profiles for each of the sub-populations [26]. The measurement at a given time, *M(t)*, is considered to be a sum of measurements for different samples: *M*(*t*) = *a*_1_*M*_1_(*t*) + *a*_2_*M*_2_(*t*) + … + *a*_*k*_*M*_*k*_(*t*). For a continuous family of sub-populations, that differ only by a time shift, the sum takes the form of an integral: *M*(*t*) = ∫ *E*(*t* − *τ*)*h*(*τ*)*dτ*, where *E* is the true underlying profile, and *h* is the distribution of cycle phases among the cells in the experimental sample. It has been shown [24] that as long as the probability distribution of time-shifts *h* is known, then by using a deconvolution algorithm based on prior probability derived from maximum entropy principle, one can solve this equation reconstructing the underlying source signal *E* with a resolution even 10 times better than that of the source data. The procedure automatically filters out most of the noise in the data because it favors regulations consistent with the underlying model *h*. Here, we report a version of the method tailored to processes where the expression levels depend on both time and spatial coordinates.

Over time, cells in every location along the PSM pass through all phases of the somite cycle. Moreover, at any given moment in time, cells in different positions along the PSM will be in different phases of the cycle, as a consequence of the fact that the gene expression wave travels along the body axis. Therefore, the experimental sample (containing cells from different locations) will contain cells in different phases of the cycle, resulting in an artificially blurred expression profile. The present, spatiotemporal version of the deconvolution formalism is designed to compensate for this effect and computationally reconstruct the original profile, free from experimental artifacts. The fundamental difference between the temporal and spatiotemporal deconvolution lies in constructing the kernel (blur) function. In the time deconvolution [24], the kernel *h* was constructed based on the distribution of times, at which the cells enter the cycle. In the present, spatiotemporal case, *h* is derived from the known geometry of the embryo-sample system, and the velocity of the expression wave.

Deconvolution in space and time thus allows reconstructing the underlying expression profile. Knowing the profile, we are able to determine the time of peak expression of a gene with the resolution of several minutes, which is the time-scale at which the transcriptional regulation is optimized (at shorter timescales the time spent on gene translation and posttranslational modifications may have an effect). We use the method to reconstruct the spatiotemporal expression profiles in mouse somitogenesis. Peaks in the profiles precisely indicate the timing of gene regulation and their sequence reveals details of the finely-tuned regulatory network. As we shall see, the time of gene expression is tightly related to the time of activity of a gene’s product, even if further, posttranscriptional and posttranslational steps of regulation are required. We postulate that this prevalence of just-in-time expression [24] allows the cell to economize on storage and maintenance of proteins not being used at a given time.

## Results and discussion

We have developed and implemented a dedicated suite of algorithms that assign the correct phase of the cycle to each data point, characterize the dependence between time, position and cycle phase, perform the deconvolution to reconstruct the full spatiotemporal profile, determine the phase of expression peak, and estimate the accuracy and resolution of the resulting timing (see Methods). The algorithms applied to the data of [2] and independently to the data from [13] reveal and confirm the intricate sequence of transcriptional events associated with the somite cycle, which is the main result of the presented research.

### Accurate cycle phase for collected data points

Previous analysis [2] has assumed evenly spaced embryos along the clock cycle, which corresponds to an approximately 7 minute difference between consecutive time points in their data. Instead of using this crude assumption, we use a three-step algorithm to infer the actual phase. First, we analyze the In-Situ Hybridization (ISH) images of [2,13] to measure the position of the highest density of the *Lfng* concentration. Next, the position *x* is converted into an approximate phase *φ* using the formula *x = 0.978(φ+t)*^*0.526*^ derived from a model of wave propagation and its deceleration near the anterior end of the PSM (see Methods). The phases are further refined by Powell optimization [27] of periodicity scores of six strongly regulated benchmark genes (*Hes1, Hes5, Hey1, Lfng, Axin2*); see Methods.

### Deconvolution algorithm

**Figure 1:**
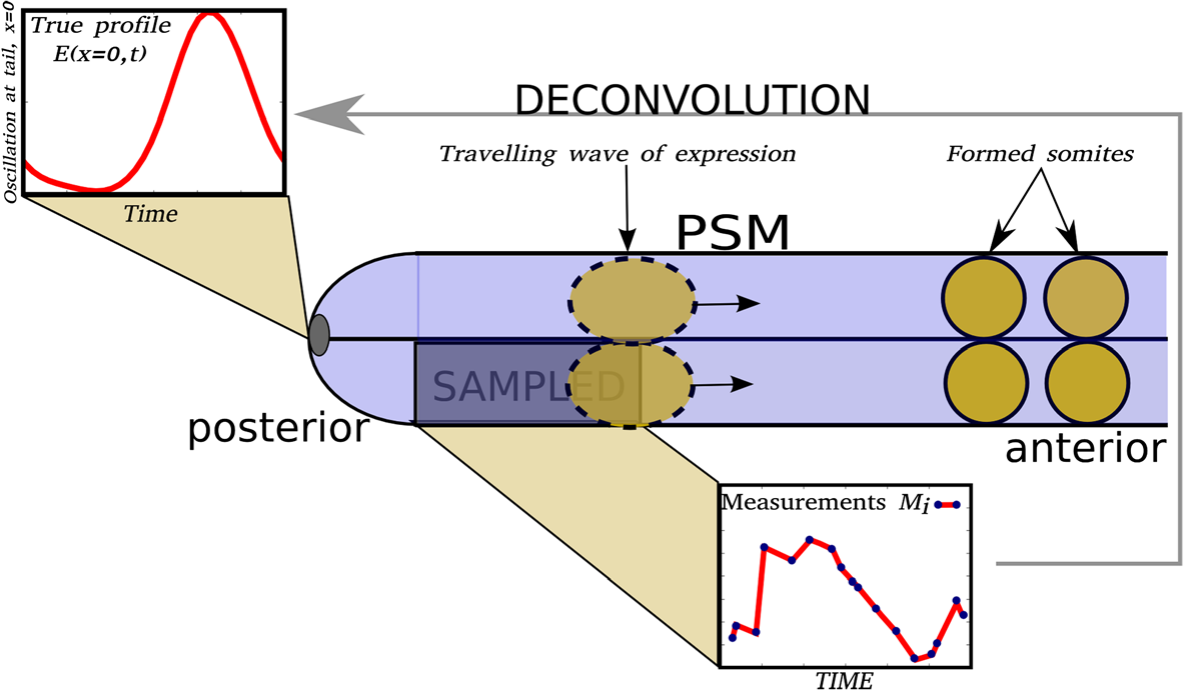
**PSM of the mouse embryo.** The sample used in microarray analysis (right posterior half of the embryo; dark blue) contains cells at different phases of the somite cycle, which distorts the observed expression profile. The deconvolution algorithm is used for reconstructing the true profile representing the oscillation of the gene at the tail *E(x = 0,t)*, from the microarray measurements *M*_*i.*_ at the tail. The measured profile M_i_ is broadened due to the large volume of the sampled region.

In somitogenesis gene expression studies, the mRNA concentration is measured in a sample encompassing a large fraction of the PSM. The sample contains cells in different phases of the somite cycle (see Figure 1) and consequently each measurement can be represented as spatial convolution of the spatiotemporal profile at a given moment in time. The complete spatiotemporal profile of expression can be reconstructed if we assume that the expression *E* depends on the phase of the cycle, *φ*, which is in turn a function of time and position along the AP axis of the embryo. The reconstruction of the most likely spatiotemporal profile uses a prior distribution derived from the Maximum-Entropy principle and solves the integral equation through multidimensional optimization in the phase space (see Methods). Our implementation has been successfully applied to the data of [2] and data of [13] resulting in regular, high-resolution spatiotemporal profiles.

### Spatiotemporal profiles and peaks of regulated genes

**Figure 2:**
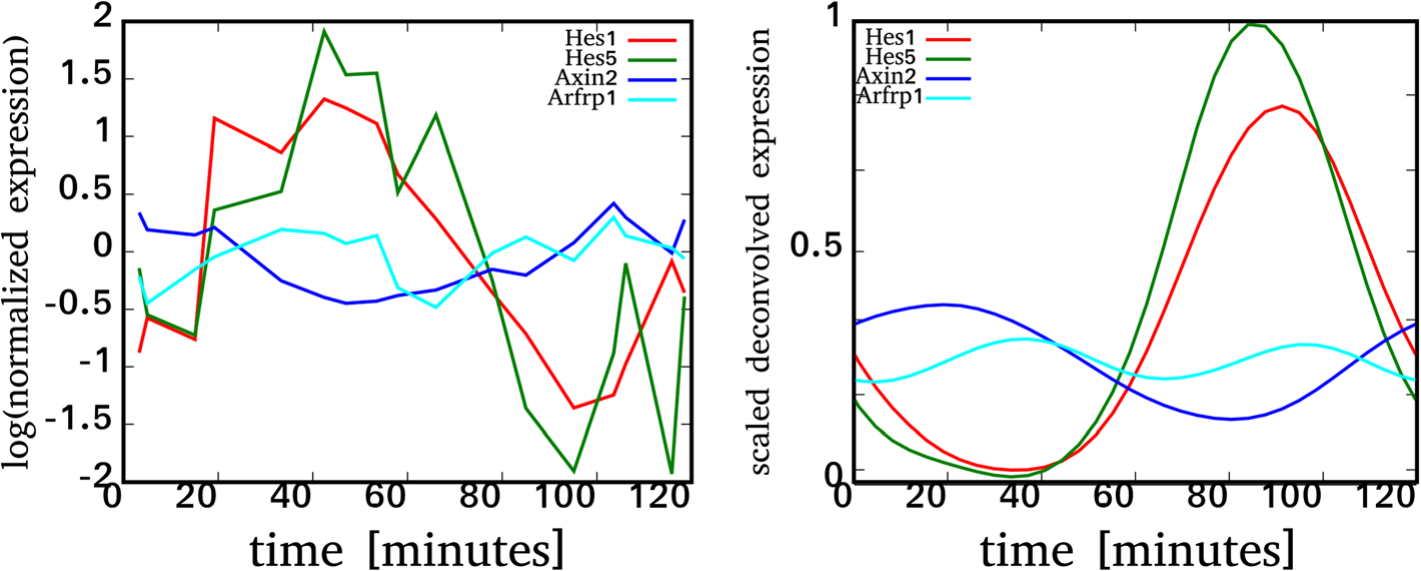
**Original measurements (left) and deconvolved (right) profiles for*Hes1*,*Axin2*,*Hes5*and*Arfrp1*.** The bimodal expression profile of *Arfrp1* shows distinct expression peaks in opposite phases of the somite cycle. The measurements are taken as the average of the posterior half of the PSM, while the reconstructed, deconvolved profile represents the gene expression at the embryo’s tail, hence the overall shift in expression times.

We applied the spatiotemporal deconvolution algorithm to the gene expression data of [2]. These published data are genome-wide mRNA concentrations in the tails of 17 mouse embryos at different stages of the oscillation generating a new somite. This dataset is based on the Affymetrix GeneChip M0E430A microarray platform, which covers a large number of transcripts regulated during somitogenesis. Krol et al. [13] have subsequently collected a second data series in a similar experiment; here we will refer to it as “*mouse-2*”. We use the data of [2] as the primary source of expression profiles, while the deconvolved profiles from *mouse-2*[13] serve as independent validation experiment. Figure 2 represents a comparison between original and deconvolved profiles using *Hes1, Hes5, Afprp1* and *Axin2* as examples*.* The timing of most notable genes, including previously known cyclic genes, is presented in Table 1 and their expression profiles are shown in Additional file 1: Figure S1. All reported times are scaled assuming a 120-minute somite cycle, and relative to the beginning of the cycle defined as the moment when a new *Lfng* band appears at the posterior end of the PSM. The accuracy of timing is assessed for each individual gene using a Monte-Carlo simulation (see Methods), the estimated median error of peak detection is 3 minutes.

**Table 1:** Timing of the most notable cyclic genes with one peak of expression per cycle

**Probeset**	**Gene**	**Time (min)**	**LS p-value**
1420360_at	*Dkk1*	22 **±** 2	0.00017
1427600_at	*Tnfrsf19*	26 **±** 2	0.00022
1436845_at	*Axin2*	20 **±** 2	0.00040
1418102_at	*Hes1*	88 **±** 3	0.00086
1417937_at	*Dact1*	31 **±** 3	0.00097
1422914_at	*Sp5*	23 ± 2	0.00114
1430111_a_at	*Bcat1*	50 ± 6	0.00122
1424942_a_at	*Myc*	20 ± 2	0.00185
1417065_at	*Egr1*	88 ± 5	0.00201
1456010_x_at	*Hes5*	80 ± 3	0.00226
1425895_a_at	*Id1*	82 ± 4	0.00236
1437666_x_at	*Ubc*	19 ± 1	0.00248
1416029_at	*Klf10*	97 ± 6	0.00279
1454904_at	*Mtm1*	80 ± 2	0.00310
1415999_at	*Hey1*	64 ± 4	0.00325
1436584_at	*Spry2*	82 ± 4	0.00510
1417985_at	*Nrarp*	65 ± 3	0.00529
1416895_at	*Efna1*	80 ± 6	0.00629
1456005_a_at	*Bcl2l11*	101 ± 3	0.00697
1449169_at	*Has2*	24 ± 3	0.00952
1418835_at	*Phlda1*	30 ± 2	0.01106
1419180_at	*Bcl9l*	98 ± 8	0.01111
1416039_x_at	*Cyr61*	28 ± 2	0.02952
1448985_at	*Dusp22*	7 ± 1	0.01998
1420643_at	*Lfng*	76 ± 1	0.02458

Times in minutes are scaled to a 120-minute somite cycle.

In the primary analysis of the results, we have considered only genes previously known to be involved in the process and used a peak detection algorithm for proper timing. The peaks of gene expression fall into two main time intervals. The first interval contains mostly genes from the Wnt pathway and their activities are limited to the first 38 minutes of the process. Those Wnt genes may be regulated by *beta-catenin (~5 min:* activated approximately 5 minutes after the beginning of the somite cycle*)* and include *Myc (~20 min), Axin2 (~20 min), Sp5 (~23 min), Dkk1 (~22 min), Has2 (~24 min), Tnfrsf19 (~20 min), Phlda1 (~30 min)*, and *Dact1 (~31 min)*.

The second interval, approximately between the 50^th^ and 100^th^ minutes of the cycle, marks the activity of the Notch and Fgf pathways. Genes from the Notch pathway, which are believed to be activated by *Notch1,* include *Nrarp (~65 min), Nkd1 (~64 min), Hey1 (~64 min), Hes7 (~71 min), Hes5 (~80 min), Lfng (~76 min)* and *Hes1 (~88 min*). Examples from the Fgf pathway include *Spry2 (~82 min), Egr1 (~88 min), Hspg2 (~94 min), Dusp1 (~100 min), Bcl2l11 (~101 min)* and *Shp2 (~80 min).* The separation between the two main phases confirms previous results suggesting that Notch and Fgf-related cyclic genes oscillate mostly in opposite phase to Wnt [2,13,28-30]. Moreover, genes in the Notch pathway are regulated before the Fgf pathway, suggesting that Notch may be acting upstream of Fgf. This difference is statistically significant at *p = 0.0364* (t-test) for peaks of the Notch genes (including *Notch1*) preceding the Fgf genes. This global picture remains unchanged when the expression times are derived from deconvolving the profiles from the Mouse-2 dataset (the peak times of previously reported cyclic genes associated with Wnt, Notch and Fgf pathways are listed in Additional file 2: Table S1).

### Peak times follow causation in regulatory networks

**Figure 3:**
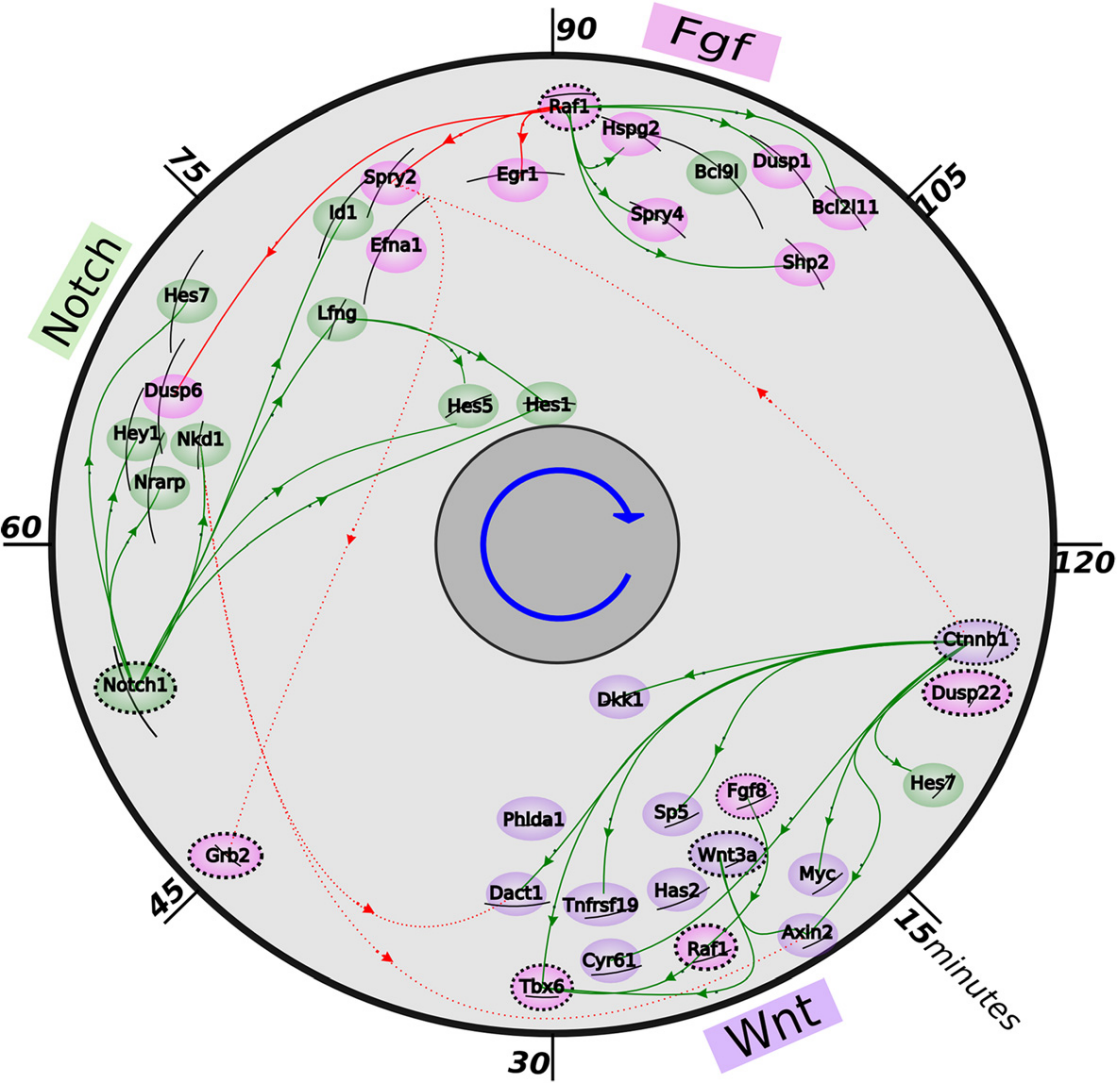
**Gene regulation during mouse somitogenesis.** Position of a gene symbol on the plot reflects time of peak expression (angle; clockwise) and the mean expression level (genes with high expression level are closer to the center). Arrows represent the known causation (green arrows connect genes in the causation direction matching those found in the literature and red arrows are in the reverse causation order. Dot links are between genes too distant to indicate direction of causation). Solid black arcs represent the estimated timing accuracy for each gene. Causation directions are compiled from literature [1-10]. Genes are color-coded according to their known pathway association with green for Notch, magenta for Fgf, purple for Wnt; in addition, dashed strokes are used for genes previously not reported as cyclic.

Knowing the directionality (or causality) of interactions is crucial for understanding of molecular and regulatory mechanisms underlying a biological process. Spatiotemporal waves imply strict correspondence between time and position, and reversing order of events within a cycle is equivalent to reversing direction of traveling wave. In somitogenesis, it is almost impossible to reverse the direction of the wave through microsurgical manipulation, which proves that the paraxial mesoderm cells are endowed with the information for periodicity and directionality very early as they emerge after gastrulation [29,31]. Here, we inferred the probable directions of causal interactions between genes based on the premise, that a gene that is active at a given moment in time can activate genes used shortly afterwards, but can never influence genes activated beforehand. Given the cyclic nature of somitogenesis, we consider two genes as expressed one after another if their expression peaks appear within 45-minutes from one another. We have built a network of directionality based on expression timing and previously reported causal interactions [1-3,7,13,19,22,29,31-33] between genes related to the Wnt, Notch and Fgf pathways. The timing, statistical error and directions of causation are presented in Figure 3. Where directionality could be inferred from data, we found approximately 87% agreement (Figure 3: solid green arcs) between timing and previously reported causation, while only 13% are in the opposite order (Figure 3: solid red arcs). Approximately 14% of the gene interactions involved genes in opposite phases, defined by separation of over 45 minutes, for which causation could not be inferred (red dotted arcs in Figure 3).

The timing and directionality network establishes the hierarchy between Wnt, Notch and Fgf signaling. The data indicate that Wnt signaling is acting upstream of Notch signaling. This Wnt-Notch directionality rule holds strictly for any regulation that may exist. This result confirm previous experiments showing that Notch signaling oscillations depend on the intact and appropriate Wnt signaling and suggest that the Wnt signaling may be central to the segmentation clock mechanism [33]. Although it is clear that Notch and Fgf signaling oscillate nearly in phase, the hierarchy between those pathways has remained an open question. Our results strongly suggest that Notch signaling is acting upstream of Fgf. We also found that the Fgf inhibitor, *Raf1*, is a bimodal gene with expression peaks at approximately *23 min* and *91 min* of the somite cycle suggesting also an Fgf signaling activity just at the beginning of the somite cycle. Assuming that the secondary peak of *Raf1* is responsible for the *Raf1-Dusp6* interaction, the coincidence between causation and timing order will grow from 87% to 92%. Also, the timing of *Egr1* has a relatively large uncertainty, and if we disregard its regulation by *Raf1*, the rate of agreement between timing and previously reported causation will reach 96%. In this analysis, we used all pairs of genes, with peaks separated by any interval smaller than 45 minutes. It can be argued, that causation in an exclusively transcriptional network requires a minimum time delay between two peaks of expression to of at least 10 – 20 minutes. Taking this into account, and restricting the analysis to 12 min, which is reasonably sufficient for transcription and regulation, the rate of agreement is even higher and reaches 95%. Note, however, that a direct transcriptional regulation between two genes is not required for just-in-time expression. It is possible, that both genes are regulated by a third process that has been evolutionary optimized to express them in a temporal sequence with a shorter interval.

For validation purposes, we analyzed the relation between causation and expression times according to the mouse-2 dataset. A comparison is represented in the Additional file 3: Figure S2 between the expression profiles from the data of Dequeant et al. (referred to as “mouse-1” in the picture) and mouse-2, using *Hes1*, *Dkk1*, *Axin2* as examples of genes with one peak of expression and *Arfrp1*, *Cnn3*, *Tmem30* as examples of genes with two peaks of expression. More generally, the rate of agreement between relative timing of causally connected genes in mouse-1 and mouse-2 is 95%. The agreement between mouse-2 and previously reported causation is 89%.

### The early activation of Ctnnb1 and the modulation of Fgf8

β-catenin *(Ctnnb1)* is a transcriptional activator that regulates embryonic development as part of the Wnt pathway and also plays a major role in the activation of genes in this pathway. It is also known that Wnt is upstream of all signaling pathways known to oscillate in the mouse PSM [19]. In the cytoplasm, β-catenin is an essential component of the Wnt signaling pathway and is required for its function [34]. Wnt activation in cells results in stabilization of cytoplasmic beta-catenin, forming a feedback loop [15,34]. β-catenin also serves an important function in the nucleus. The nuclear beta-catenin interacts with TCF/LEF proteins forming a transcription factor, which in turn activates the expression of the Wnt genes [35,36], which is consistent with the peak of β-catenin preceding the genes from the Wnt pathway with a temporal delay allowing for the transcription and translation of these genes. Aulehla et al. [37] have also shown that Wnt-signaling is dependent on a nuclear β-catenin protein gradient in the posterior PSM.

Those observations are all verified in our model in terms of causation as *ctnnb1 (~5 min)* is activated early in the process and according to the timing, it activates successively *Myc (~20 min), Axin2 (~20 min), Dkk1 (~22 min), Sp5 (~23 min), Has2 (~24 min), Tnfrsf19 (~20 min), Phlda1 (~30 min)*, and *Dact1 (~31 min).* It has been shown [37] that somitogenesis is not disrupted by constitutive stabilization of β-catenin in an embryo. This observation does not however conflict with the observed peak of expression. Most likely, while the presence of β-catenin is essential at a specific phase of the somite cycle, its absence in other phases is not required – and the observed pattern is an effect of evolutionary optimization or redundancy built into the system.

For many Fgf genes, both a spatial gradient [38,39] and an oscillatory behavior have been observed. This dual nature is not understood [30]. The overall gradient and its function in creating the determination front suggests that Fgf acts upstream of Wnt and Notch, which also agrees with the cyclic nature of the process. *Fgf8* is a gene from the Fgf family with multiple roles in development, including determination of the anteroposterior body axis, gastrulation, limb development as well as pattering of the face and the midbrain/hindbrain region [40-42]. The results of our analysis show modulation of the *Fgf8* transcript (see profile in Additional file 4: Figure S9), which is surprising, because in the experiments of [43] no production of new *Fgf8* mRNA was observed in the PSM. It is possible that the observed profile of *Fgf8* is a consequence of modulated degradation of *Fgf8* mRNA. Although the modulation may not be strictly transcriptional, it may still play a role in optimization of the somite formation process. Our timing results show an expression peak of *Fgf8* which is later than the peaks of other Fgf genes, and is nearly synchronous with the Wnt pathway. *Fgf8* being active later than other genes in the pathway also agrees with Wahl et al. [30], who show that the expression of *Fgf8* depends of *Fgfr1*, and suggest that *Fgfr1* may directly influence the Wnt and Notch pathways. The relationship between *Fgf8* and other members of the Fgf signaling pathway may be also related to the existence of a second peak for the Fgf signaling inhibitor, *Raf1*.

### Expression of Notch1 precedes Notch-related cyclic genes

The activity of the Notch-related cyclic genes depends on *Notch1,* whose periodic activation can be visualized via the rhythmic production of the Notch Intracellular Domain (NICD). It is believed that after nuclear translocation, NICD activates transcription of target Notch related genes. The process of somite formation was found to be delayed and disorganized in *Notch1* mutant embryos [44], suggesting that this gene should coordinate the process or at least the Notch signaling. Consequently, we should expect activation of *Notch1* before all Notch-related cyclic genes and that is actually the case, as shown in Figure 3.

*Notch1* is expressed approximately *53 min* after the beginning of the somite cycle and then activates successively *Nkd1 (~64 min), Hey1 (~64 min), Nrarp (~65 min), Hes7 (~71 min), Lfng (~76 min), Hes5 (~80 min) Hes1 (~88 min*) and *Bcl9l (~97 min)*. Analysis of the Mouse 2 dataset confirms these findings; Notch1 peaks at 50 min and its targets between 53 and 90 minutes.

### Hes7 displays two peaks of expression

The role of the *Hes7* gene, and its zebrafish homologs *Her1* and *Her7*, has been extensively studied in the recent years. The primary function of *Hes7* in somitogenesis is related to its interaction with the Notch modulator Lfng [45-48], which results in a wave of *Hes7* activity in phase with genes from the Notch pathway. In the analysis of the deconvolved data, we have observed two peaks of expression of *Hes7* (see panel on page 4 of Additional file 4: Figure S9)*.* The first peak at 71 min after the beginning of the somite cycle is consistent with the Notch signaling activity and the Hairy and enhancer of split Family (*Hes1/5/7*), and is exactly in phase with *Lfng*. Niwa et al. [49] have discovered a secondary function of *Hes7*, related to its initiation by the FGF pathway. *Lfng* knockout experiments suggest that these two pathways are largely independent from one another. This function is more consistent with the second peak of expression of *Hes7,* at 13 min after the beginning of the somite cycle. The bimodal expression pattern of *Hes7* is confirmed by the mouse-2 dataset, where the same pattern is observed consisting of two peaks, one following FGF and one in late Notch phase.

### Raf1, an Fgf-related cyclic genes activator is bimodal

The timing of most known cyclic genes during mouse somitogenesis suggests the following relationship between signaling pathways: Wnt acts upstream of Notch, which in turns acts upstream of Fgf. On the other hand, activities of some Fgf signaling have been found to take place at the beginning of the process. Moreover, several results (see [50] for review) have suggested that cells in the posterior-most tissues are maintained undifferentiated by a high level of FGF signaling and activate their differentiation program only when they reach the appropriate threshold of FGF activity. Although those observations can be related to the gradient of *Fgf8* creating the asymmetry along the rostral-caudal axis of the PSM, another explanation may come from the bimodal behavior of *Raf1*. Indeed, *Raf1* (*v-raf-leukemia viral oncogene 1*), which is known to regulate indirectly members of the Fgf signaling pathway, was found to have two peaks of expression. The first expression peak of *Raf1*, which happens *23 min* after the beginning of the somite cycle, can explain the regulation with some genes like *Tbxl6 (~31 min)* and *Fgf8 (~17 min)*, while the second peak, *91 min* after the beginning of the somite, is closer to the known Fgf cyclic genes.

### Other bimodal genes

Analysis of the timing of gene expression during mouse somitogenesis indicates that genes with multiple expression peaks may be involved. Indeed, the dual peaks of expression of genes like *Hes7* and *Raf1* is obvious and may explain their role in the process.

In most studies on somitogenesis, attention is paid to genes with oscillation periods matching that of somite formation. Although this is the most appropriate way to support the idea of the “clock and wavefront” model, studying genes with multiperiodicity or multifrequency can help understand some concepts behind the process. Indeed, a transcript with 60-minutes period is also periodic within a 120 minutes process, but the 60-minutes periodicity will not produce a Fourier peak a 120 minutes and such transcripts are often missed.

Genes oscillating at different harmonic modes have been previously observed in cyclic processes. A prominent example is the cyclin-dependent kinase *CDC28* with two peaks during the mitotic cycle [24]. Examples in development include the recently reported two-segment periodicity in insect segmentation clock [51]: in the beetle *Tribolium casteneum*, a short germ-band insect, the segmentation gene *odd-skipped (Tc-odd)* oscillates with a two-segment periodicity, which shows that a multi-periodicity or half periodicity may occur during segmentation processes.

The bimodality of *Hes7* and *Raf1* suggests that other bimodal genes could be involved in the mouse somitogenesis. We used the previous LS algorithm with double frequency to find those genes whose period is half of that of somite formation. We detected 247 probe sets with a bimodal signal by setting the p-value, statistical significance level of testing the null hypothesis that a double peak in LS periodogram is due to chance, at 0.05. After deconvolution, peak detection, error estimation and visual check of individual profiles, we ranked the genes according to the regularity of their profiles. The complete set of genes (173) found to be statistically significant with a regularly smooth profile is presented in the Additional file 5: Table S2 and the supporting website at http://moment.utmb.edu/somites. This set contains genes previously associated with Wnt (*Ccnd3, Csnk2a1*), Notch (*Cbln1, Csnk2a1*) and Fgf (*Pik3ca, Fgf13, Mapk14*) pathways. The positions of peaks of the 173 bimodal genes along the somite cycle are shown in Additional file 6: Figure S3. A majority of these genes have one peak of expression in the late Wnt phase and the other one in the late Notch/Fgf phase.

Table 2 contains the 20 most significant bimodal genes; their expression profile and timing are shown in Additional file 7: Figure S4 and Additional file 8: Figure S5 respectively. To our knowledge, none of these genes have been previously reported to have any critical function during mouse somitogenesis. *Arfrp1*, the gene with the most regular bimodal profile (see Figure 2 and panels *a* and *c* of Additional file 3: Figure S2), has been reported to play a major role in such processes during early gastrulation as adhesion-dependent morphogenesis, cytoskeletal reorganization, and/or development of cell polarity and its deletion in mice results in embryonic lethality [52]. It should be noted that the bimodality is confirmed by the deconvolved data of [13] as seen in Additional file 3: Figure S2. We expect that the discovered bimodal profiles will lead to constructing more accurate models of somitogenesis and to finding new functions of the genes involved.

**Table 2:** Timing of the most notable cyclic genes with two peaks of expression per cycle

**Probeset**	**Gene**	**T**_**1**_**(min)**	**T**_**2**_**(min)**	**LS p-value**
1425508_s_at	*Arfrp1*	37 ± 4	96 ± 3	0.00231
1417405_at	*Stard3*	28 ± 3	91 ± 4	0.00261
1416446_at	*Tmem30a*	37 ± 3	101 ± 2	0.00286
1426017_a_at	*0610011L14Rik*	31 ± 2	95 ± 4	0.00387
1426359_at	*Zc3h11a*	28 ± 2	86 ± 5	0.00392
1456380_x_at	*Cnn3*	29 ± 1	84 ± 3	0.00555
1417108_at	*Klc4*	34 ± 3	86 ± 8	0.00695
1448478_at	*Med20*	29 ± 2	89 ± 1	0.00717
1426524_at	*Gnpda2*	28 ± 1	83 ± 3	0.00748
1450953_at	*Ciao1*	32 ± 3	89 ± 4	0.00798
1448155_at	*Pdcd6ip*	41 ± 3	96 ± 3	0.00799
1418017_at	*Pum2*	35 ± 2	103 ± 1	0.00808
1452053_a_at	*Tmem33*	45 ± 3	100 ± 5	0.00823
1451243_at	*Rnpep*	31 ± 3	92 ± 2	0.00828
1448762_at	*Rad17*	37 ± 4	98 ± 4	0.00872
1427356_at	*Fam89a*	37 ± 3	100 ± 2	0.00883
1423286_at	*Cbln1*	17 ± 1	77 ± 5	0.00942
1460718_s_at	*Mtch1*	29 ± 1	87 ± 9	0.00947
1452560_a_at	*Nfya*	24 ± 2	88 ± 5	0.00985
1448389_at	*Wdr5*	89 ± 3	28 ± 3	0.0101

Times in minutes are provided for both peaks (T1 for the first and T2 for the second) and scaled to a 120-minute somite cycle.

## Conclusions

We have developed a variant of the maximum entropy deconvolution formalism that can describe spatiotemporally variable processes. Our algorithm, supplemented with a customized method for data preprocessing, allowed the successful reconstruction of transcriptional events during mouse somitogenesis with a high accuracy and an unprecedented temporal resolution. The results demonstrate that the genes involved in the process are transcribed precisely when their products are needed, and that the timeline of gene expression agrees with the direction of causation in the regulatory network of somitogenesis. This strongly suggests that the temporal structure of the segmentation process is fully reflected by the timeline of transcriptional activity. The agreement holds even for genes with demonstrated posttranscriptional or posttranslational modifications, (e.g. beta-catenin). A plausible explanation of such highly prevalent just-in-time expression is through the evolutionary pressure towards economizing on resources in the living cells – in predictable temporal processes a cell will conserve energy and amino acid components if a gene product is made just before it is needed, as opposed to long-time storage and maintenance of proteins. The deconvolution acting as a noise filter has revealed prominent peaks in the temporal profiles of many genes previously not annotated as cyclic (as beta-catenin), and for a number of transcripts two phases of activity have been identified per somite cycle (including *Raf1* and *Hes7*). Our findings are confirmed by applying the algorithms to a second independent dataset. Although some differences exist between these two datasets, the ordering of causally-related genes is almost universally conserved.

The timeline of expression peaks will serve as a benchmark for newly identified causal interactions in somitogenesis, as well as a tool to generate and test further hypotheses concerning the regulatory network involved. Our results demonstrate the utility of high-resolution timing of gene expression in deciphering the regulations in transcriptional networks in general.

The statistical and computational methods developed in this work are readily applicable to interpret the results of further gene expression studies of somitogenesis in mouse and other species as well as to other developmental processes.

## Methods

### Positioning of embryos along the clock cycle

In the somitogenesis study each data point comes from a different embryo and no accurate wall-clock is present, so information about the phase of the somitogenesis cycle may be derived only from the embryo itself. Dequeant et al. have derived the order of embryos in the cycle using *in situ* hybridization of Lunatic fringe glycosyltransferase (*Lfng*) in the contralateral half embryo (see Figure 1) for the 17 data points; this dataset will be referred to as “mouse-1”. In the study by Krol et al. [13], a similar procedure, using Affymetrix GeneChip Mouse Genome 430 2.0; an updated version of the microarray platform, has produced a sequence of 20 data points - referred to as “mouse-2”. We use the data of Dequeant et al. as the primary set. The expression profiles from Krol et al. are used for validation of the inferred timeline of gene activation during somitogenesis.

To estimate the actual positions of embryos along the 2 h clock cycle, we used the set of 6 known cyclic genes (*Hes1, Hes5, Hey1, Lfng, Axin2 and Nkd1*) from Dequeant et al. [2]. The aim is to assign times to measurements such that the expression profiles of these 6 genes are the most periodic, as measured by the amplitude of the best-fit harmonic wave. Specifically, we start from an evenly spaced time distribution, with approximately 7 and 7.5 minute interval between two consecutive points in the mouse-1 and mouse-2 dataset respectively. We vary the time *t*_*i*_ associated with each embryo by adding a small value *ϵ*_*i*_ to *t*_*i*_ in order to maximize the Lomb-Scargle (LS) [53] periodicity scores of the six cyclic genes.

The LS periodogram is a common tool in the frequency analysis of unequally spaced data. Let (*t*_*i*_, *x*_*i*_), *i* = 1…*n*, been the time point distribution representing for example the expression values *x*_*i*_ of a gene *g* at different time *t*_*i*_, then the LS periodogram for a single frequency ω is defined by

(1) $$\begin{array}{l} P_{g}\left(\omega \right)=\frac{1}{2\sigma^{2}}\left[A{\left(\sum_{j=1}^{n}\left(x_{j}-\bar{x}\right)\cos\left(\omega t_{j}-\tau \right)\right)}^{2}\right)\\ \;\left(+B{\left(\sum_{j=1}^{n}\left(x_{j}-\bar{x}\right)\sin\left(\omega t_{j}-\tau \right)\right)}^{2}\right] \end{array}$$

Where $A=1/\sqrt{\sum_{j=1}^{n}\left(x_{j}-\bar{x}\right)\cos{\left(\omega t_{j}-\tau \right)}^{2}}$, $B=1/\sqrt{\sum_{j=1}^{n}\left(x_{j}-\bar{x}\right)\sin{\left(\omega t_{j}-\tau \right)}^{2}}$, *σ*^*2*^ is the sample variance, $\bar{x}$ is the mean of *x* , τ is defined by $\tan\left(2\omega \tau \right)=\sum_{j=1}^{n}\sin\left(2\omega t_{j}\right)/\sum_{j=1}^{n}\cos\left(2\omega t_{j}\right)$ and *n,* the total number of time points is 17 for mouse-1 and 16 for mouse-2.

The null distribution of the LS periodogram *z*_*g*_ = *P*_*g*_(*ω*) at frequency ω is computed using the Fisher rule *F*(*z*_*g*_) = (1 − *z*_*g*_/*n*)^*n*−1^[54]. A periodic gene expression is obtained by testing the null hypothesis, *p*_*g*_ = 1 − (1 − *z*_*g*_/*n*)^*n*−1^ , that the gene is non-periodic versus the alternative that it is periodic. For the set of known cycling genes described above, their combined periodicity significance (equation 2) is minimized with an additional given penalty λ for large deviations. Combining the periodogram with the penalty results in the following target function *T*:

(2) $$T\left(\epsilon_{1}\ldots \epsilon_{n}\right)=\sum_{g=1}^{6}\left(\log\left(1-{\left(1-z_{g}/n\right)}^{n-1}\right)\right)+\lambda \sum_{j=1}^{n}\epsilon_{j}^{2}$$

**Figure 4:**
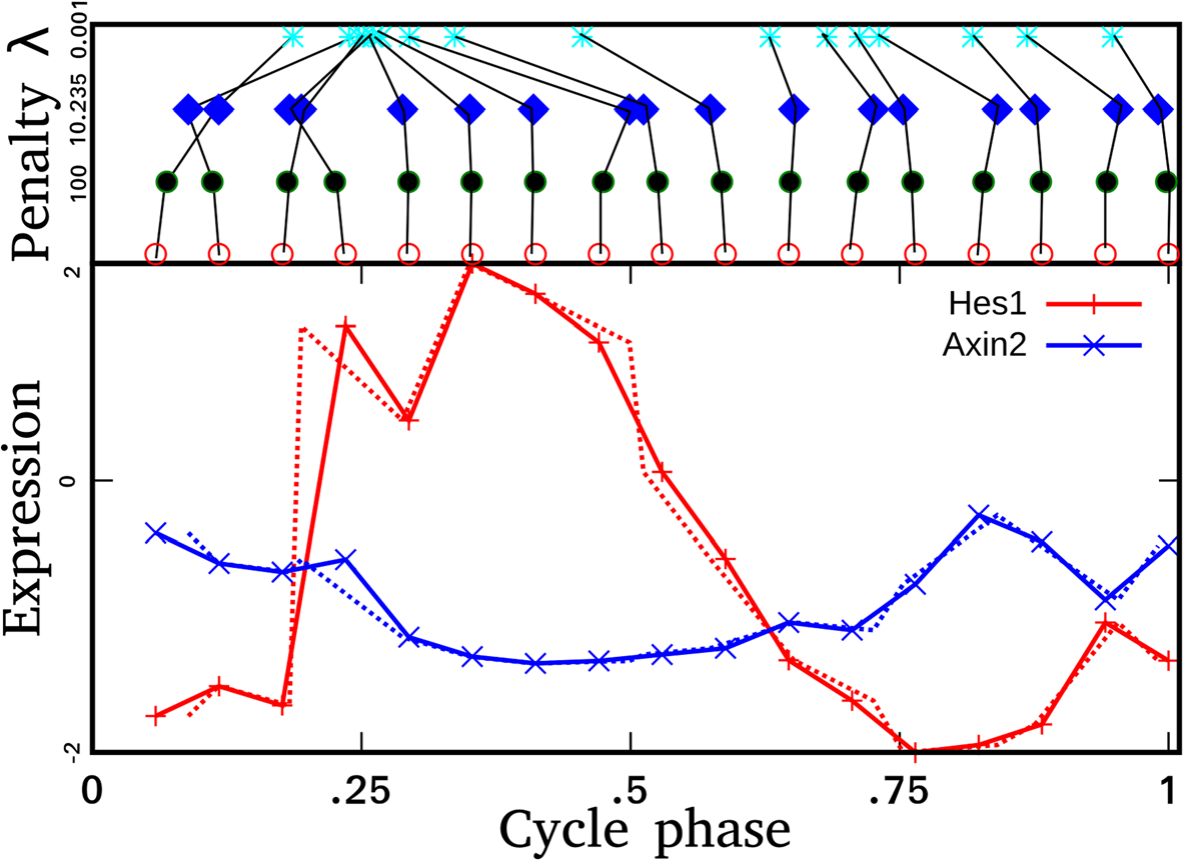
**Optimization of positioning of experiments along the somite cycle.** Bottom panel: The temporal profiles of *Hes1* and *Axin2* plotted against original (solid lines) and optimized time points (dotted lines). Top panel: The result depends on the choice of the amount of allowed variation during optimization of timepoints (see Methods). The allowed variation depends on the value of weight penalty λ in Eq. 2. Higher values of λ will overemphasize the regulation leading to the reconstruction of the original points while too small values will overweight the goodness-of-fit component leading to a poor regulation. The value 10.2 (in the mouse-1 dataset) was found to be a good consensus, giving high periodicity scores without sacrificing the global properties of the profiles.

The Powell optimization method [27] is applied to the target function to optimize the values of *ϵ*_*i*_. Figure 4 gives an illustration how the amplitude of λ may affect the final result: High values of λ will overemphasize the regulation leading to the reconstruction of the original points while too small values will overweight the goodness-of-fit component leading to a poor regulation. We found that for the mouse-1 dataset, λ =10.2 results in adequate balance between the periodicity and penalty components. For mouse-2, we used λ =23.7; the difference can be attributed to different noise levels and different numbers of data points in the two datasets.

### Spatiotemporal maximum entropy deconvolution

**Figure 5:**
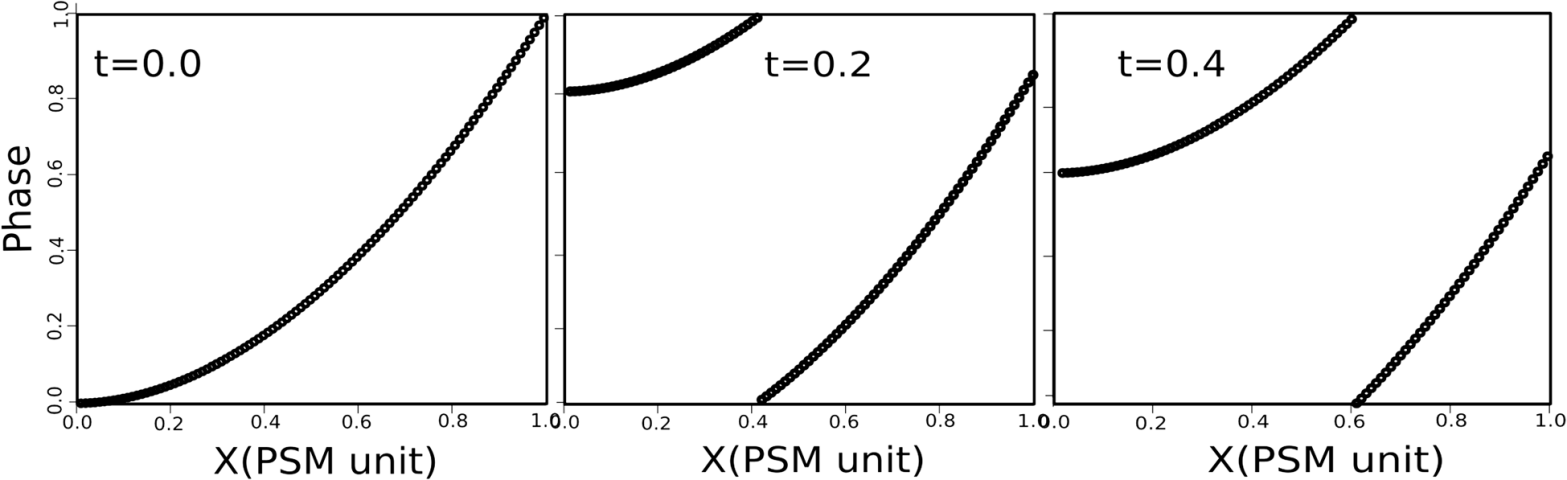
**The relation between the phase of gene expression and position along the PSM.** The waves start as moving fast for small values of *x* (most posterior part of the PSM), and slow down as the wave progresses toward larger values of *x*. The three snapshots depict a sequence of time-points, corresponding to different phases of the somite cycle.

Because of relatively large spatial dimensions of the sample, it will contain cells in different stages of the cycle, which will affect on the observed temporal changes in gene expression. To correct for this effect, we first model the dependence between the position of the *Lfng* band *x*, the time *t* and phase *φ* as

(3) $$\phi \left(x,t\right)={\left(\frac{x}{1-d}\right)}^{\alpha }-t$$

where the parameters *α* and *d* describe the wave deceleration and geometry of the system. The cycle phases *φ*_*i*_ are random when the embryos are sacrificed. Therefore, a correct transformation between *x* and *φ* is expected to yield cycle phases consistent with a random, flat distribution. To ensure this, we require that the moments of the actual distribution of timepoints will be the same as in a random distribution. To this end, we choose such values of *α* and *d*, for which the first two moments of the distribution of the phases *φ* are the same as the moments of a flat distribution: < *ϕ* > = 1/2 for the first moment and < (*ϕ* − 1/2)^2^ > = 1/12 for the second moment. These two constraints uniquely determine the values of the geometry parameters as *d = 0.022* (PSM unit length) and *α* = *1.900* when *t = 0.0*. Figure 5 represents the phase of gene expression along the PSM: The waves are moving faster for small values of x (most posterior part of the PSM), and slow down for larger values of *x*. The values of *x* used in this calculation are obtained by analyzing the in-situ micrographs of [2] to identify the position of maximum intensity of the *Lfng* band.

The observed spatiotemporal patterns ***M*** of gene expression in the PSM result from convolution of the primary oscillation ***E*** with a geometry-dependent kernel function ***h*** (Figure 1). In the phase space, the observed spatiotemporal pattern is given by:

(4) $$M\left(\phi \right)=\int E\left(\phi \right)h\left(\phi \right)\mathrm{d\phi}$$

Experimentally, the samples are collected from the posterior half right side of the PSM, approximately between positions *x*_*p*_ *= 0.05* and *x*_*a*_ *= 0.5* (PSM length unit), and the measurements *M* correspond to integration over this interval. For a time point *t*_*i*_*,* the observed spatiotemporal pattern *M(t*_*i*_*)* is given by

(5) $$M\left(t_{i}\right)=\int_{x_{p}}^{x_{a}}E\left(x,t_{i}\right)\mathrm{dx}=\int_{\phi_{p}^{i}}^{\phi_{a}^{i}}E\left(\phi \left(x,t_{i}\right)\right)\frac{\mathrm{dx}}{\mathrm{d\phi}}\mathrm{d\phi}$$

Here $\frac{\mathrm{dx}}{\mathrm{d\phi}}=\frac{1-d}{\alpha }{\left(\phi +t\right)}^{\frac{1}{\alpha }-1}$

The convolution kernel as function of phase is given by: $h\left(\phi ,t\right)=\left(\left(1-d\right)/\alpha \right){\left(\phi +t\right)}^{\frac{1}{\alpha }-1}\;\mathrm{for}\;\phi_{p}^{i}<\phi <\phi_{a}^{i}\;\mathrm{and}\;0\mathrm{elsewhere}.$ The temporal gene expression profile, *E φ(x,t*_*i*_*))*, is represented by values of mRNA concentrations, *a*_*j*,_ at 100 points evenly spaced in time: *a*_*j*_ *= E*(*φ = j/*100), for *j =* 1..100. The expression value at *φ =* 0 is represented by *a*_*100*_. The likelihood of observing a particular temporal profile *M* from a given true profile *E* at the tail of the embryo (which is also characteristic of any typical single-cell in the PSM) is based on the least squares principle: *prob*(*M | E*) ≈ *exp*(−*x*^2^/2), where

$$\chi^{2}=\sum_{i=1}^{n}{\left\{M\left(t_{i}\right)-\sum_{j=1}^{100}a_{j}h\left(\phi_{j},t_{i}\right)\right\}}^{2}$$

We employ a Bayesian approach and to calculate the probability of a profile, we combine this likelihood with a prior probability for the original profile *E*, which is chosen based on the maximum entropy principle.

The maximum entropy prior is chosen as one correctly describing a distribution of a variable that is non-negative and additive, and for which no additional prior information (prior to experimental data) is available [55]. The use of maximum entropy prior is justified even if periodicity of a profile is presumed, because periodic processes are possible (and observed in biology) with any shape and distribution, not necessarily sinusoidal. The most probable solution for the profile – represented by the parameters {*a*_*j*_} is the one maximizing *prob*(*M | E*) while keeping the posterior probability normalized to 1. Under the maximum entropy prior, the final target function has the form $T\left(a_{1},\ldots ,a_{100}\right)=\frac{\omega }{A}\sum_{j=1}^{100}a_{j}\log\left(a_{i}/A\right)+\frac{1}{2}\chi^{2}$,

where $A=\sum_{j=1}^{100}a_{j}$. We find the optimal values of *a*_*j*_ by minimizing *T* using conjugate gradient method.

The result depends on the choice of *ω*, which can be viewed as Lagrange multiplier. The parameter *ω* measures regularization introduced into the least square minimization through the entropy condition. There is no consensus for the choice of *ω*[56]. Too small a value of *ω* will over-weight the goodness-of-fit component, thus favoring high-frequency solutions, which are poorly regularized. On the other hand, too high a value of *ω* will overemphasize the entropy regularization, which will result in under-reconstruction of the high-frequency components of the profile (artificially flat profile). The optimal value of the entropy weight *ω* is established based on numerical experiments.

### Microarray data and periodicity detection

As primary set, we used the data of [2], retrieved from the ArrayExpress database under accession E-TABM-163. The 17 time points correspond to embryos harvested approximately 9 days post-coitus (ranging between 19 and 23 somites), and *mRNA* concentrations in the right posterior half of the PSM are measured using the Affymetrix GeneChip M0E430A microarray. Following [2], we only used probe sets with maximum signal over than 80 and a peak-to-trough ratio of 1.7 or higher (5822 probe sets). Fourier amplitudes were computed as in [53], 276 probesets passed the p-value threshold of 0.05 [54]. We individually inspected the regularity of each temporal profile. The resulting set contains genes with a regular peak suitable for timing with a good resolution and includes about 95% of previously known cyclic genes. Indeed, from the set of 22 most cyclic genes proposed by Dequeant et al. in [2], only 2 genes (*Shp2* and *Nkd1*) are not present in the remaining set, due to their low signals.

The dataset of [13], which was used for independently validating our results has been retrieved from the ArrayExpress database under accession E-MTAB-406. This collection of profiles set (referred to as mouse-2) contains 20 mouse embryos analyzed using the same protocol as in Dequeant et al. Only 16 out of 20 embryos were selected by the authors to cover the 2 h clock cycle. The data were filtered by the percentage of p-calls (only probesets called present by the detection call for at least 2/3 of the samples of the microarray series were retained), minimum signal intensity (probesets with minimum signal less than 29 were removed) and peak-to-trough (only probesets with a ratio of 1.5 were selected). This filtering reduced the dataset by 43% and for the remaining 194318 probesets, we have repeated the procedures described in the sections above to extract the individual profiles, estimate the timing as well as its resolution. In the affymetrix platform, several probesets may be available for one gene. Whenever such probesets displayed diverging expression profiles, we matched the probesets between the MOE430A and 430–2.0 – based experiments based on profile similarity. Moreover, *Nkd1*, *Shp2*, *Lfng* despite their low signals or low p-calls were included in the results as they are known to be involved in mouse somitogenesis. The position of peaks for those genes along the 2 h somite cycle is shown in Additional file 9: Figure S6.

The list of genes involved in the mouse somitogenesis has been derived and refined in several studies using a number of approaches [1,2,13,28,31,57-60], however the poor S/N ratio may be the source of both false positive and false negative detections. As in [24,61], we follow the observation that truly regulated transcripts will exhibit an observed expression profile that is not only periodic but also has the characteristics of a convolved signal. Using this additional information, we postulate the presence of previously undetected genes that are transcriptionally regulated during somitogenesis. The full list of genes Additional file 5: Table S2 for genes with two peaks of expression and Additional file 10: Table S3 for genes with one peak of expression is shown in the supplementary material and can also be downloaded from the supporting website. Each list contains the probe set ID, the gene symbol, the timing, the precision and the LS p-value. The expression profiles of those genes are also shown in the Additional file 11: Figure S7 (genes with two peaks of expression) and Additional file 12: Figure S8 (genes with one peak of expression). Additional file 4: Figure S9 represents the expression profiles of common genes discussed in the text. Additionally, we provide an online tool to plot and/or download the profile and timing for each gene, available at http://moment.utmb.edu/somites.

### Peak detection and stability

To find the peaks of the profiles, we search for maxima of the deconvolved expression profile. of course, the deconvolved profiles can have multiple local maxima, only some of which correspond to actual significant peaks in gene expression. To identify significant peaks, we design a heuristic score, taking into account the integral under the peak and the peak's full width at half magnitude. To estimate the accuracy of the timing for each individual gene, we have created a large number of synthetic datasets (100 artificial profiles for every gene) by adding to the actual data, sources of noise typically found in microarray experiments, the amplitude of noise set to 0.4 of the expression level. For each gene, the simulated profiles were deconvolved and peaks identified. The accuracy (resolution) of the gene timing was computed the root mean square deviation of timing results (peak position) of the synthetic profiles.

### Supporting website

The presented data are also available from the supporting website.

http://moment.utmb.edu/somites.

## Competing interests

The authors declare that they have no competing interests.

## Authors’ contributions

BF and AK conceived of the study, developed methods, analyzed and interpreted data and drafted the manuscript. Both authors read and approved the final manuscript.

## Supplementary Material

Additional file 1: Figure S1 Profiles of the main genes used in the text and known as Wnt, Notch or Fgf cyclic genes for mouse somitogenesis. The figure presents the individual profiles the well-known cyclic genes compiled as well new candidate cyclic genes. Click here for file

Additional file 2: Table S1 Timing of genes known to be associated with Fgf, Wnt and Notch pathways in the data set mouse2. Click here for file

Additional file 3: Figure S2 Expression profiles are conserved between mouse-1 and mouse-2 datasets. In the figure, we compare the expression profiles between mouse-1 (Dequeant et al.) and mouse-2 (Krol et al.) using *Hes1*, *Dkk1*, *Axin2* as examples of genes with one peak of expression (b,d) and *Arfrp1*,*Tmem30a*, *Cnn3* as example of genes with two peaks of expression (a,c). Click here for file

Additional file 4: Figure S9 Expression profiles for genes discussed in the text. The figure represents the expression profiles of common genes discussed in the text. Click here for file

Additional file 5: Table S2 The list of genes with two peaks of expression. The timing of genes found with two peaks of expression, ranked according to their LS p-value and the regularity of the profile. Times in minutes assume a 2 h periodicity for every transcript and errors are computed by adding to the original transcript source of noise typically found in microarray experiments. Click here for file

Additional file 6: Figure S3 Timing of genes with two peaks of expression. The positions of peaks of 173 bimodal genes along the 2 h somite cycle are shown. Most of those genes have one peak of expression in the Wnt phase and the other one in the Notch/Fgf phase. Click here for file

Additional file 7: Figure S4 Profiles of the top 20 genes with two peaks of expression. Click here for file

Additional file 8: Figure S5 Timing of the top 20 genes with two peaks of expression. Click here for file

Additional file 9: Figure S6 Timing of genes with one peak of expression. The positions of peaks of 159 genes along the 2 h somite cycle are shown. Click here for file

Additional file 10: Table S3 The list of genes with one peak of expression. The timing of genes found with one peak of expression, ranked according to their LS p-value and the regularity of their profile. Times in minutes assume a 2 h periodicity for every transcript and errors are computed by adding to the original transcript source of noise typically found in microarray experiments (see text). Click here for file

Additional file 11: Figure S7 Individual profile for every gene listed in the Additional file 5: Table S2. For every gene in the Additional file 5: Table S2, the deconvolved expression is displayed. Click here for file

Additional file 12: Figure S8 Individual profile for every gene listed in the Additional file 2: Table S1. For every gene in the Additional file 2: Table S1, the deconvolved expression is displayed. Click here for file
