# Supplementary figures and images for: The precise timeline of transcriptional regulation reveals causation in mouse somitogenesis network
Source: BMC Dev Biol. 2013 Dec 5;13:42. doi: 10.1186/1471-213X-13-42 (PMC4235037; doi:10.1186/1471-213X-13-42)

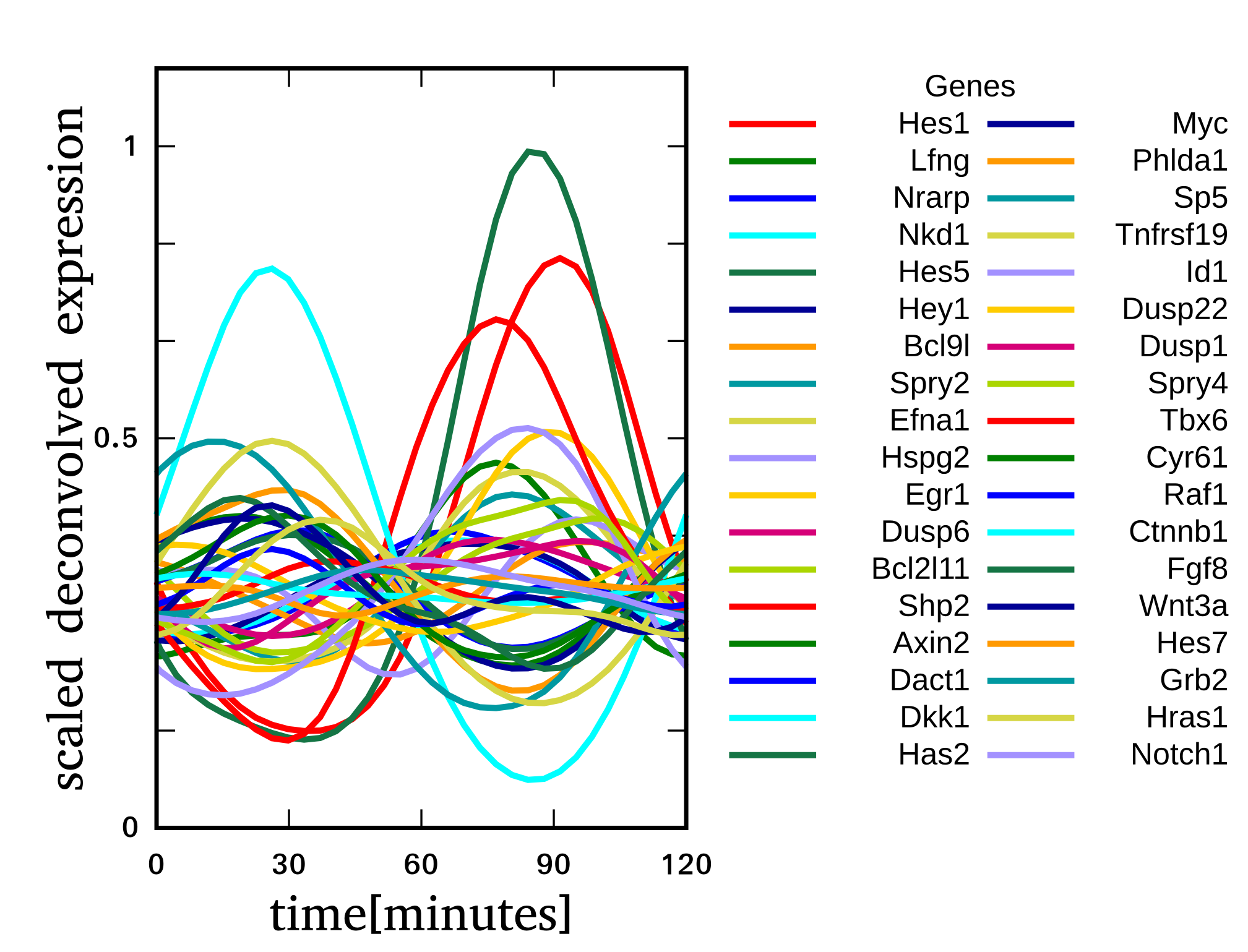

Supplement: Additional file 1: Figure S1 — Profiles of the main genes used in the text and known as Wnt, Notch or Fgf cyclic genes for mouse somitogenesis. The figure presents the individual profiles the well-known cyclic genes compiled as well new candidate cyclic genes. [file 1471-213X-13-42-S1.tiff]

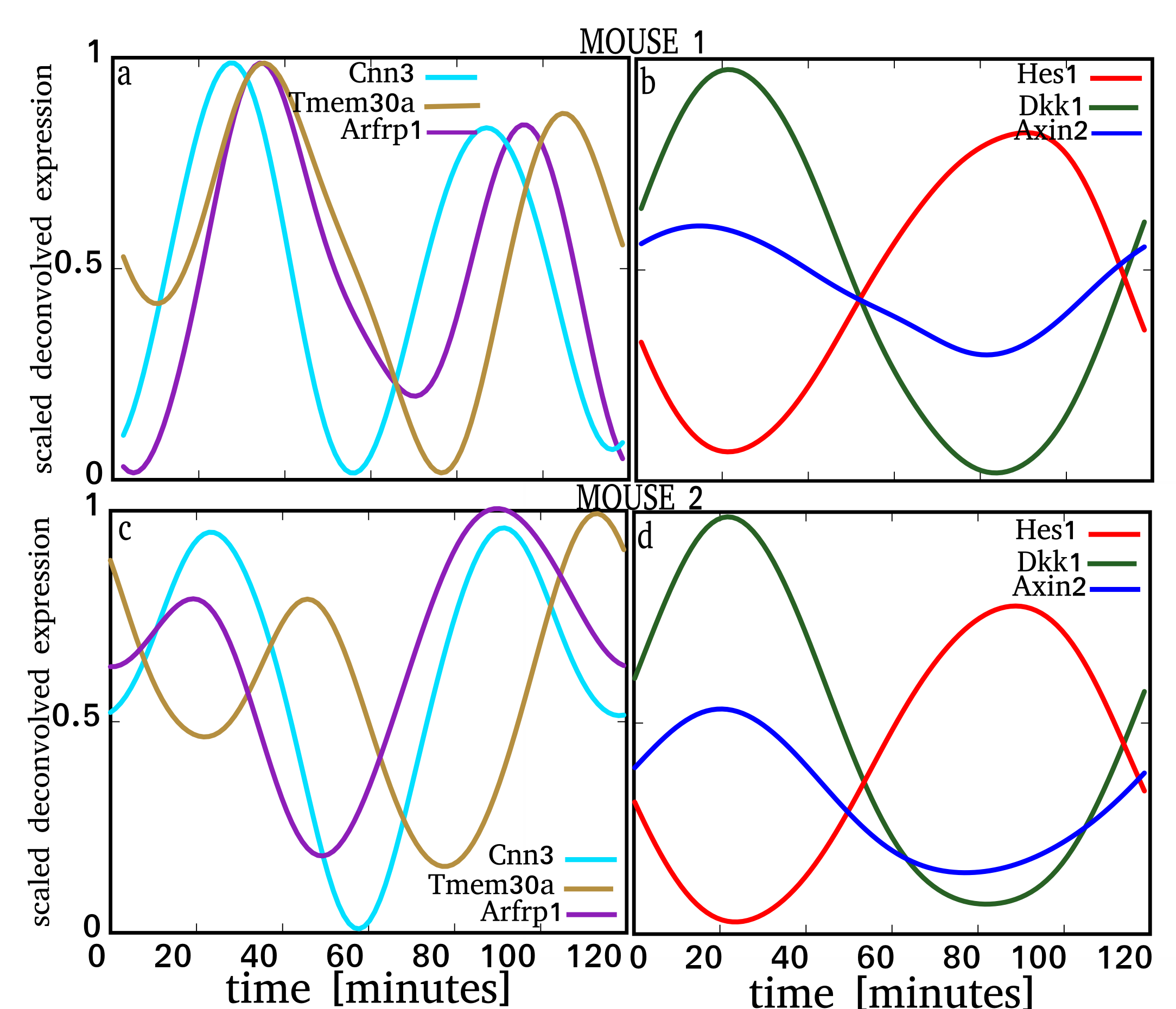

Supplement: Additional file 3: Figure S2 — Expression profiles are conserved between mouse-1 and mouse-2 datasets. In the figure, we compare the expression profiles between mouse-1 (Dequeant et al.) and mouse-2 (Krol et al.) using Hes1, Dkk1, Axin2 as examples of genes with one peak of expression (b,d) and Arfrp1,Tmem30a, Cnn3 as example of genes with two peaks of expression (a,c). [file 1471-213X-13-42-S3.tiff]

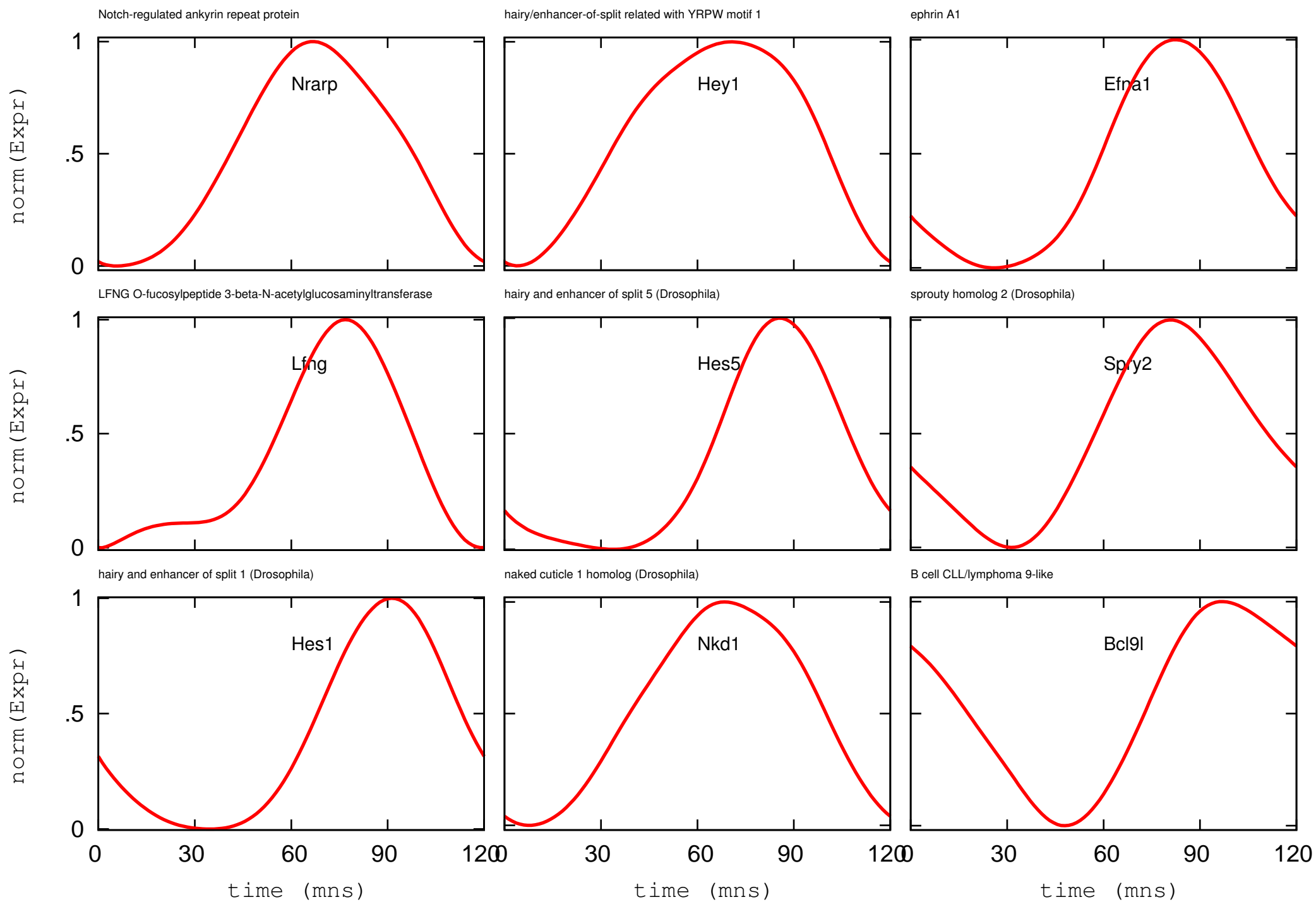

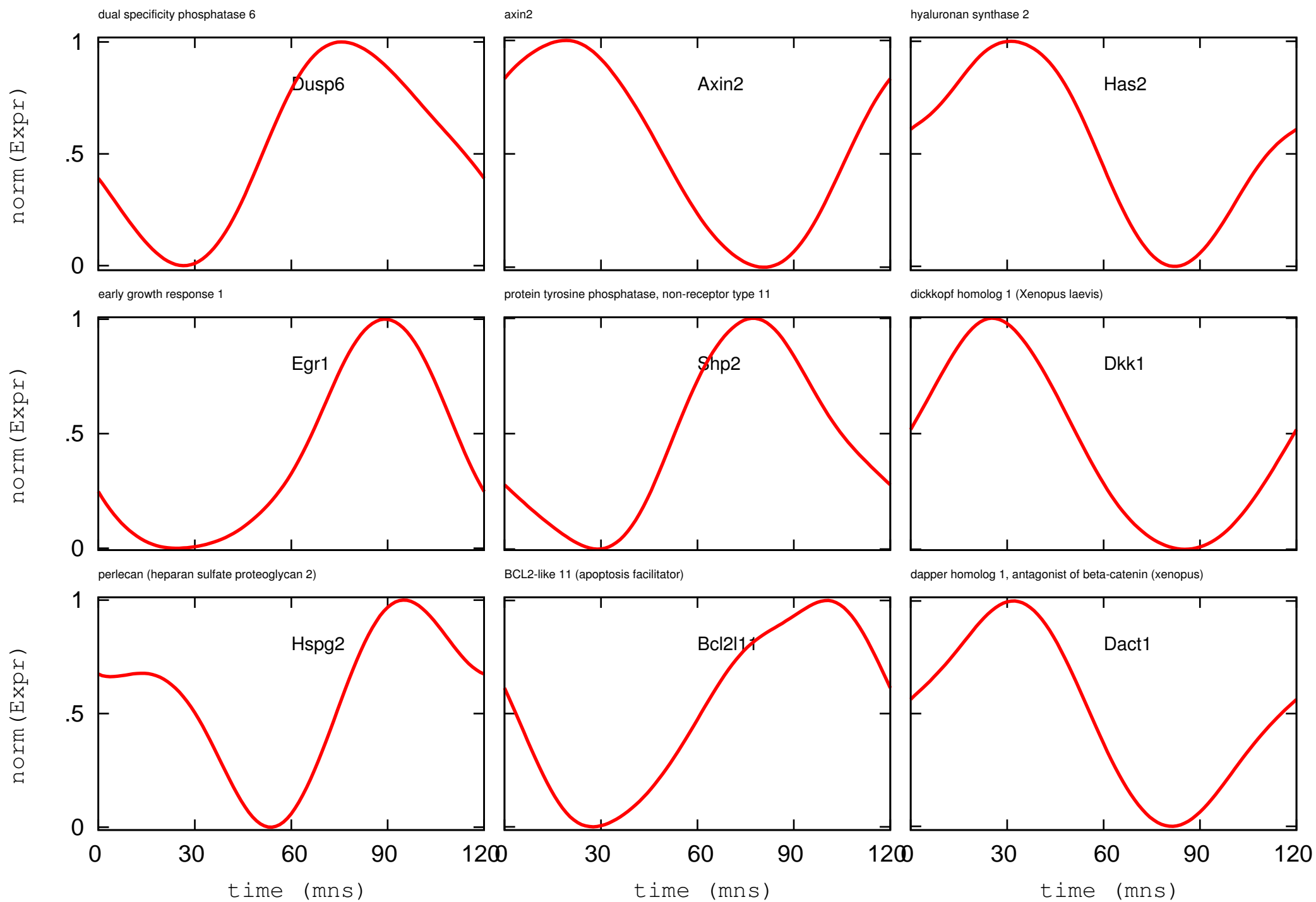

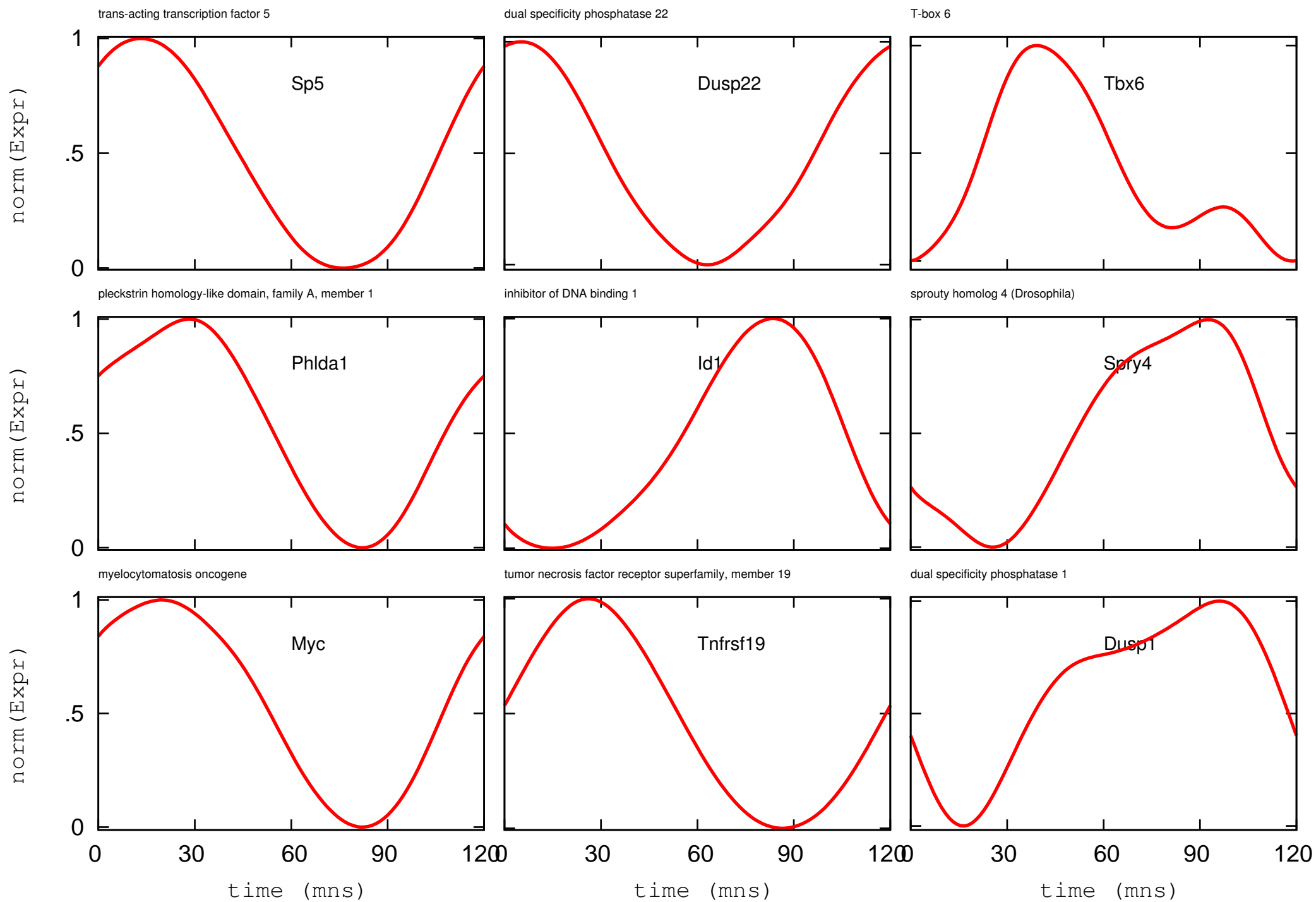

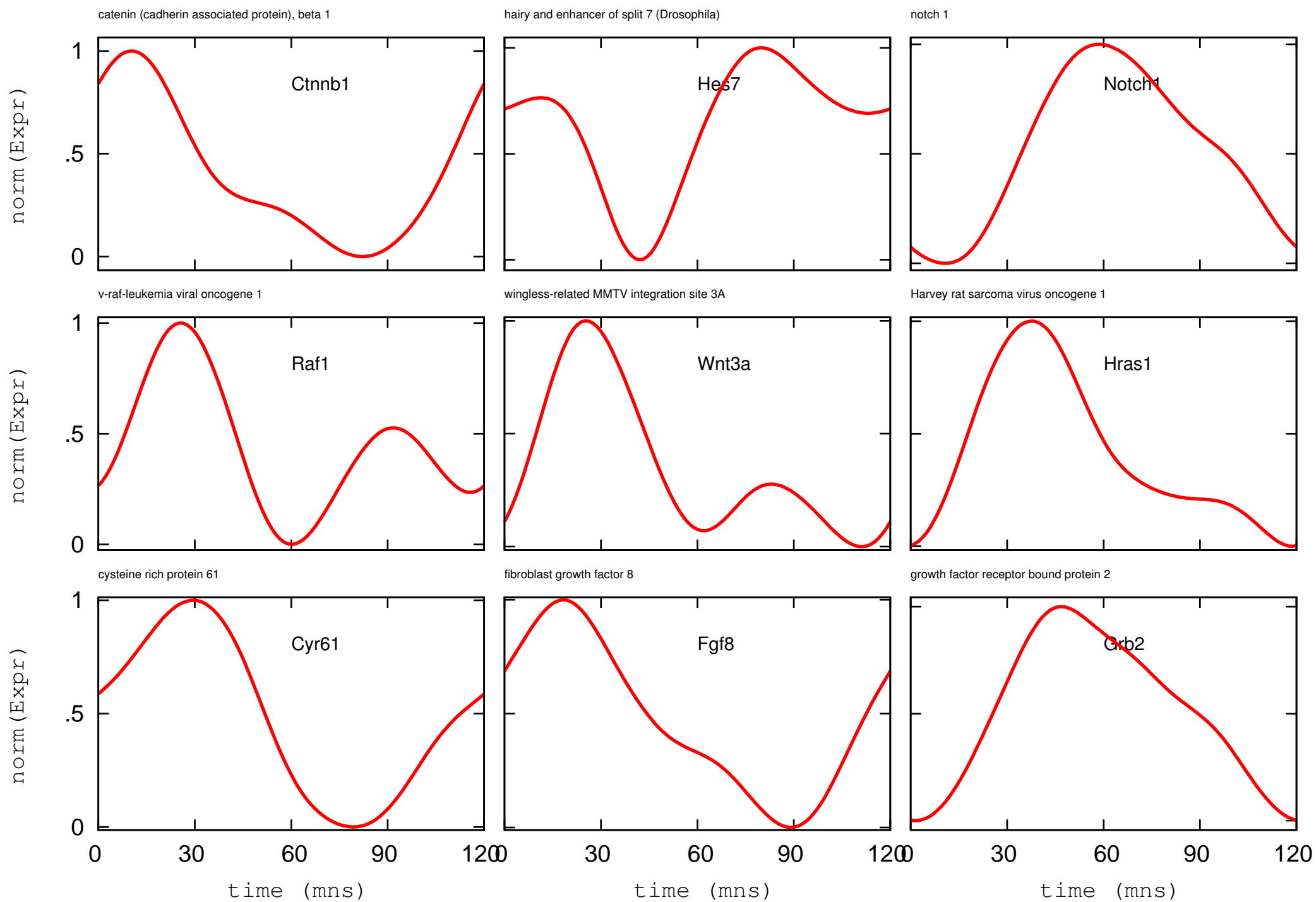

Supplement: Additional file 4: Figure S9 — Expression profiles for genes discussed in the text. The figure represents the expression profiles of common genes discussed in the text. [file 1471-213X-13-42-S4.pdf]

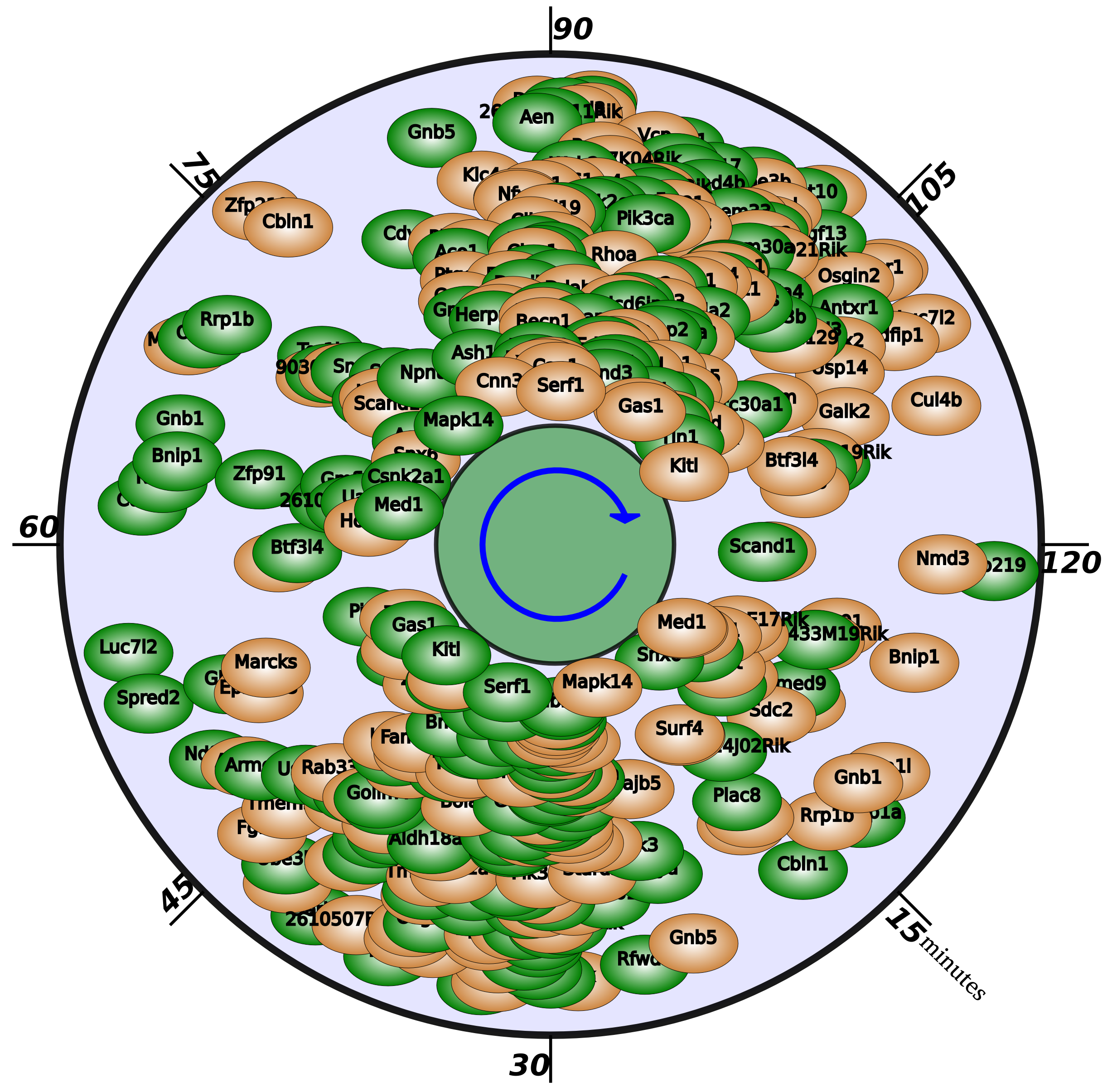

Supplement: Additional file 6: Figure S3 — Timing of genes with two peaks of expression. The positions of peaks of 173 bimodal genes along the 2 h somite cycle are shown. Most of those genes have one peak of expression in the Wnt phase and the other one in the Notch/Fgf phase. [file 1471-213X-13-42-S6.tiff]

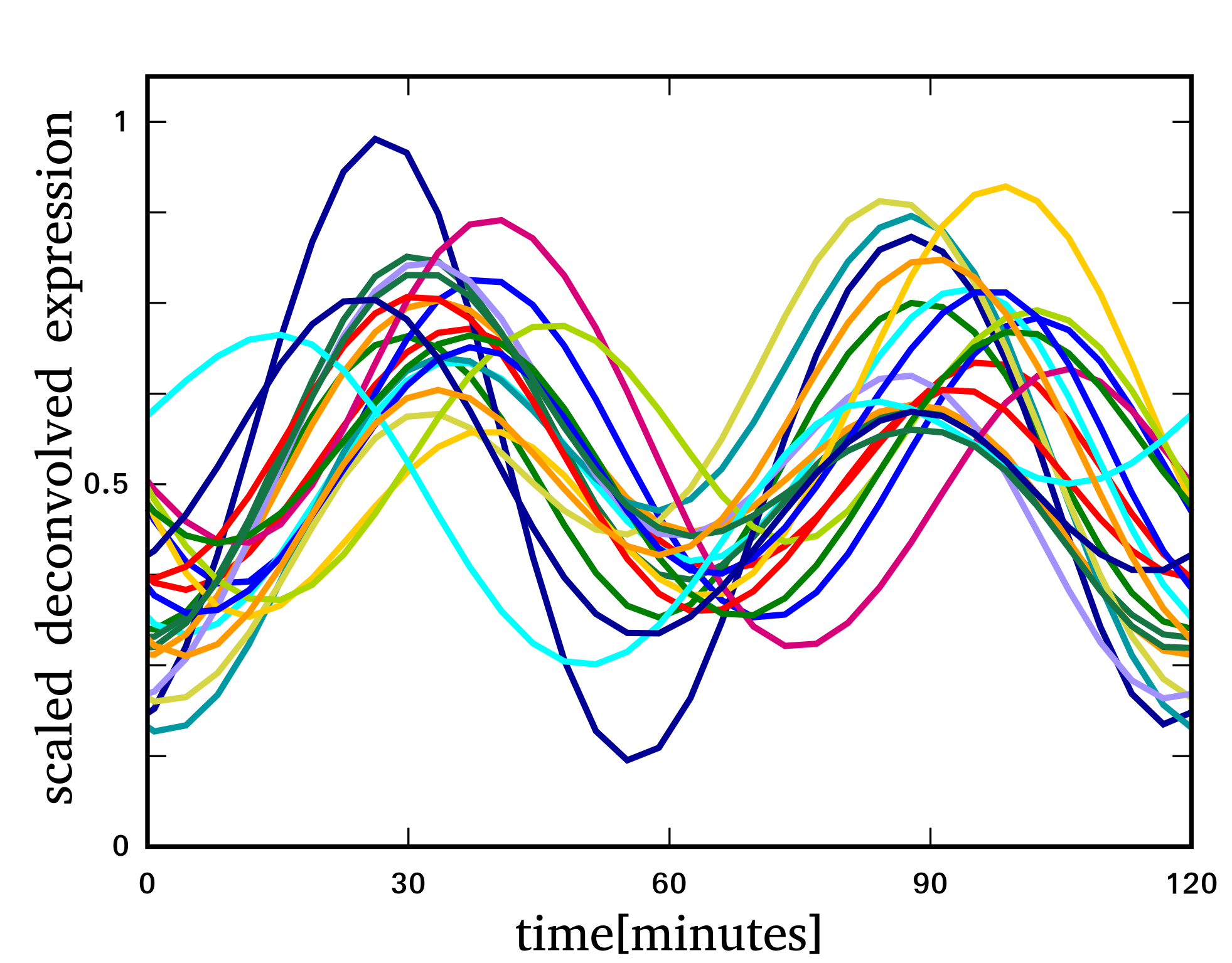

Supplement: Additional file 7: Figure S4 — Profiles of the top 20 genes with two peaks of expression. [file 1471-213X-13-42-S7.tiff]

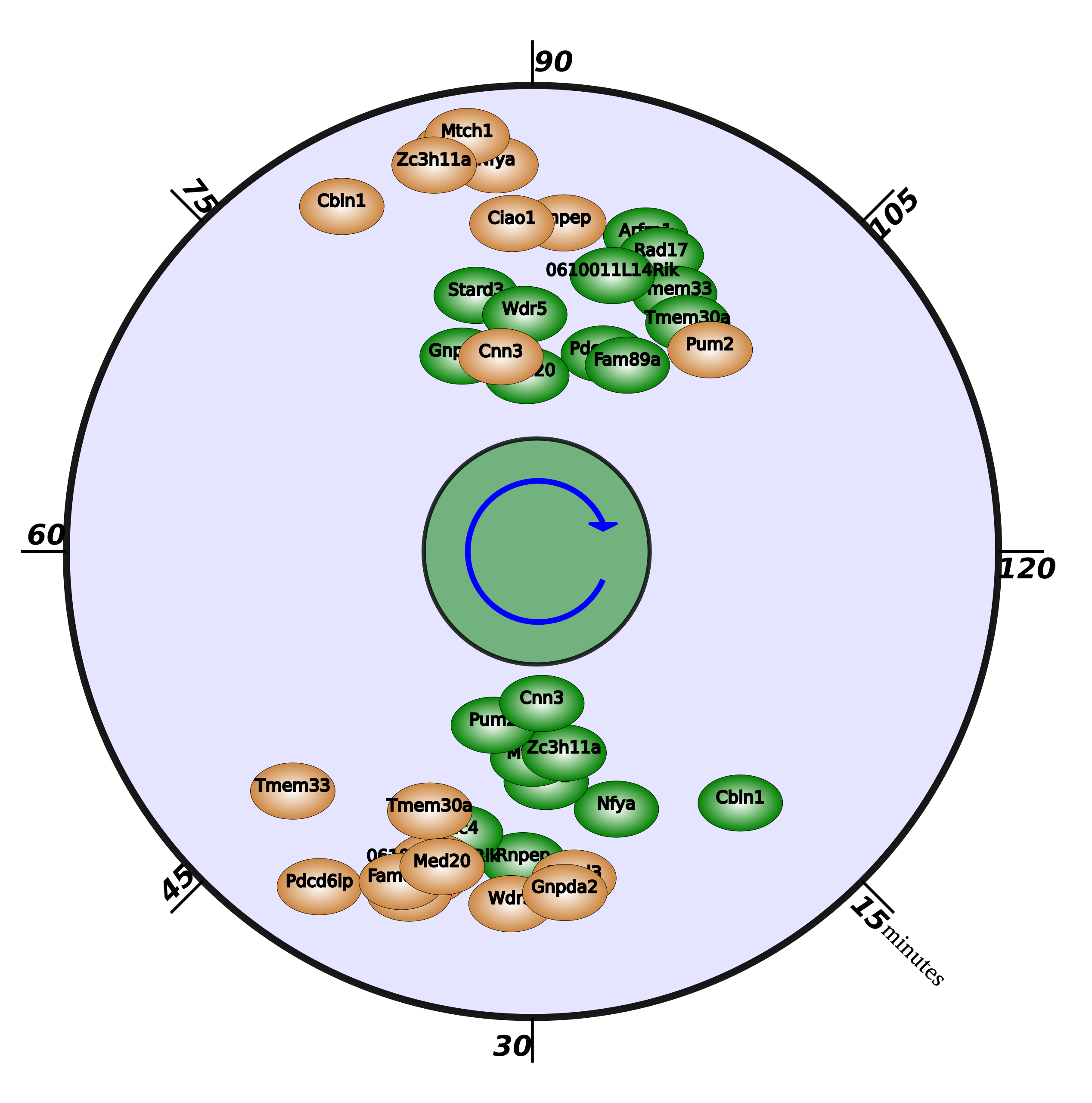

Supplement: Additional file 8: Figure S5 — Timing of the top 20 genes with two peaks of expression. [file 1471-213X-13-42-S8.tiff]

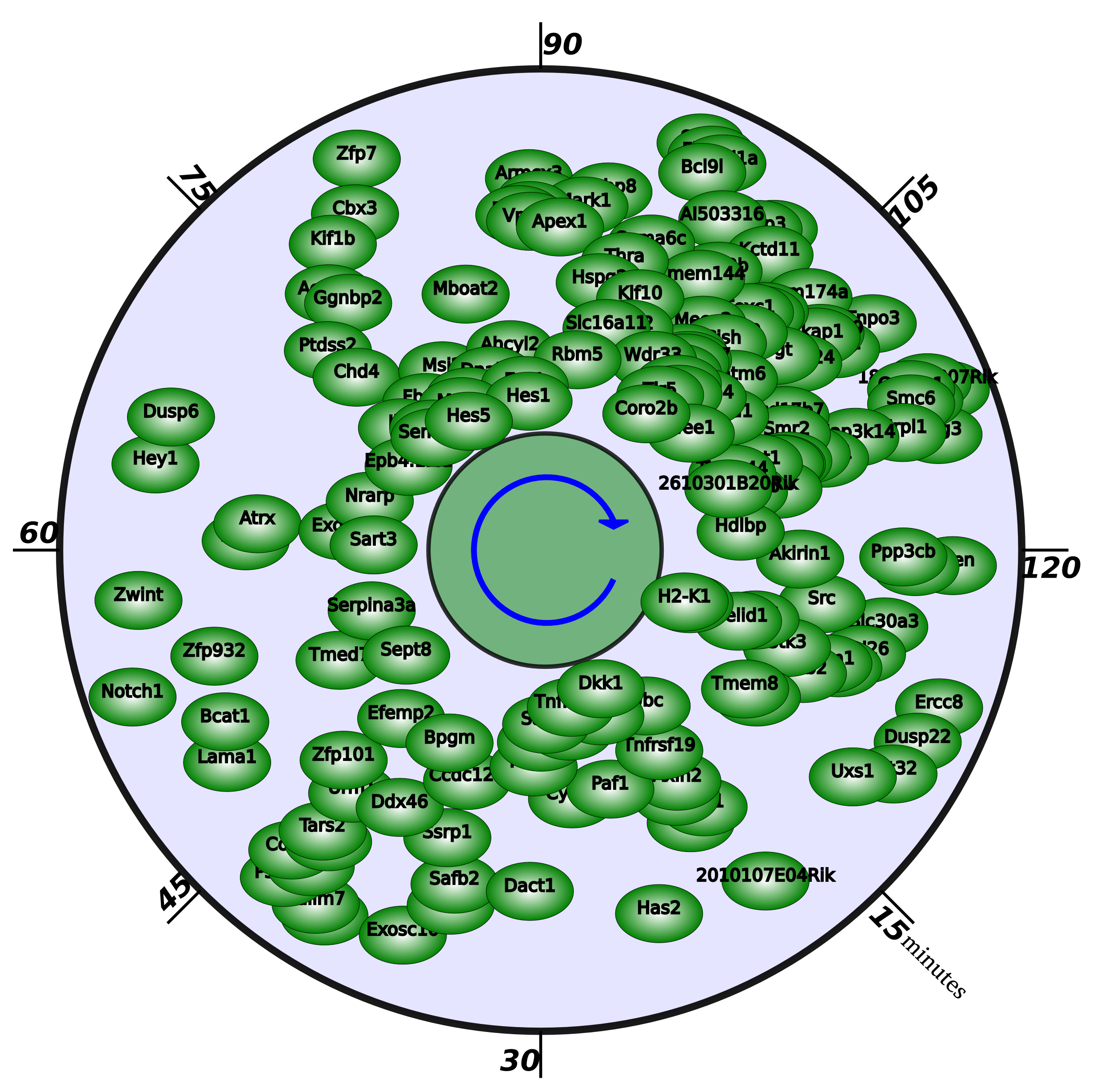

Supplement: Additional file 9: Figure S6 — Timing of genes with one peak of expression. The positions of peaks of 159 genes along the 2 h somite cycle are shown. [file 1471-213X-13-42-S9.tiff]

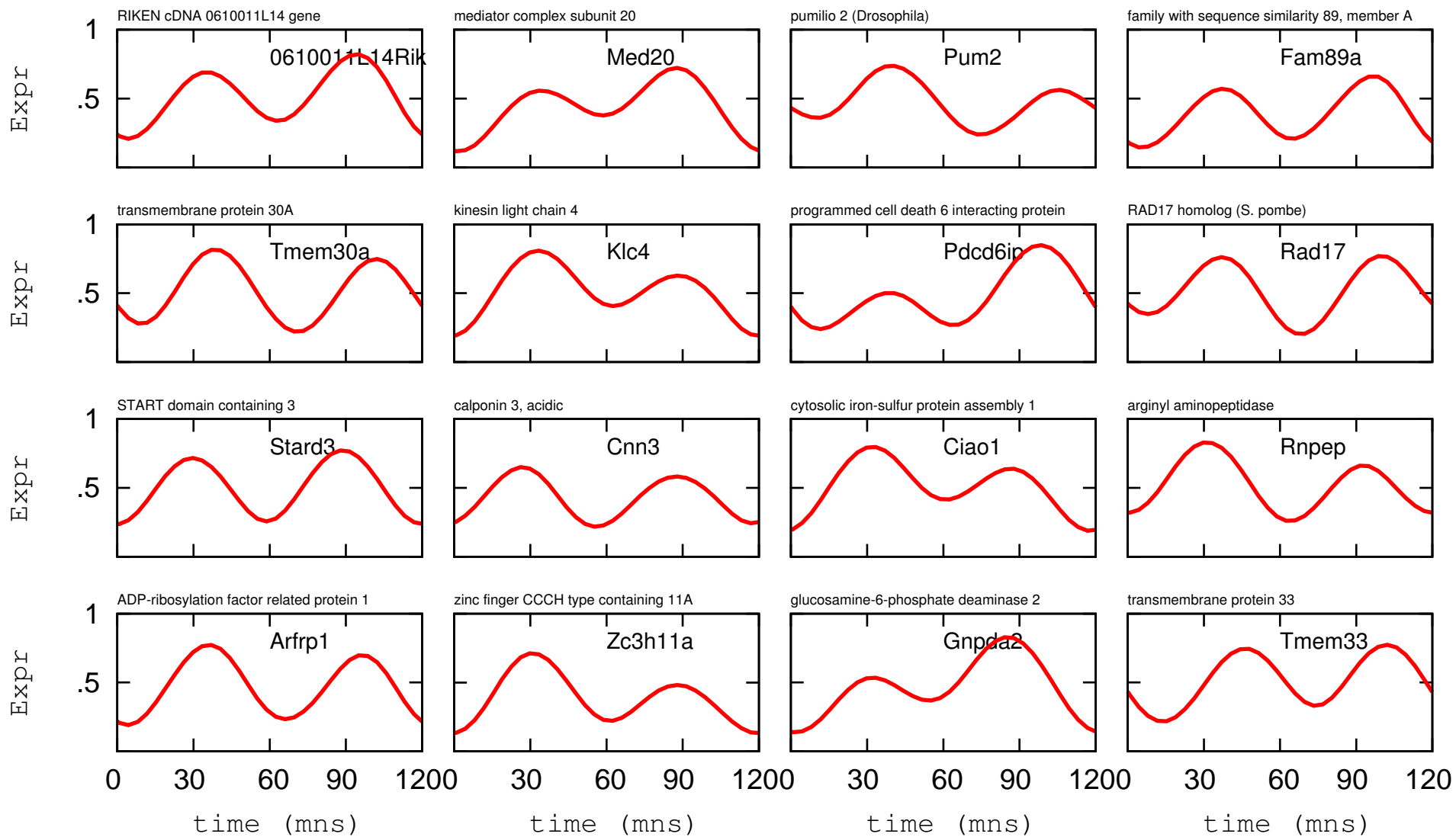

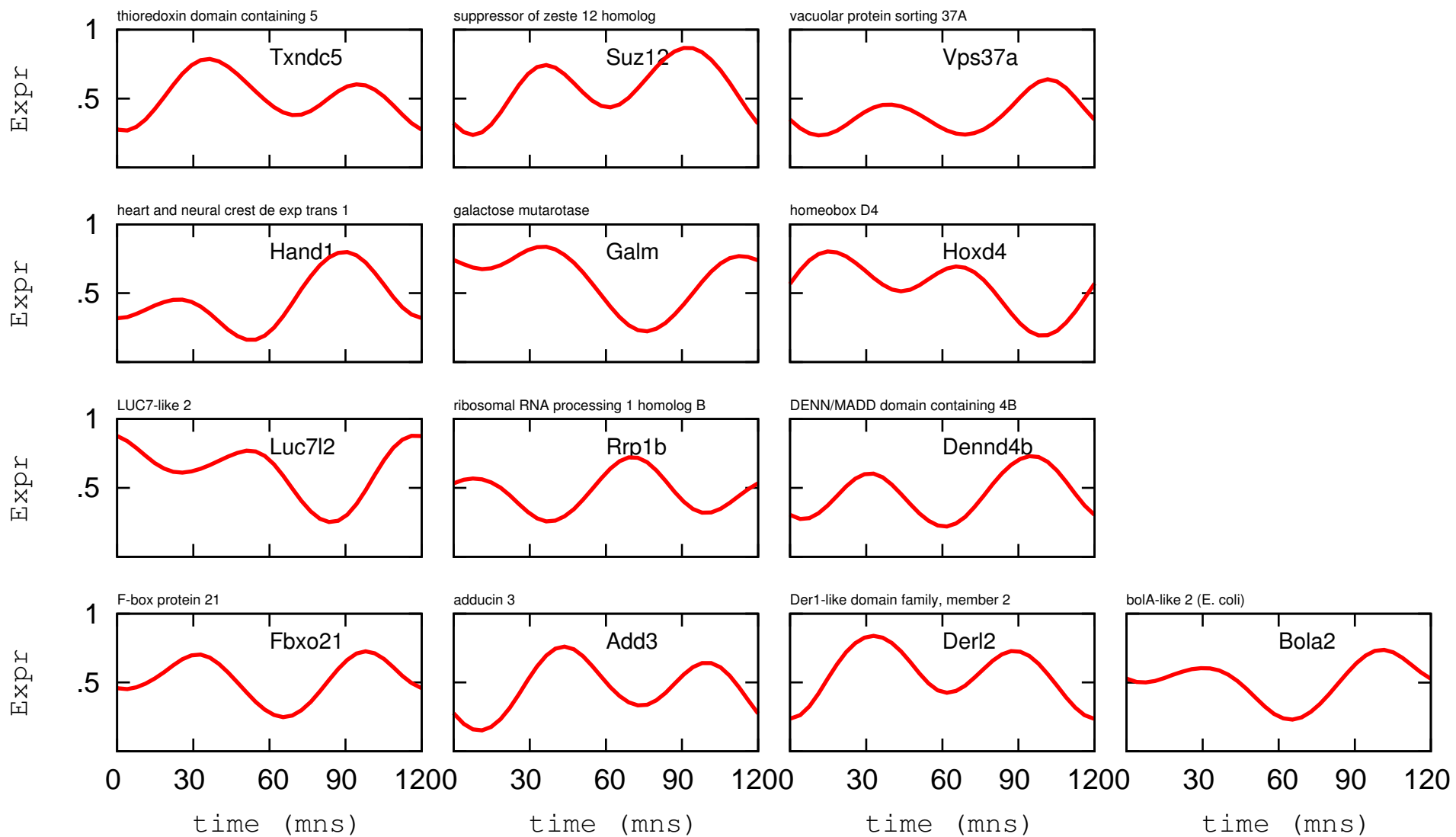

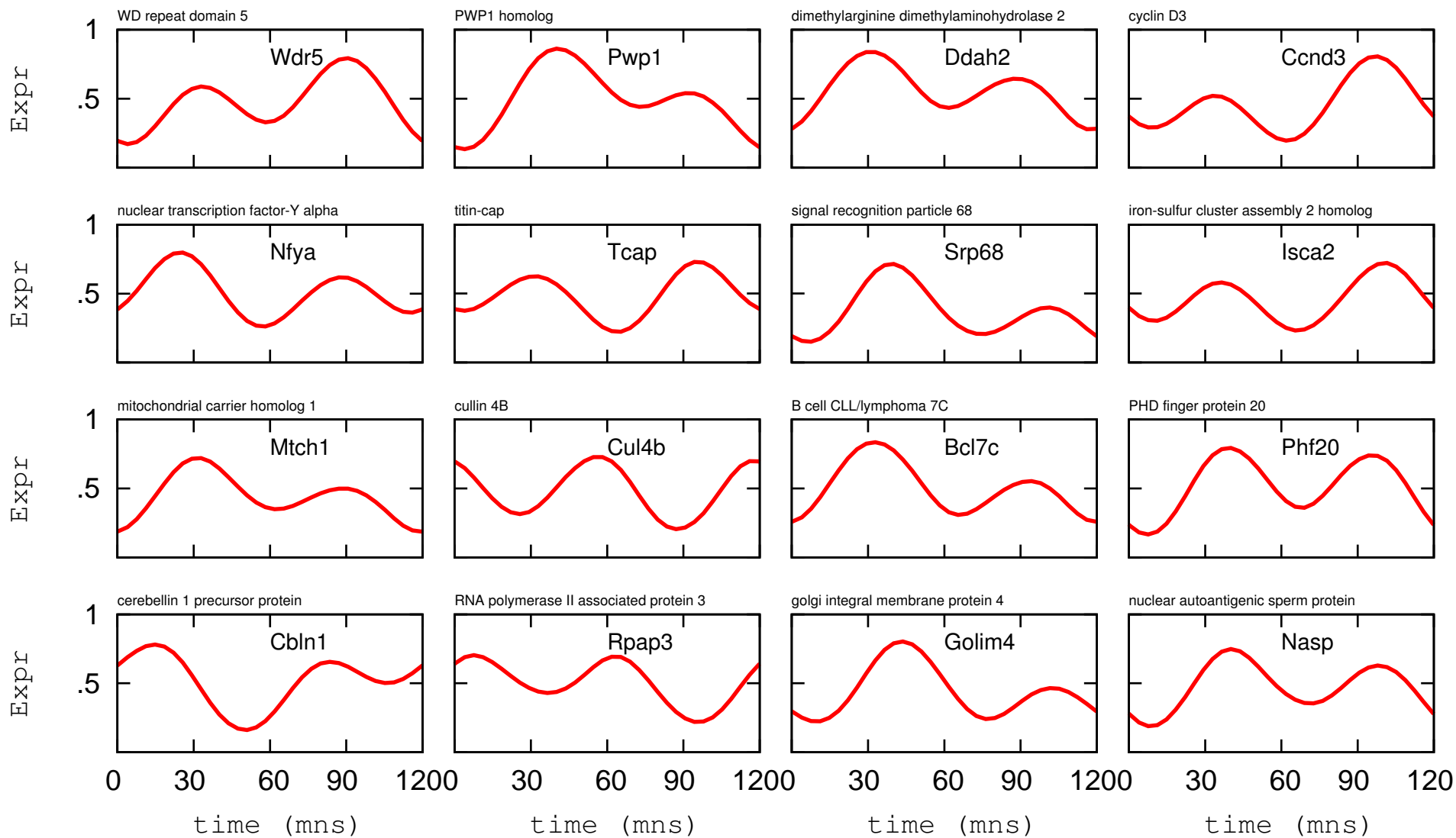

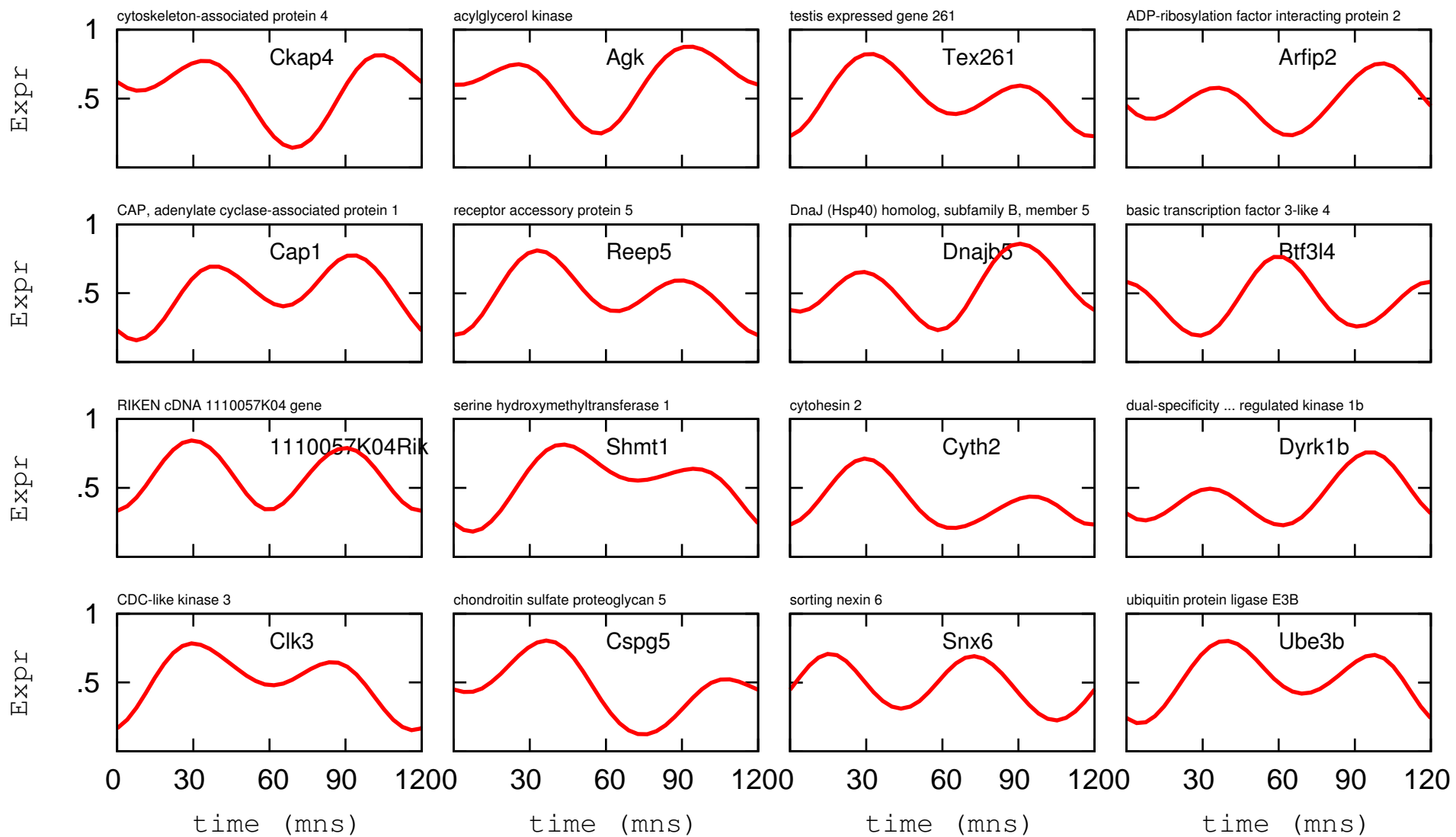

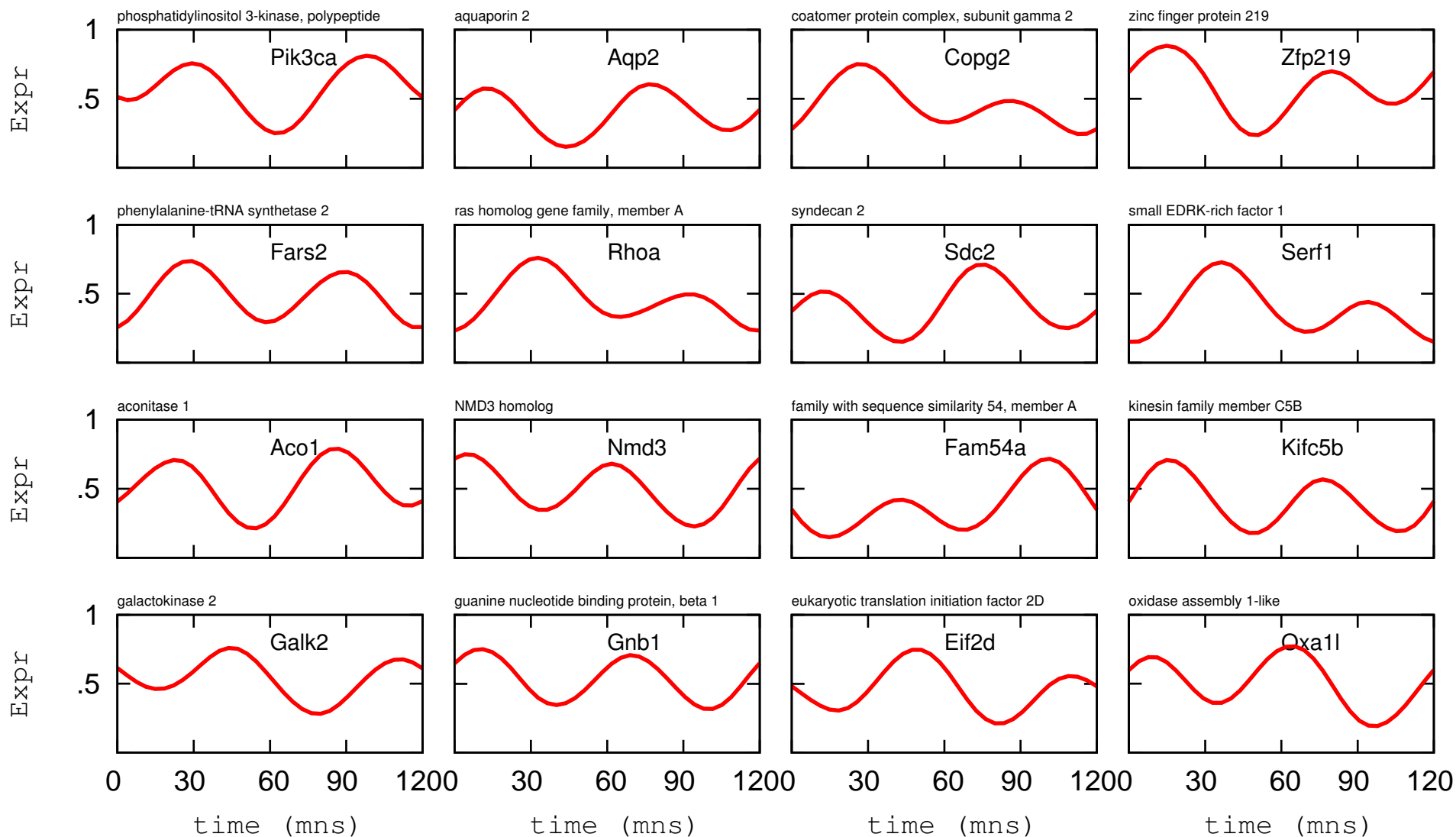

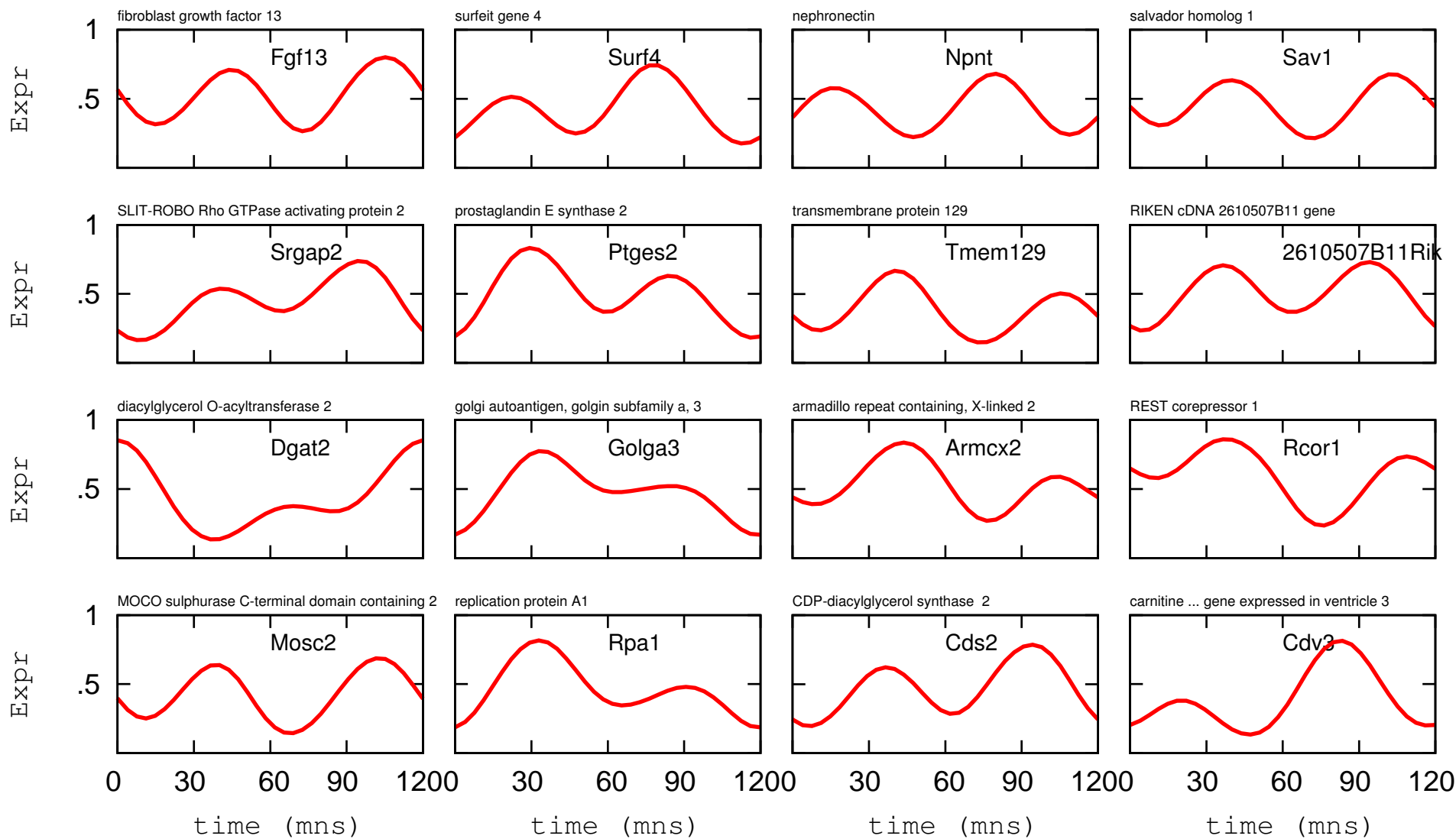

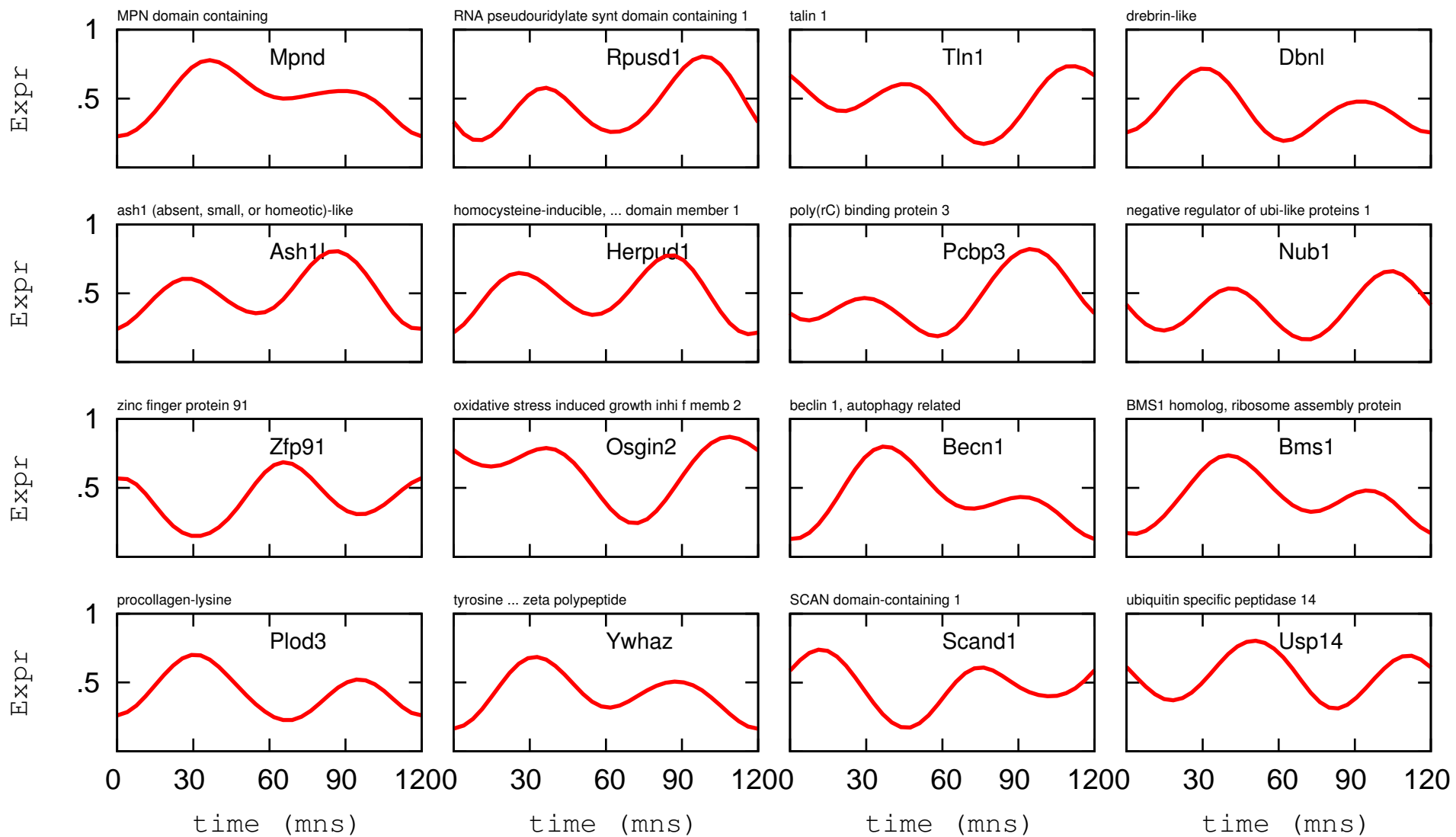

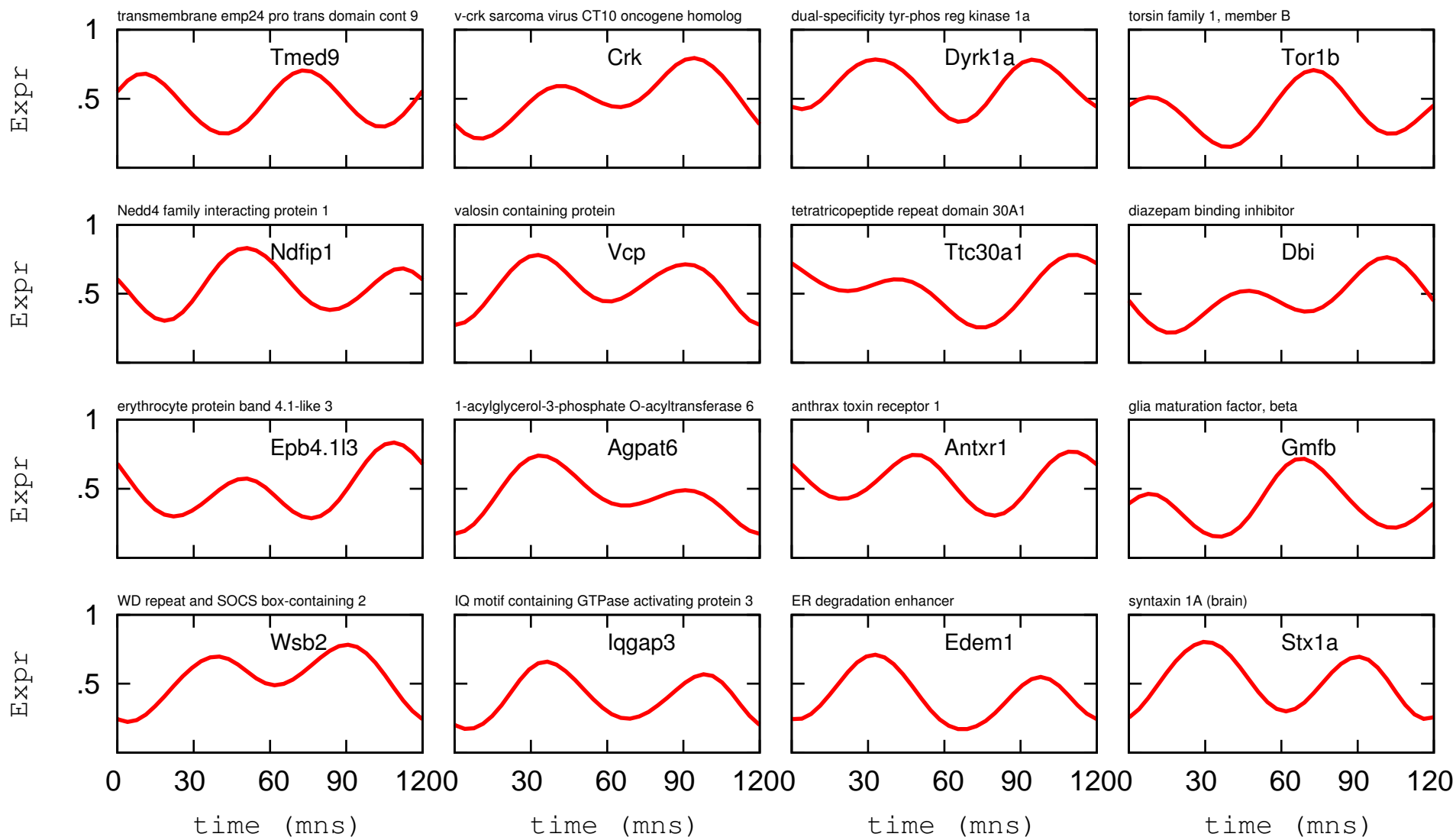

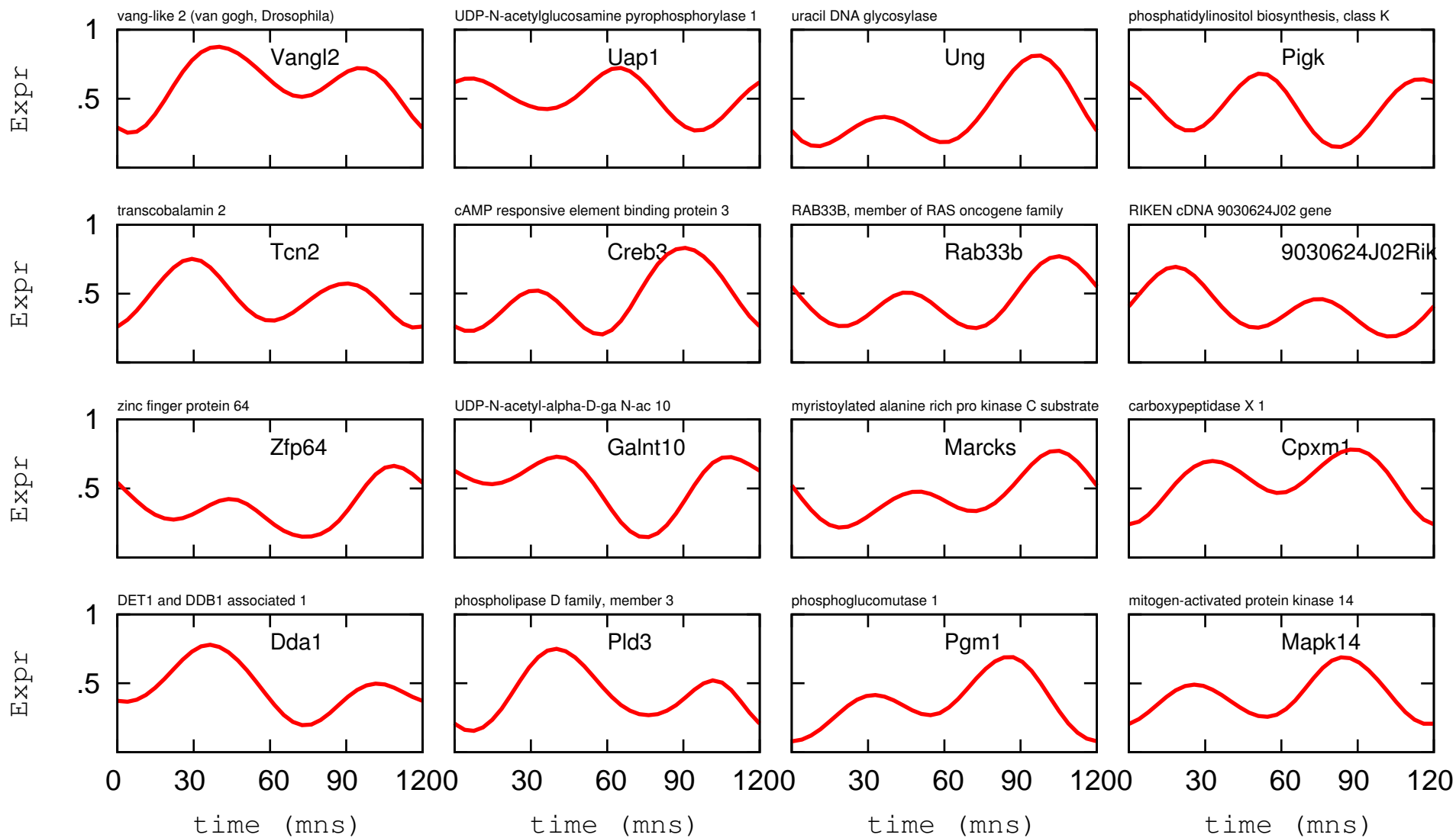

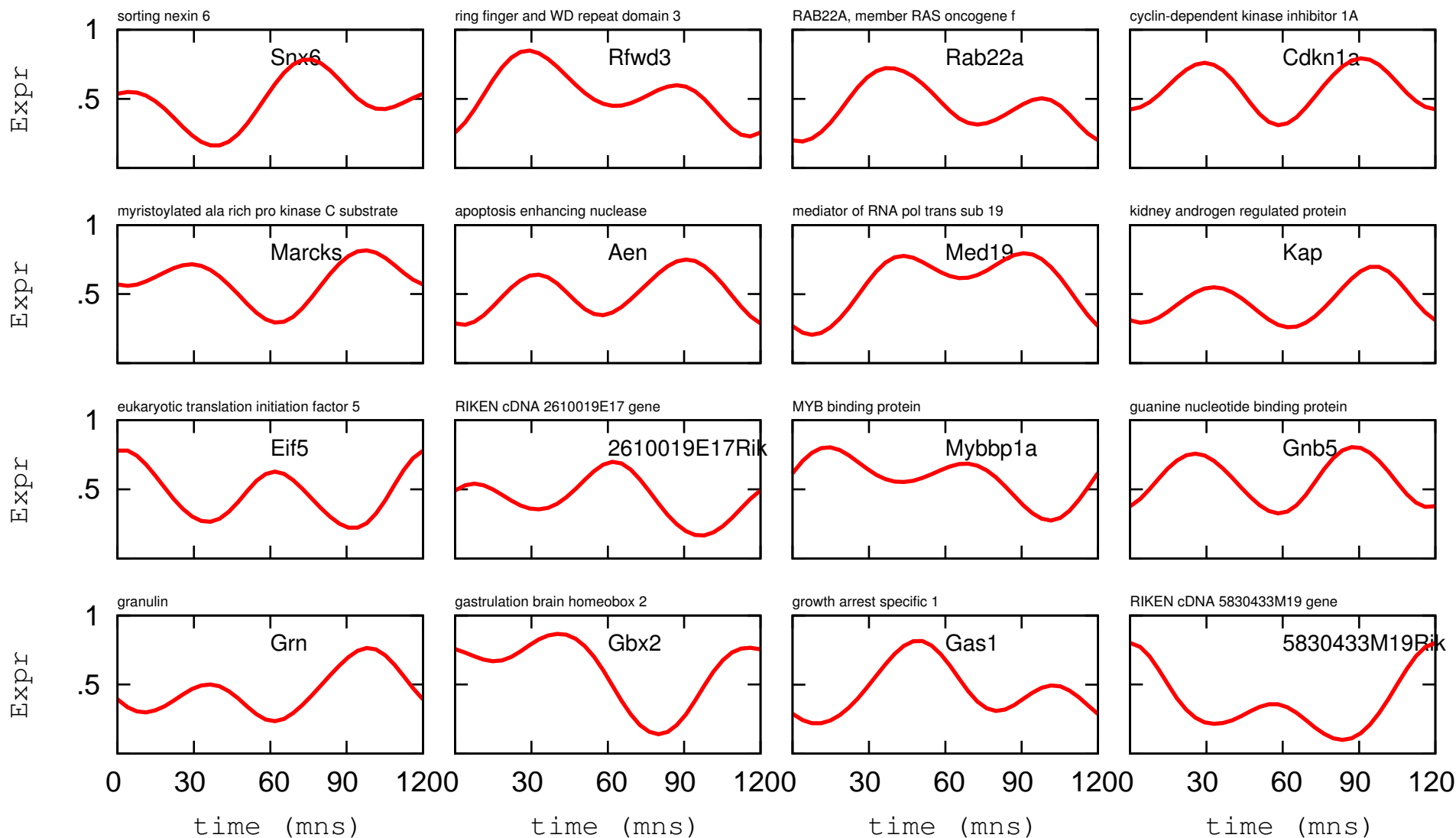

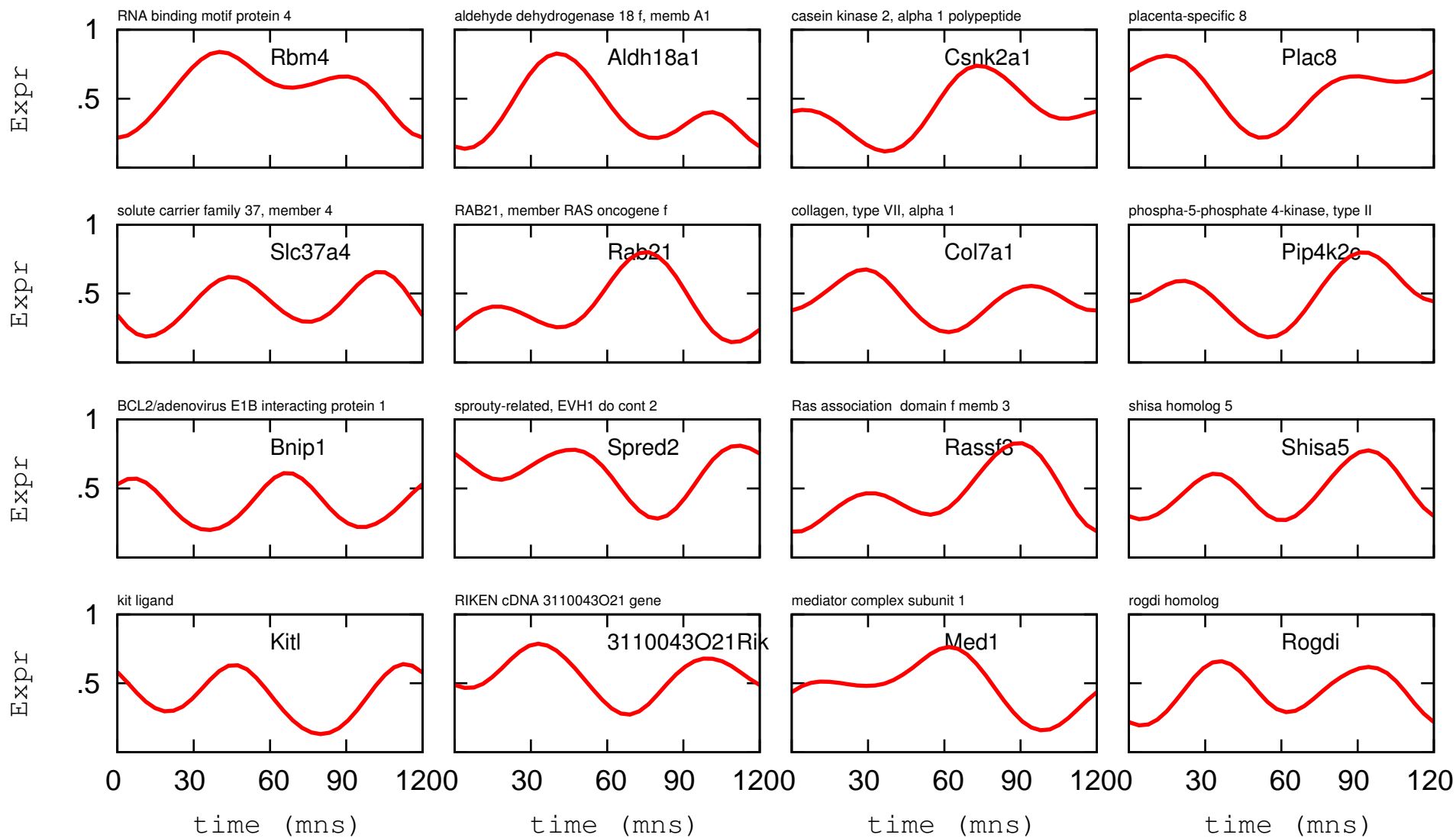

Supplement: Additional file 11: Figure S7 — Individual profile for every gene listed in the Additional file 5: Table S2. For every gene in the Additional file 5: Table S2, the deconvolved expression is displayed. [file 1471-213X-13-42-S11.pdf]

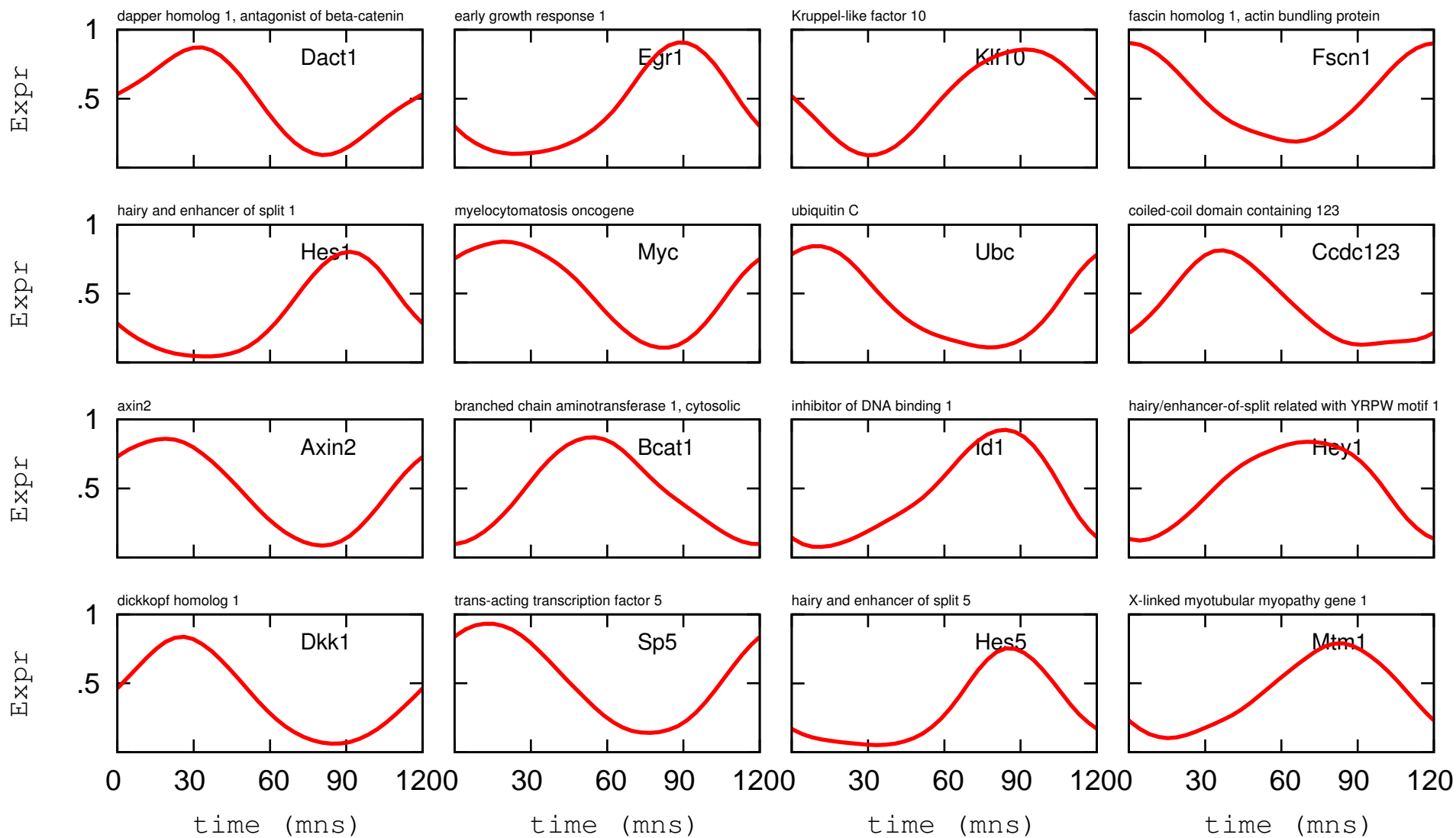

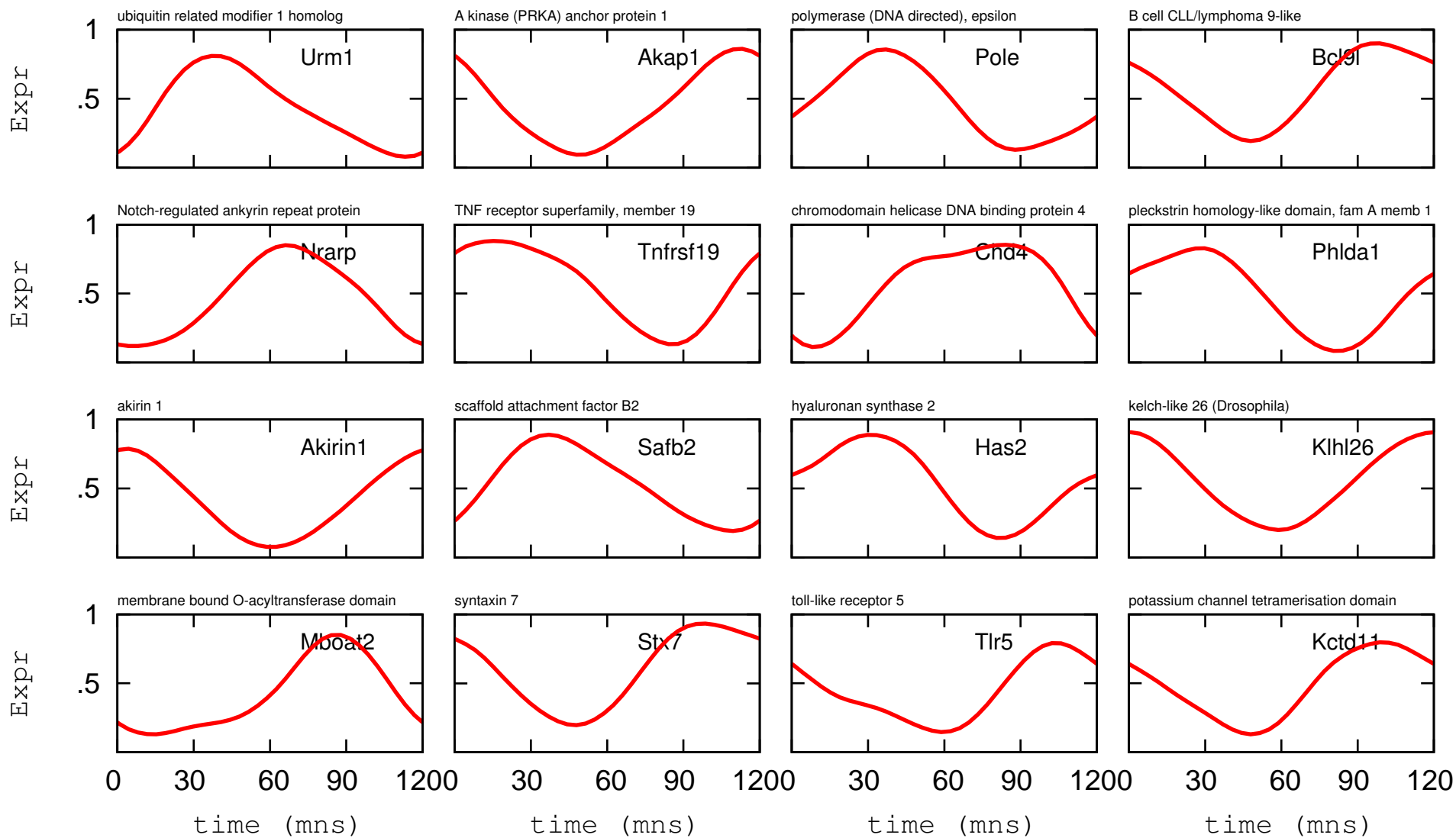

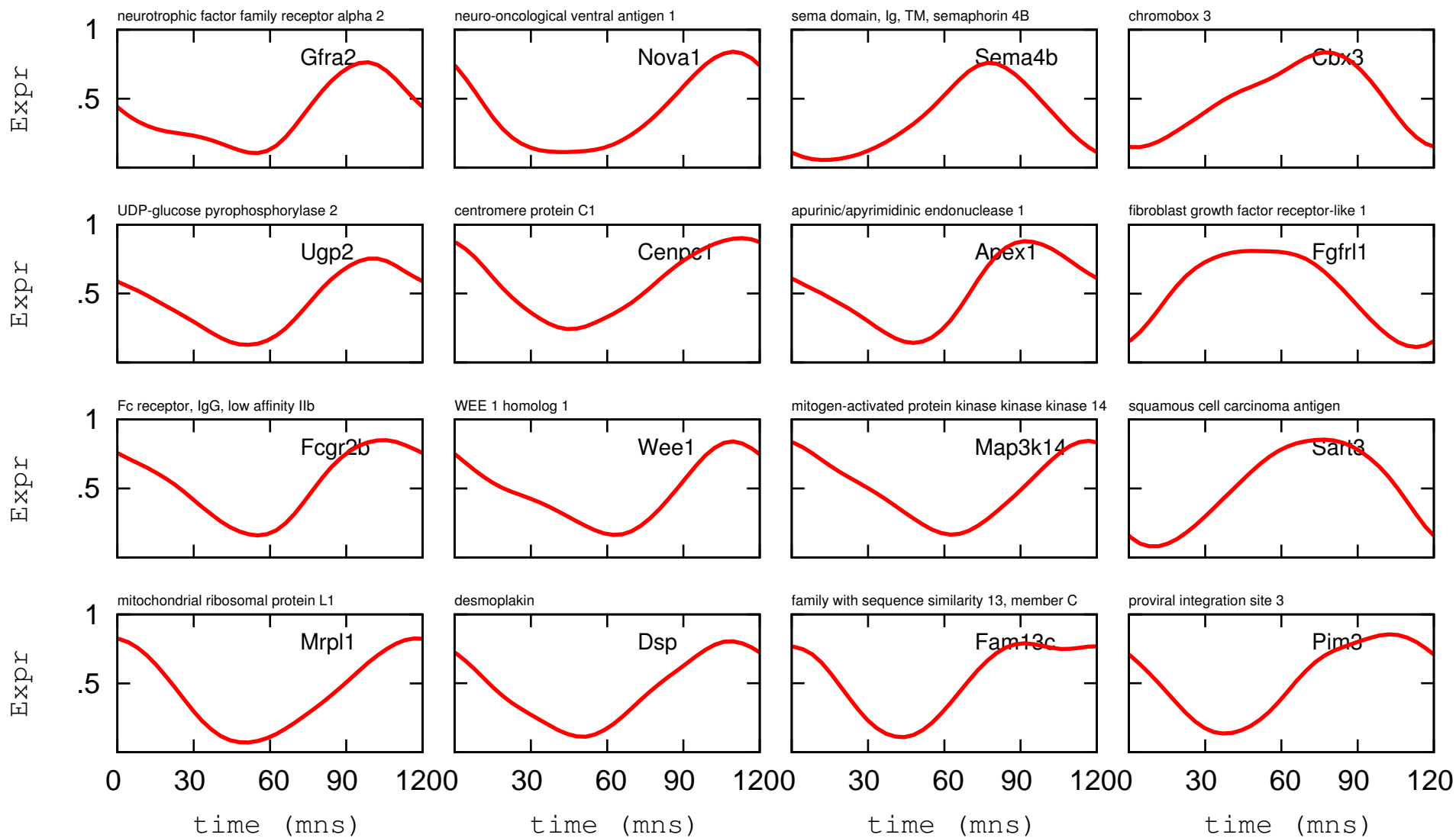

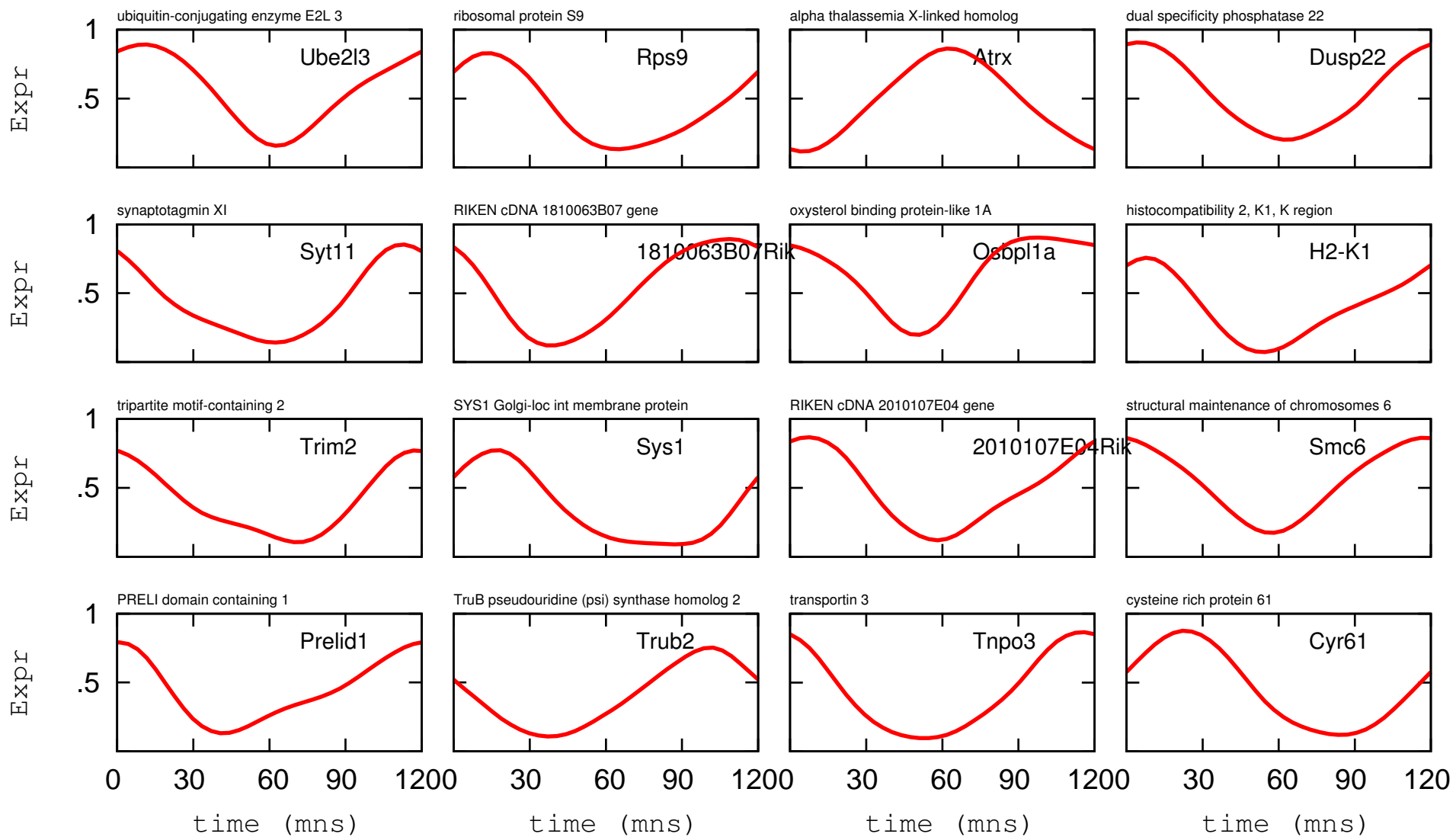

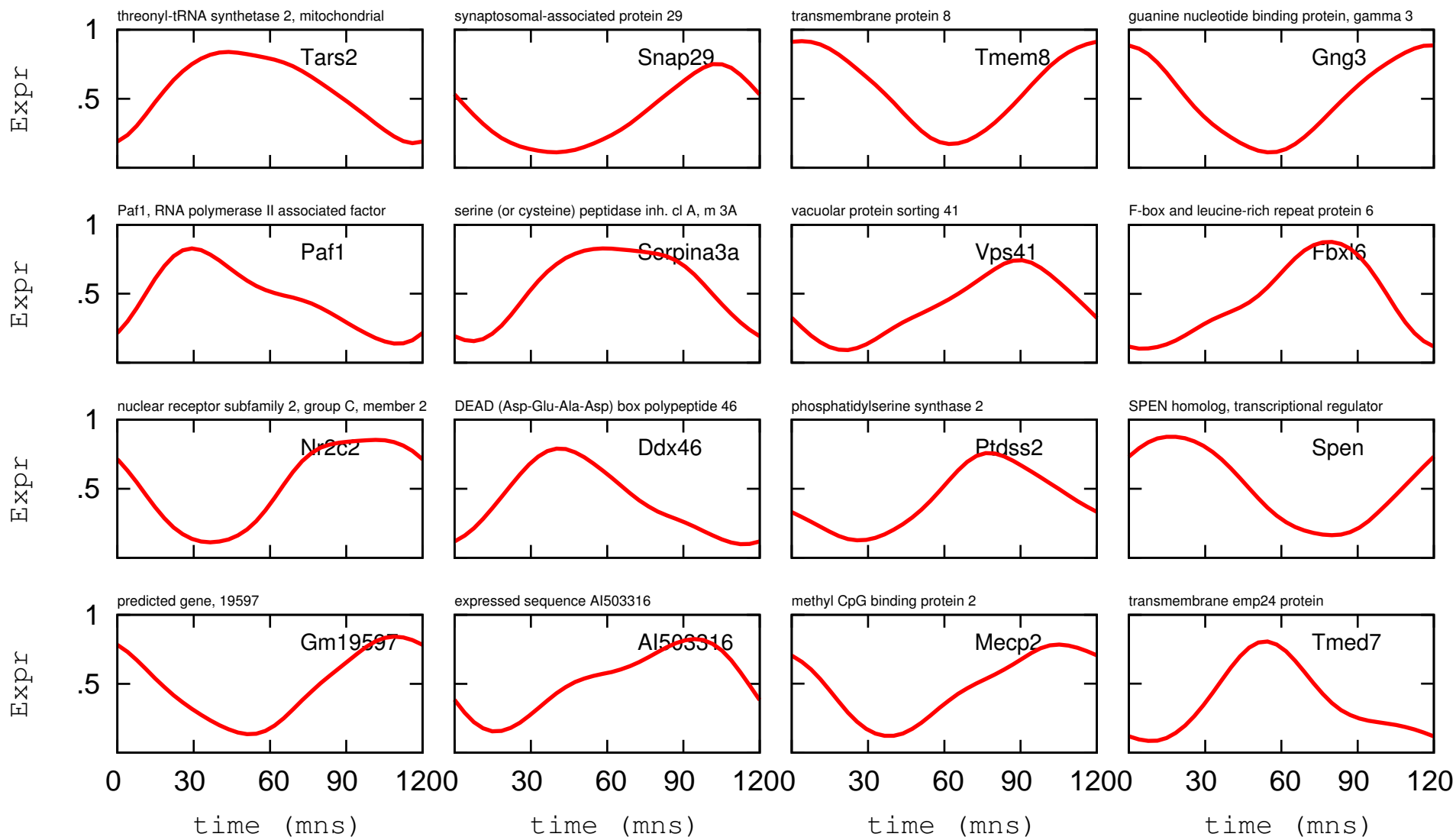

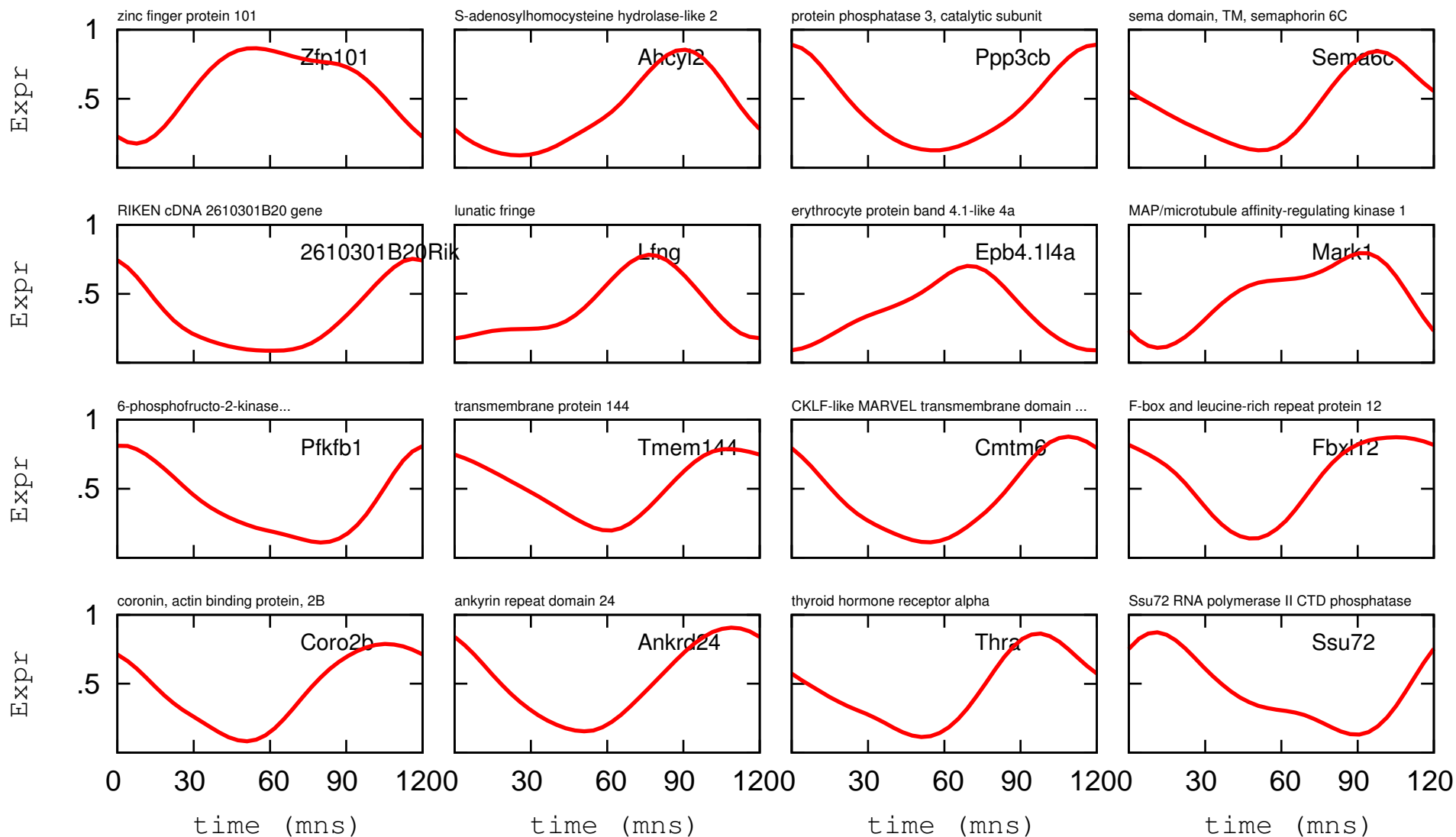

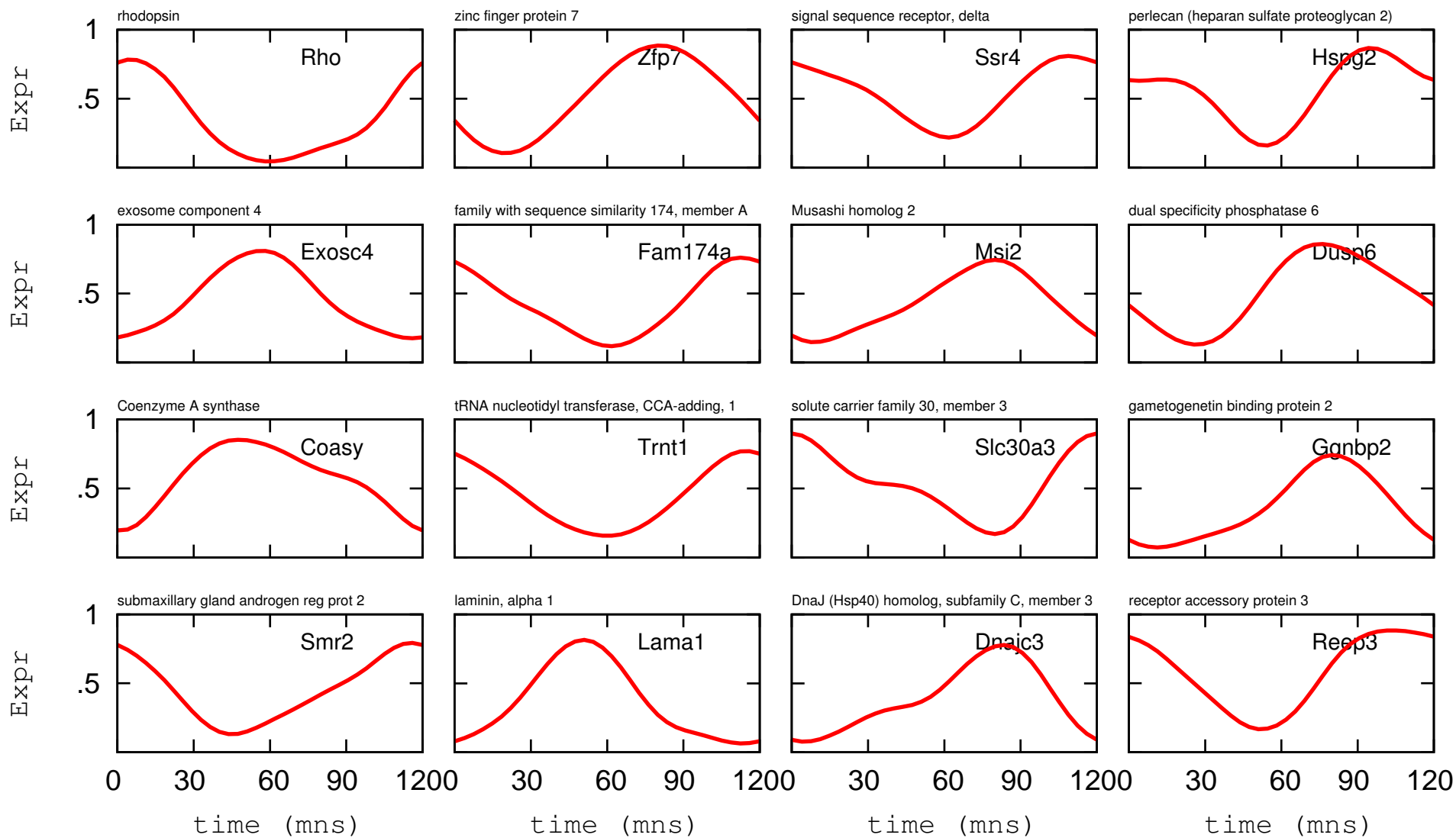

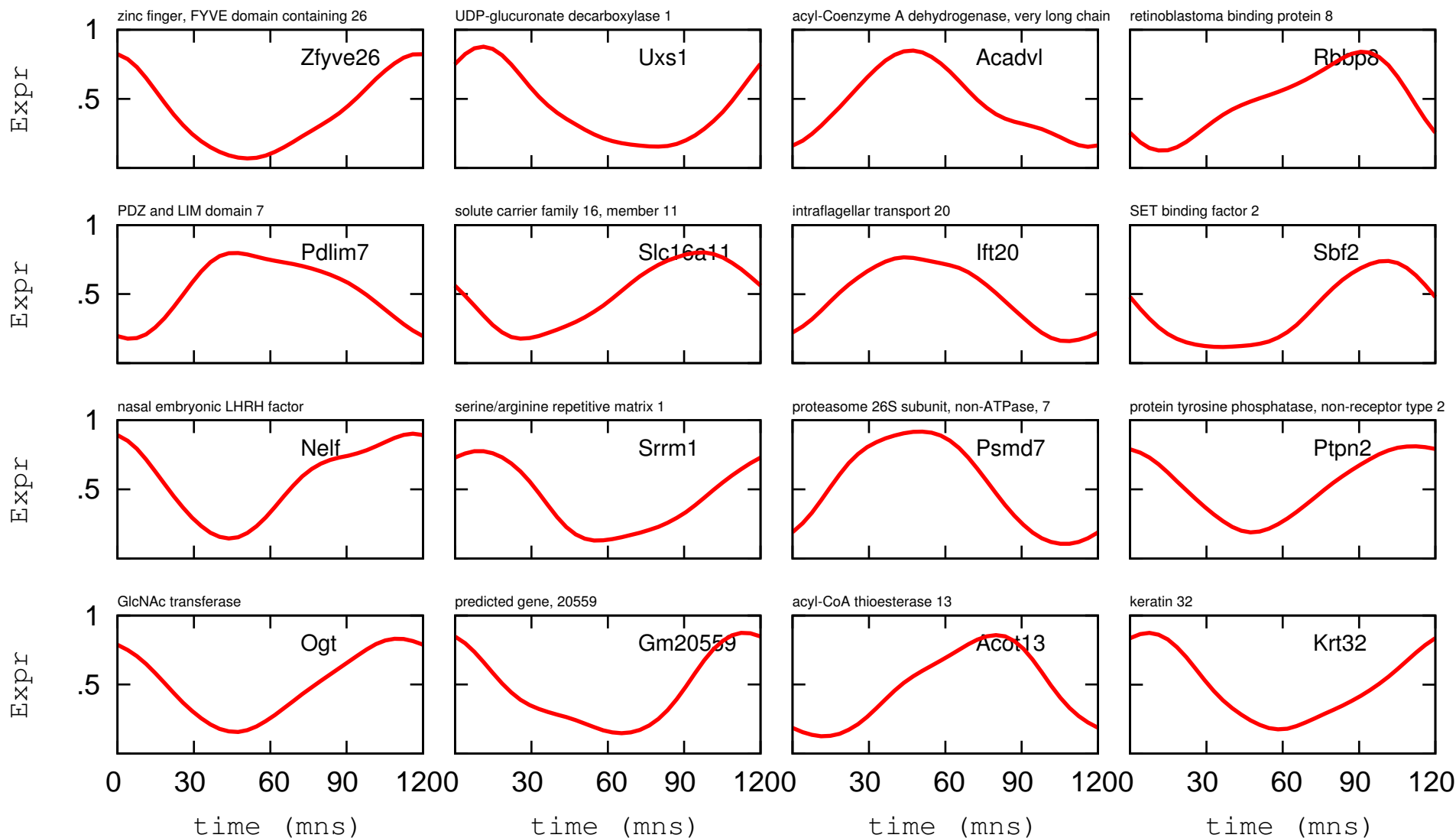

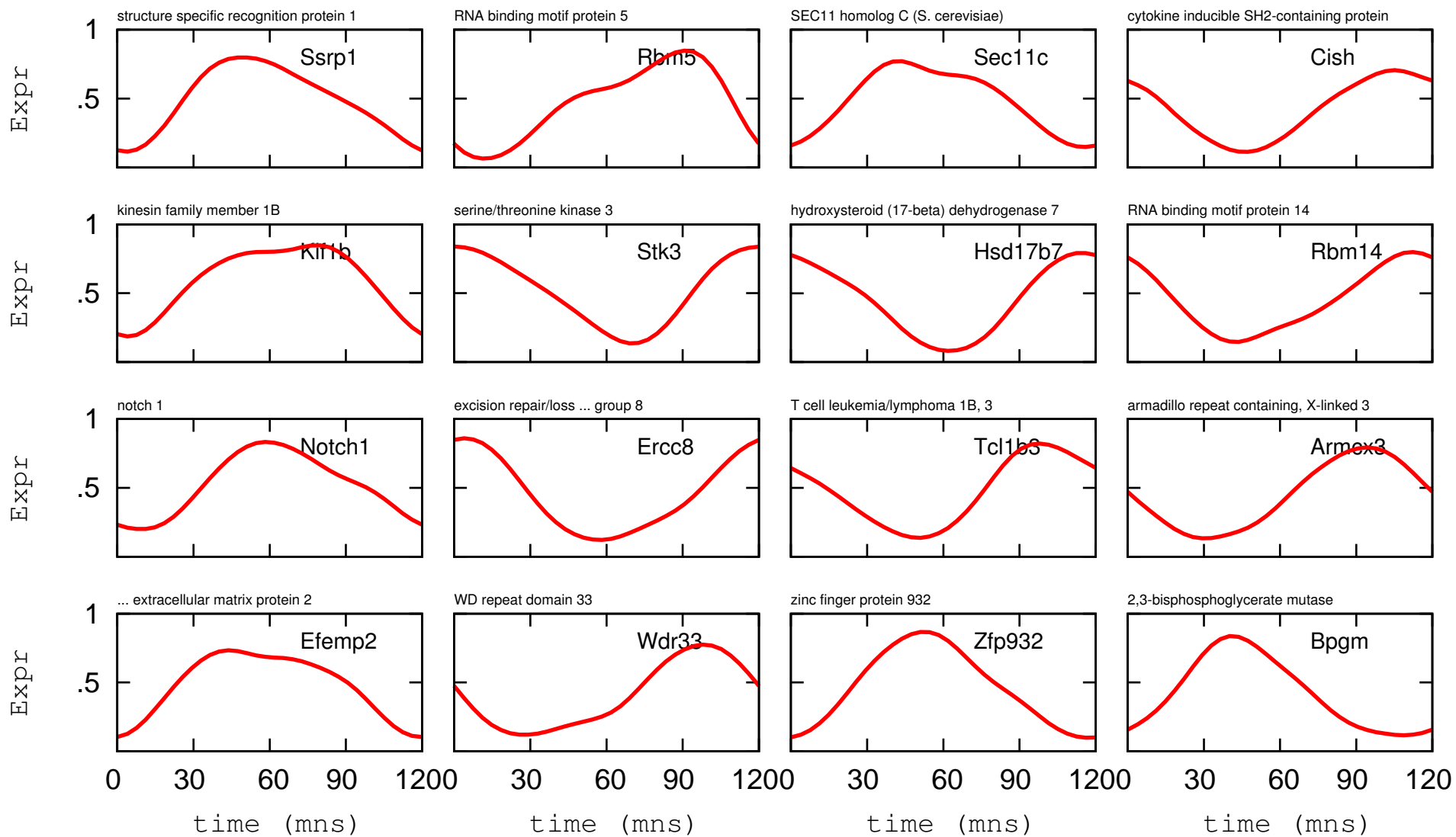

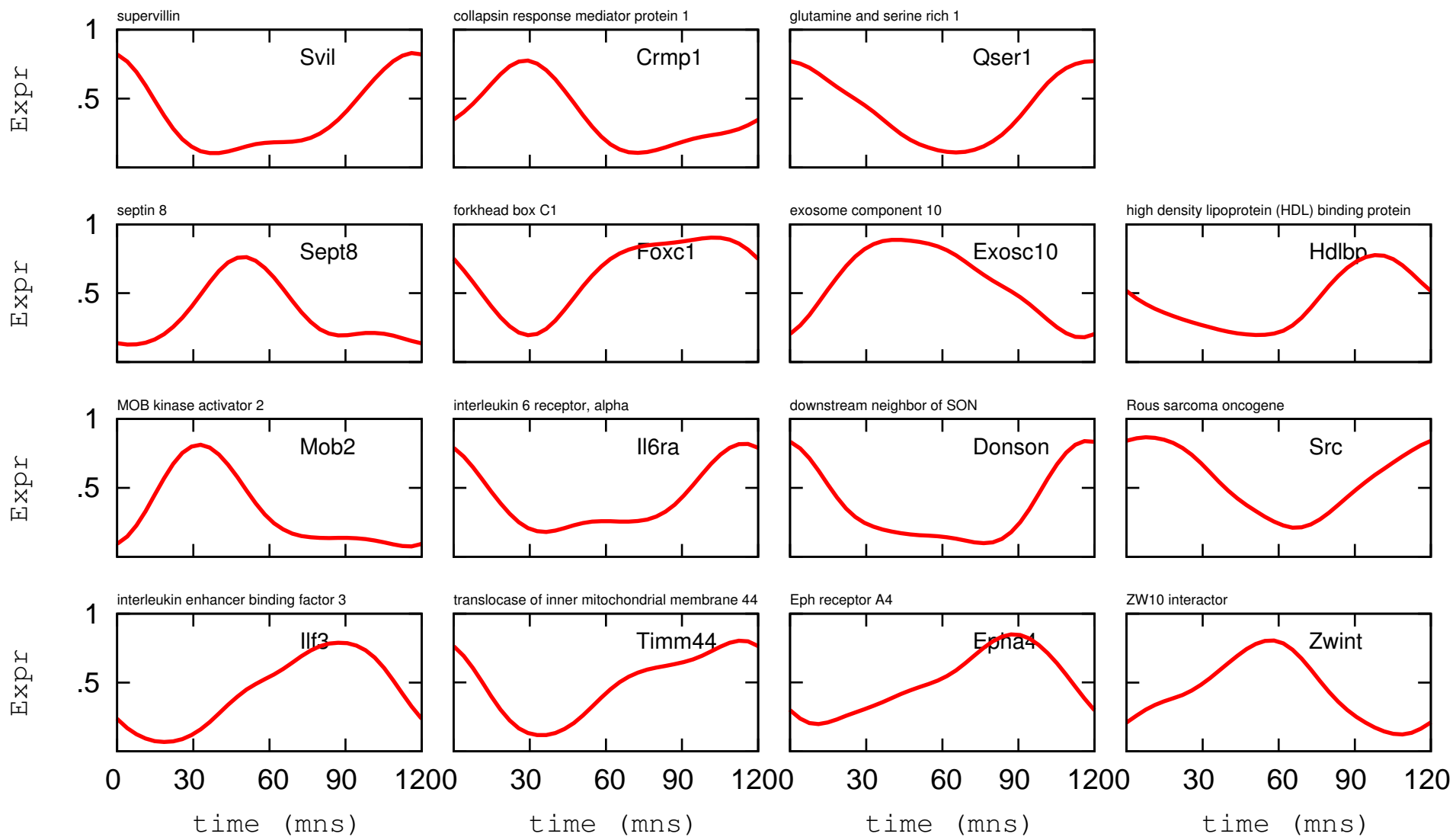

Supplement: Additional file 12: Figure S8 — Individual profile for every gene listed in the Additional file 2: Table S1. For every gene in the Additional file 2: Table S1, the deconvolved expression is displayed. [file 1471-213X-13-42-S12.pdf]
