# Supplementary material for: The precise timeline of transcriptional regulation reveals causation in mouse somitogenesis network
Source: BMC Dev Biol. 2013 Dec 5;13:42. doi: 10.1186/1471-213X-13-42 (PMC4235037; doi:10.1186/1471-213X-13-42)
Supplement: Additional file 2: Table S1 — Timing of genes known to be associated with Fgf, Wnt and Notch pathways in the data set mouse2. [file 1471-213X-13-42-S2.docx]

| **Probeset** | **Gene** | **Time (min)** |
| --- | --- | --- |
| 1420360_at | *Dkk1* | 22 **±** 2 |
| 1427600_at | *Tnfrsf19* | 21 **±** 3 |
| 1436845_at | *Axin2* | 20 **±** 1 |
| 1418102_at | *Hes1* | 88 **±** 3 |
| 1417937_at | *Dact1* | 25 **±** 6 |
| 1422914_at | *Sp5* | 16 ± 5 |
| 1424942_a_at | *Myc* | 20 ± 1 |
| 1417065_at | *Egr1* | 88 ± 2 |
| 1456010_x_at | *Hes5* | 75 ± 5 |
| 1425895_a_at | *Id1* | 79 ± 2 |
| 1415999_at | *Hey1* | 59 ± 5 |
| 1436584_at | *Spry2* | 91 ± 7 |
| 1417985_at | *Nrarp* | 53 ± 2 |
| 1416895_at | *Efna1* | 71 ± 6 |
| 1456005_a_at | *Bcl2l11* | 102 ± 1 |
| 1449169_at | *Has2* | 19 ± 5 |
| 1418835_at | *Phlda1* | 17 ± 6 |
| 1419180_at | *Bcl9l* | 67 ± 9 |
| 1416039_x_at | *Cyr61* | 19± 6 |
| 1448985_at | *Dusp22* | 3 ± 2 |
| 1417278_a_at | *Nkd1* | 55 ± 6 |
| 1415834_at | *Dusp6* | 56 ± 8 |
| 1448830_at | *Dusp1* | 81 ± 9 |
| 1449868_at | *Tbx6* | 42 ± 7 |
| 1420811_a_at | *Ctnnb1* | 8 ± 3 |
| 1449111_a_at | *Grb2* | 62 ± 4 |

**Table S1: Timing of genes known to be associated with Fgf, Wnt and Notch pathways in the data set mouse2.**
