# Supplementary material for: The precise timeline of transcriptional regulation reveals causation in mouse somitogenesis network
Source: BMC Dev Biol. 2013 Dec 5;13:42. doi: 10.1186/1471-213X-13-42 (PMC4235037; doi:10.1186/1471-213X-13-42)
Supplement: Additional file 5: Table S2 — The list of genes with two peaks of expression. The timing of genes found with two peaks of expression, ranked according to their LS p-value and the regularity of the profile. Times in minutes assume a 2 h periodicity for every transcript and errors are computed by adding to the original transcript source of noise typically found in microarray experiments. [file 1471-213X-13-42-S5.docx]

**Table S2: The list of genes with two peaks of expression**.

| **Probeset** | **Gene** | **T_1_ (min)** | **Error (min)** | **T_2_ (min)** | **Error (min)** | **LS p-value** |
| --- | --- | --- | --- | --- | --- | --- |
| 1425508_s_at | Arfrp1 | 96 | 3 | 37 | 4 | 0.00231 |
| 1417405_at | Stard3 | 91 | 4 | 28 | 3 | 0.00261 |
| 1416446_at | Tmem30a | 101 | 2 | 37 | 3 | 0.00286 |
| 1426017_a_at | 0610011L14Rik | 95 | 4 | 31 | 2 | 0.00387 |
| 1426359_at | Zc3h11a | 28 | 2 | 86 | 5 | 0.00392 |
| 1456380_x_at | Cnn3 | 29 | 1 | 84 | 3 | 0.00555 |
| 1417108_at | Klc4 | 34 | 3 | 86 | 8 | 0.00696 |
| 1448478_at | Med20 | 89 | 1 | 29 | 2 | 0.00717 |
| 1426524_at | Gnpda2 | 83 | 3 | 28 | 1 | 0.00748 |
| 1450953_at | Ciao1 | 32 | 3 | 89 | 4 | 0.00798 |
| 1448155_at | Pdcd6ip | 96 | 3 | 41 | 3 | 0.00799 |
| 1418017_at | Pum2 | 35 | 2 | 103 | 1 | 0.00808 |
| 1452053_a_at | Tmem33 | 100 | 5 | 45 | 3 | 0.00823 |
| 1451243_at | Rnpep | 31 | 3 | 92 | 2 | 0.00828 |
| 1448762_at | Rad17 | 98 | 4 | 37 | 4 | 0.00873 |
| 1427356_at | Fam89a | 100 | 2 | 37 | 3 | 0.00883 |
| 1423286_at | Cbln1 | 17 | 1 | 77 | 5 | 0.00942 |
| 1460718_s_at | Mtch1 | 29 | 1 | 87 | 9 | 0.00947 |
| 1452560_a_at | Nfya | 24 | 2 | 88 | 5 | 0.00985 |
| 1448389_at | Wdr5 | 89 | 3 | 28 | 3 | 0.0101 |
| 1448555_at | Rpap3 | 64 | 1 | 6 | 1 | 0.01054 |
| 1417453_at | Cul4b | 62 | 4 | 113 | 2 | 0.01075 |
| 1423145_a_at | Tcap | 93 | 2 | 30 | 3 | 0.01088 |
| 1454142_a_at | Pwp1 | 37 | 2 | 90 | 3 | 0.01185 |
| 1428875_at | Golim4 | 41 | 3 | 100 | 6 | 0.01208 |
| 1449523_at | Bcl7c | 32 | 3 | 98 | 4 | 0.01222 |
| 1433708_at | Srp68 | 38 | 3 | 101 | 5 | 0.01228 |
| 1416457_at | Ddah2 | 30 | 3 | 92 | 7 | 0.01248 |
| 1416043_at | Nasp | 40 | 3 | 97 | 6 | 0.01256 |
| 1426961_at | Phf20 | 96 | 4 | 37 | 3 | 0.01259 |
| 1424154_a_at | Isca2 | 101 | 2 | 30 | 2 | 0.01305 |
| 1415907_at | Ccnd3 | 96 | 5 | 29 | 1 | 0.0137 |
| 1423849_a_at | Clk3 | 25 | 2 | 89 | 7 | 0.01411 |
| 1428085_at | 1110057K04Rik | 30 | 3 | 93 | 6 | 0.01441 |
| 1417461_at | Cap1 | 35 | 1 | 91 | 2 | 0.01451 |
| 1426754_x_at | Ckap4 | 100 | 4 | 35 | 4 | 0.01453 |
| 1450713_at | Cspg5 | 36 | 4 | 104 | 3 | 0.01461 |
| 1425177_at | Shmt1 | 41 | 3 | 102 | 6 | 0.01462 |
| 1426376_at | Reep5 | 32 | 2 | 85 | 6 | 0.01463 |
| 1424582_at | Agk | 91 | 7 | 29 | 2 | 0.01471 |
| 1451602_at | Snx6 | 16 | 1 | 71 | 1 | 0.01472 |
| 1450103_a_at | Cyth2 | 30 | 2 | 97 | 5 | 0.01505 |
| 1450436_s_at | Dnajb5 | 88 | 3 | 24 | 2 | 0.01541 |
| 1450965_at | Tex261 | 27 | 3 | 91 | 5 | 0.0155 |
| 1452149_at | Ube3b | 43 | 3 | 100 | 3 | 0.0158 |
| 1422254_a_at | Dyrk1b | 96 | 3 | 29 | 1 | 0.01606 |
| 1451223_a_at | Btf3l4 | 59 | 2 | 114 | 1 | 0.01606 |
| 1424240_at | Arfip2 | 101 | 3 | 42 | 3 | 0.01636 |
| 1455798_at | Galk2 | 44 | 2 | 112 | 5 | 0.01637 |
| 1423644_at | Aco1 | 84 | 3 | 27 | 1 | 0.01639 |
| 1431354_a_at | Fars2 | 29 | 2 | 87 | 4 | 0.01658 |
| 1423144_at | Pik3ca | 96 | 5 | 31 | 2 | 0.01674 |
| 1417432_a_at | Gnb1 | 66 | 2 | 13 | 1 | 0.01698 |
| 1448133_at | Nmd3 | 63 | 2 | 10 | 1 | 0.01708 |
| 1450632_at | Rhoa | 31 | 2 | 94 | 5 | 0.01725 |
| 1418903_at | Aqp2 | 73 | 1 | 11 | 1 | 0.01755 |
| 1455797_x_at | Eif2d | 47 | 4 | 107 | 2 | 0.01756 |
| 1432013_a_at | Fam54a | 100 | 3 | 41 | 3 | 0.01868 |
| 1417011_at | Sdc2 | 73 | 2 | 13 | 1 | 0.0189 |
| 1448761_a_at | Copg2 | 28 | 2 | 83 | 5 | 0.01945 |
| 1423738_at | Oxa1l | 70 | 5 | 11 | 1 | 0.01965 |
| 1449877_s_at | Kifc5b | 13 | 1 | 74 | 1 | 0.02094 |
| 1454907_at | Serf1 | 35 | 2 | 91 | 1 | 0.02099 |
| 1424301_at | Zfp219 | 11 | 1 | 76 | 3 | 0.02118 |
| 1416766_at | Mosc2 | 42 | 1 | 104 | 4 | 0.02123 |
| 1422678_at | Dgat2 | 115 | 2 | 7 | 1 | 0.02126 |
| 1429884_at | Srgap2 | 99 | 4 | 32 | 3 | 0.02219 |
| 1418497_at | Fgf13 | 104 | 5 | 45 | 4 | 0.02225 |
| 1423293_at | Rpa1 | 34 | 3 | 89 | 6 | 0.02232 |
| 1419159_at | Golga3 | 32 | 2 | 90 | 5 | 0.02281 |
| 1417591_at | Ptges2 | 30 | 3 | 84 | 4 | 0.02298 |
| 1436797_a_at | Surf4 | 76 | 2 | 19 | 1 | 0.02307 |
| 1438957_x_at | Cds2 | 96 | 7 | 33 | 3 | 0.02353 |
| 1415780_a_at | Armcx2 | 47 | 3 | 101 | 3 | 0.02441 |
| 1415715_at | Tmem129 | 43 | 2 | 107 | 5 | 0.02453 |
| 1426560_a_at | Npnt | 77 | 2 | 12 | 1 | 0.02472 |
| 1451100_a_at | Cdv3 | 82 | 4 | 18 | 1 | 0.02489 |
| 1434336_s_at | Rcor1 | 31 | 3 | 107 | 3 | 0.02501 |
| 1426820_at | 2610507B11Rik | 90 | 4 | 39 | 3 | 0.0251 |
| 1448204_at | Sav1 | 101 | 2 | 40 | 4 | 0.02521 |
| 1415901_at | Plod3 | 30 | 1 | 95 | 4 | 0.02567 |
| 1429615_at | Zfp91 | 64 | 3 | 5 | 1 | 0.02716 |
| 1450072_at | Ash1l | 83 | 3 | 28 | 2 | 0.02736 |
| 1451357_at | Mpnd | 32 | 2 | 99 | 4 | 0.0274 |
| 1448218_s_at | Ywhaz | 29 | 1 | 89 | 4 | 0.02755 |
| 1424289_at | Osgin2 | 36 | 3 | 106 | 3 | 0.02787 |
| 1435626_a_at | Herpud1 | 85 | 2 | 19 | 1 | 0.0279 |
| 1453253_a_at | Rpusd1 | 97 | 4 | 33 | 2 | 0.02814 |
| 1448868_at | Scand1 | 7 | 1 | 73 | 2 | 0.02818 |
| 1460320_at | Becn1 | 37 | 3 | 89 | 7 | 0.0285 |
| 1417526_at | Pcbp3 | 95 | 3 | 29 | 1 | 0.02854 |
| 1448402_at | Tln1 | 107 | 3 | 51 | 2 | 0.02868 |
| 1416208_at | Usp14 | 46 | 4 | 110 | 2 | 0.03019 |
| 1433636_at | Bms1 | 40 | 3 | 96 | 4 | 0.03039 |
| 1452776_a_at | Nub1 | 104 | 1 | 42 | 2 | 0.03078 |
| 1460334_at | Dbnl | 31 | 2 | 101 | 8 | 0.03084 |
| 1421847_at | Wsb2 | 91 | 4 | 41 | 3 | 0.03089 |
| 1426010_a_at | Epb4.1l3 | 107 | 3 | 51 | 2 | 0.03097 |
| 1424820_a_at | Ndfip1 | 49 | 4 | 110 | 6 | 0.03106 |
| 1448268_at | Tmed9 | 10 | 1 | 72 | 3 | 0.03186 |
| 1434850_at | Iqgap3 | 31 | 2 | 101 | 3 | 0.03195 |
| 1450776_at | Agpat6 | 29 | 1 | 89 | 6 | 0.03196 |
| 1423030_at | Vcp | 31 | 2 | 95 | 8 | 0.03217 |
| 1416201_at | Crk | 91 | 3 | 41 | 2 | 0.03238 |
| 1424065_at | Edem1 | 29 | 1 | 95 | 3 | 0.03286 |
| 1451446_at | Antxr1 | 107 | 3 | 48 | 2 | 0.03303 |
| 1424012_at | Ttc30a1 | 109 | 2 | 37 | 3 | 0.03308 |
| 1451977_at | Dyrk1a | 92 | 2 | 32 | 4 | 0.03315 |
| 1437390_x_at | Stx1a | 30 | 3 | 92 | 4 | 0.03342 |
| 1448571_a_at | Gmfb | 65 | 2 | 8 | 1 | 0.03405 |
| 1433991_x_at | Dbi | 101 | 1 | 48 | 3 | 0.03406 |
| 1417820_at | Tor1b | 73 | 3 | 11 | 1 | 0.0341 |
| 1429038_at | Dda1 | 33 | 2 | 100 | 5 | 0.03415 |
| 1421030_at | Zfp64 | 103 | 2 | 44 | 1 | 0.03435 |
| 1448200_at | Tcn2 | 30 | 2 | 97 | 6 | 0.0344 |
| 1419218_at | Vangl2 | 37 | 4 | 95 | 5 | 0.03455 |
| 1416013_at | Pld3 | 37 | 4 | 103 | 3 | 0.03457 |
| 1418195_at | Galnt10 | 102 | 3 | 43 | 4 | 0.03461 |
| 1419979_s_at | Creb3 | 90 | 9 | 28 | 1 | 0.03465 |
| 1416743_at | Uap1 | 64 | 1 | 10 | 1 | 0.03483 |
| 1453283_at | Pgm1 | 89 | 5 | 31 | 3 | 0.03584 |
| 1415971_at | Marcks | 103 | 3 | 52 | 3 | 0.03606 |
| 1423083_at | Rab33b | 105 | 3 | 44 | 2 | 0.03646 |
| 1425753_a_at | Ung | 96 | 4 | 43 | 3 | 0.03648 |
| 1426104_at | Mapk14 | 77 | 1 | 24 | 2 | 0.03768 |
| 1448901_at | Cpxm1 | 89 | 6 | 29 | 2 | 0.03831 |
| 1426694_at | 9030624J02Rik | 17 | 2 | 73 | 4 | 0.03843 |
| 1428488_at | Pigk | 53 | 1 | 110 | 2 | 0.03857 |
| 1456567_x_at | Grn | 98 | 3 | 29 | 1 | 0.03868 |
| 1454664_a_at | Eif5 | 115 | 1 | 59 | 3 | 0.03881 |
| 1437034_x_at | Marcks | 95 | 3 | 29 | 2 | 0.03885 |
| 1425148_a_at | Snx6 | 74 | 3 | 116 | 3 | 0.03892 |
| 1420337_at | Gbx2 | 52 | 3 | 109 | 2 | 0.03929 |
| 1419798_at | 2610019E17Rik | 64 | 2 | 8 | 1 | 0.04039 |
| 1450506_a_at | Aen | 89 | 5 | 32 | 3 | 0.04047 |
| 1423882_at | Rfwd3 | 26 | 3 | 91 | 6 | 0.0407 |
| 1448494_at | Gas1 | 49 | 1 | 101 | 1 | 0.04084 |
| 1423431_a_at | Mybbp1a | 14 | 1 | 70 | 5 | 0.04111 |
| 1427332_at | Med19 | 40 | 2 | 90 | 5 | 0.04138 |
| 1424503_at | Rab22a | 29 | 1 | 101 | 2 | 0.04169 |
| 1424591_at | 5830433M19Rik | 7 | 1 | 114 | 2 | 0.04192 |
| 1422208_a_at | Gnb5 | 85 | 5 | 23 | 3 | 0.04217 |
| 1415968_a_at | Kap | 97 | 2 | 30 | 2 | 0.04232 |
| 1424638_at | Cdkn1a | 93 | 3 | 35 | 3 | 0.04234 |
| 1415855_at | Kitl | 44 | 1 | 110 | 1 | 0.04277 |
| 1427908_at | Bnip1 | 64 | 3 | 6 | 1 | 0.04335 |
| 1417042_at | Slc37a4 | 104 | 2 | 43 | 2 | 0.04388 |
| 1416920_at | Rbm4 | 40 | 4 | 92 | 8 | 0.04395 |
| 1428468_at | 3110043O21Rik | 31 | 2 | 103 | 7 | 0.044 |
| 1434403_at | Spred2 | 53 | 2 | 107 | 3 | 0.04415 |
| 1437741_at | Rab21 | 73 | 3 | 12 | 1 | 0.04426 |
| 1437325_x_at | Aldh18a1 | 37 | 4 | 101 | 1 | 0.04435 |
| 1421906_at | Med1 | 64 | 2 | 11 | 1 | 0.04474 |
| 1448547_at | Rassf3 | 90 | 6 | 31 | 2 | 0.04524 |
| 1419613_at | Col7a1 | 30 | 1 | 101 | 6 | 0.04555 |
| 1419034_at | Csnk2a1 | 68 | 1 | 11 | 1 | 0.04625 |
| 1451421_a_at | Rogdi | 29 | 1 | 100 | 4 | 0.04639 |
| 1423986_a_at | Shisa5 | 95 | 4 | 31 | 2 | 0.04706 |
| 1416387_at | Pip4k2c | 92 | 3 | 19 | 2 | 0.04738 |
| 1451335_at | Plac8 | 18 | 1 | 84 | 7 | 0.04767 |
| 1451543_at | Fbxo21 | 30 | 1 | 97 | 3 | 0.04783 |
| 1427087_at | Luc7l2 | 55 | 2 | 110 | 3 | 0.04799 |
| 1417525_at | Hand1 | 88 | 3 | 29 | 1 | 0.04804 |
| 1423746_at | Txndc5 | 32 | 3 | 88 | 6 | 0.04861 |
| 1423298_at | Add3 | 42 | 2 | 98 | 3 | 0.0487 |
| 1452118_at | Rrp1b | 71 | 3 | 15 | 2 | 0.04922 |
| 1452582_at | Galm | 32 | 2 | 109 | 2 | 0.04923 |
| 1427253_s_at | Suz12 | 33 | 3 | 92 | 7 | 0.04923 |
| 1448438_at | Derl2 | 41 | 2 | 89 | 6 | 0.04929 |
| 1427014_at | Dennd4b | 98 | 6 | 32 | 2 | 0.04938 |
| 1450209_at | Hoxd4 | 12 | 1 | 62 | 3 | 0.04973 |
| 1419176_at | Vps37a | 101 | 2 | 37 | 5 | 0.04973 |
| 1436639_at | Bola2 | 101 | 3 | 36 | 5 | 0.04985 |
