# Supplementary material for: The precise timeline of transcriptional regulation reveals causation in mouse somitogenesis network
Source: BMC Dev Biol. 2013 Dec 5;13:42. doi: 10.1186/1471-213X-13-42 (PMC4235037; doi:10.1186/1471-213X-13-42)
Supplement: Additional file 10: Table S3 — The list of genes with one peak of expression. The timing of genes found with one peak of expression, ranked according to their LS p-value and the regularity of their profile. Times in minutes assume a 2 h periodicity for every transcript and errors are computed by adding to the original transcript source of noise typically found in microarray experiments (see text). [file 1471-213X-13-42-S10.docx]

**Table S3: Timing of genes with one peak of expression**.

| **Probeset** | **Gene** | **Time (min)** | **Error (min)** | **LS p-value** |
| --- | --- | --- | --- | --- |
| 1420360_at | Dkk1 | 22 | 2 | 0.00017 |
| 1427600_at | Tnfrsf19 | 26 | 2 | 0.00022 |
| 1436845_at | Axin2 | 20 | 2 | 0.00041 |
| 1418102_at | Hes1 | 88 | 3 | 0.00086 |
| 1417937_at | Dact1 | 31 | 3 | 0.00097 |
| 1422914_at | Sp5 | 23 | 2 | 0.00115 |
| 1430111_a_at | Bcat1 | 50 | 6 | 0.00122 |
| 1424942_a_at | Myc | 20 | 2 | 0.00185 |
| 1417065_at | Egr1 | 88 | 5 | 0.00201 |
| 1456010_x_at | Hes5 | 80 | 3 | 0.00226 |
| 1425895_a_at | Id1 | 82 | 4 | 0.00233 |
| 1437666_x_at | Ubc | 19 | 1 | 0.00248 |
| 1416029_at | Klf10 | 97 | 6 | 0.00279 |
| 1454904_at | Mtm1 | 80 | 2 | 0.0031 |
| 1415999_at | Hey1 | 64 | 4 | 0.00325 |
| 1420494_x_at | Ubc | 113 | 1 | 0.00342 |
| 1421948_a_at | Ccdc123 | 36 | 3 | 0.00347 |
| 1448378_at | Fscn1 | 113 | 3 | 0.00384 |
| 1425029_a_at | Mboat2 | 85 | 5 | 0.00392 |
| 1416911_a_at | Akirin1 | 7 | 1 | 0.00417 |
| 1431753_x_at | Urm1 | 43 | 3 | 0.00765 |
| 1418436_at | Stx7 | 97 | 8 | 0.00907 |
| 1427988_s_at | Safb2 | 35 | 3 | 0.00923 |
| 1425212_a_at | Tnfrsf19 | 20 | 1 | 0.00948 |
| 1449019_at | Akap1 | 107 | 4 | 0.00952 |
| 1450242_at | Tlr5 | 103 | 3 | 0.00964 |
| 1449169_at | Has2 | 24 | 3 | 0.00968 |
| 1436343_at | Chd4 | 74 | 4 | 0.01024 |
| 1448650_a_at | Pole | 35 | 4 | 0.01027 |
| 1436820_at | Kctd11 | 103 | 6 | 0.01053 |
| 1417986_at | Nrarp | 65 | 3 | 0.01072 |
| 1452041_at | Klhl26 | 6 | 1 | 0.01102 |
| 1418835_at | Phlda1 | 30 | 2 | 0.01106 |
| 1419180_at | Bcl9l | 98 | 8 | 0.01111 |
| 1423351_at | Mrpl1 | 114 | 6 | 0.01137 |
| 1435476_a_at | Fcgr2b | 101 | 5 | 0.01144 |
| 1426461_at | Ugp2 | 103 | 3 | 0.0116 |
| 1425579_at | Gfra2 | 97 | 4 | 0.01212 |
| 1435493_at | Dsp | 104 | 2 | 0.01246 |
| 1416773_at | Wee1 | 107 | 2 | 0.01255 |
| 1420441_at | Cenpc1 | 112 | 5 | 0.0127 |
| 1426938_at | Nova1 | 108 | 5 | 0.01279 |
| 1448557_at | Fam13c | 89 | 4 | 0.01282 |
| 1422999_at | Map3k14 | 113 | 5 | 0.01292 |
| 1437715_x_at | Apex1 | 91 | 9 | 0.01295 |
| 1455678_at | Sema4b | 76 | 2 | 0.01371 |
| 1437100_x_at | Pim3 | 102 | 6 | 0.01381 |
| 1417548_at | Sart3 | 61 | 1 | 0.01402 |
| 1451912_a_at | Fgfrl1 | 61 | 2 | 0.01419 |
| 1439421_x_at | Cbx3 | 80 | 6 | 0.01428 |
| 1431419_at | Prelid1 | 7 | 1 | 0.01441 |
| 1417027_at | Trim2 | 113 | 2 | 0.01486 |
| 1456464_x_at | Syt11 | 109 | 3 | 0.01501 |
| 1448880_at | Ube2l3 | 8 | 1 | 0.01532 |
| 1438206_a_at | Sys1 | 19 | 2 | 0.01851 |
| 1427905_at | 1810063B07Rik | 112 | 8 | 0.01862 |
| 1434624_x_at | Rps9 | 7 | 1 | 0.01867 |
| 1435494_s_at | Dsp | 104 | 1 | 0.0187 |
| 1449678_at | Tnpo3 | 109 | 3 | 0.01909 |
| 1451408_at | Trub2 | 8 | 1 | 0.01936 |
| 1428258_at | 2010107E04Rik | 19 | 1 | 0.01942 |
| 1416823_a_at | Osbpl1a | 98 | 10 | 0.01951 |
| 1420947_at | Atrx | 62 | 5 | 0.01955 |
| 1416039_x_at | Cyr61 | 20 | 2 | 0.01956 |
| 1448829_at | Smc6 | 113 | 5 | 0.01967 |
| 1425336_x_at | H2-K1 | 7 | 1 | 0.01988 |
| 1448985_at | Dusp22 | 7 | 1 | 0.01998 |
| 1439111_at | Gm19597 | 103 | 3 | 0.02029 |
| 1451569_at | Nr2c2 | 103 | 7 | 0.02029 |
| 1416874_a_at | Paf1 | 25 | 2 | 0.02062 |
| 1431125_a_at | Tars2 | 43 | 3 | 0.02063 |
| 1434391_at | AI503316 | 100 | 5 | 0.02078 |
| 1424569_at | Ddx46 | 40 | 3 | 0.02104 |
| 1421556_at | Serpina3a | 53 | 1 | 0.02143 |
| 1423356_at | Snap29 | 102 | 3 | 0.02147 |
| 1460246_at | Mecp2 | 102 | 3 | 0.02166 |
| 1426728_x_at | Ptdss2 | 74 | 3 | 0.02176 |
| 1433666_s_at | Vps41 | 89 | 3 | 0.02222 |
| 1460377_a_at | Tmem8 | 11 | 1 | 0.02257 |
| 1419917_s_at | Tmed7 | 50 | 2 | 0.0226 |
| 1420397_a_at | Spen | 7 | 1 | 0.02267 |
| 1449849_a_at | Fbxl6 | 77 | 8 | 0.02313 |
| 1417428_at | Gng3 | 115 | 6 | 0.02332 |
| 1434326_x_at | Coro2b | 103 | 4 | 0.0234 |
| 1427213_at | Pfkfb1 | 114 | 1 | 0.0234 |
| 1429451_at | 2610301B20Rik | 114 | 1 | 0.02367 |
| 1425831_at | Zfp101 | 44 | 2 | 0.02378 |
| 1425731_at | Ankrd24 | 108 | 5 | 0.02379 |
| 1452042_a_at | Tmem144 | 100 | 4 | 0.0244 |
| 1420643_at | Lfng | 76 | 1 | 0.02458 |
| 1428195_at | Ahcyl2 | 87 | 7 | 0.02491 |
| 1454675_at | Thra | 95 | 4 | 0.02492 |
| 1423792_a_at | Cmtm6 | 106 | 5 | 0.02506 |
| 1427987_at | Safb2 | 29 | 2 | 0.02525 |
| 1449167_at | Epb4.1l4a | 71 | 1 | 0.0261 |
| 1433835_at | Ppp3cb | 4 | 1 | 0.02616 |
| 1425674_a_at | Ssu72 | 11 | 1 | 0.02833 |
| 1448420_a_at | Fbxl12 | 98 | 9 | 0.02843 |
| 1419834_x_at | Mark1 | 92 | 3 | 0.02895 |
| 1451898_a_at | Sema6c | 97 | 4 | 0.02895 |
| 1426180_a_at | Smr2 | 112 | 2 | 0.02906 |
| 1423701_at | Coasy | 43 | 3 | 0.0292 |
| 1437712_x_at | Exosc4 | 62 | 4 | 0.02929 |
| 1438133_a_at | Cyr61 | 28 | 2 | 0.02952 |
| 1451618_at | Rho | 115 | 1 | 0.03034 |
| 1418153_at | Lama1 | 49 | 4 | 0.0306 |
| 1425561_at | Trnt1 | 113 | 3 | 0.03071 |
| 1419170_at | Fam174a | 106 | 3 | 0.03079 |
| 1451323_at | Zfp7 | 82 | 10 | 0.03085 |
| 1419163_s_at | Dnajc3 | 85 | 2 | 0.03312 |
| 1460654_at | Slc30a3 | 4 | 1 | 0.03325 |
| 1421230_a_at | Msi2 | 80 | 2 | 0.03341 |
| 1427096_s_at | Ssr4 | 104 | 3 | 0.03346 |
| 1424781_at | Reep3 | 101 | 8 | 0.03364 |
| 1459917_at | Ggnbp2 | 77 | 5 | 0.03411 |
| 1415834_at | Dusp6 | 67 | 5 | 0.03438 |
| 1418669_at | Hspg2 | 94 | 4 | 0.03449 |
| 1425517_s_at | Ogt | 107 | 3 | 0.03466 |
| 1449474_a_at | Nelf | 113 | 4 | 0.03474 |
| 1417959_at | Pdlim7 | 41 | 2 | 0.03475 |
| 1452306_at | Zfyve26 | 115 | 5 | 0.03554 |
| 1455581_x_at | Gm20559 | 107 | 5 | 0.03563 |
| 1454689_at | Srrm1 | 7 | 1 | 0.03572 |
| 1427107_at | Slc16a11 | 96 | 6 | 0.03583 |
| 1426275_a_at | Uxs1 | 12 | 1 | 0.03611 |
| 1417316_at | Acot13 | 77 | 7 | 0.03612 |
| 1451056_at | Psmd7 | 43 | 3 | 0.0363 |
| 1451412_a_at | Ift20 | 42 | 2 | 0.03646 |
| 1424184_at | Acadvl | 42 | 4 | 0.03661 |
| 1420728_at | Krt32 | 11 | 1 | 0.03758 |
| 1438562_a_at | Ptpn2 | 104 | 3 | 0.03761 |
| 1443762_s_at | Sbf2 | 103 | 3 | 0.03819 |
| 1427062_at | Rbbp8 | 94 | 4 | 0.03837 |
| 1417018_at | Efemp2 | 43 | 1 | 0.03844 |
| 1418634_at | Notch1 | 53 | 5 | 0.03858 |
| 1425270_at | Kif1b | 79 | 3 | 0.03871 |
| 1426790_at | Ssrp1 | 36 | 2 | 0.03885 |
| 1456488_at | Wdr33 | 100 | 4 | 0.03908 |
| 1432249_a_at | Ercc8 | 7 | 1 | 0.03911 |
| 1418512_at | Stk3 | 7 | 1 | 0.03926 |
| 1452187_at | Rbm5 | 94 | 3 | 0.0394 |
| 1424872_at | Zfp932 | 54 | 5 | 0.03948 |
| 1420570_x_at | Tcl1b3 | 90 | 6 | 0.03984 |
| 1417871_at | Hsd17b7 | 110 | 2 | 0.04077 |
| 1460698_a_at | Sec11c | 40 | 3 | 0.04082 |
| 1448119_at | Bpgm | 38 | 4 | 0.04088 |
| 1424373_at | Armcx3 | 89 | 8 | 0.04173 |
| 1437032_x_at | Rbm14 | 106 | 2 | 0.04214 |
| 1448724_at | Cish | 104 | 1 | 0.0424 |
| 1422546_at | Ilf3 | 74 | 4 | 0.04241 |
| 1432419_a_at | Mob2 | 31 | 3 | 0.04261 |
| 1426801_at | Sept8 | 47 | 2 | 0.04271 |
| 1460694_s_at | Svil | 113 | 5 | 0.04353 |
| 1439371_x_at | Timm44 | 113 | 2 | 0.04373 |
| 1422270_a_at | Il6ra | 113 | 3 | 0.04376 |
| 1419485_at | Foxc1 | 104 | 6 | 0.04391 |
| 1448289_at | Crmp1 | 27 | 2 | 0.04398 |
| 1421929_at | Epha4 | 89 | 6 | 0.04408 |
| 1426739_at | Donson | 7 | 1 | 0.0442 |
| 1431832_x_at | Exosc10 | 37 | 5 | 0.04426 |
| 1427151_at | Qser1 | 6 | 1 | 0.04426 |
| 1423724_at | Zwint | 58 | 5 | 0.04456 |
| 1450918_s_at | Src | 4 | 1 | 0.04461 |
| 1415988_at | Hdlbp | 102 | 4 | 0.04484 |
